# ﻿Unveiling fungal diversity associated with coffee trees in China using a polyphasic approach and a global review of coffee saprobic fungi

**DOI:** 10.3897/imafungus.16.144874

**Published:** 2025-03-10

**Authors:** Li Lu, Samantha C. Karunarathna, Kunhiraman C. Rajeshkumar, Abdallah M. Elgorban, Ruvishika S. Jayawardena, Sinang Hongsanan, Nakarin Suwannarach, Jaturong Kumla, Yin-Ru Xiong, Kevin D. Hyde, Mei-Yan Han, De-Ge Zheng, Qiang Li, Dong-Qin Dai, Saowaluck Tibpromma

**Affiliations:** 1 Center for Yunnan Plateau Biological Resources Protection and Utilization, Key Laboratory of Yunnan Provincial Department of Education of the Deep-Time Evolution on Biodiversity from the Origin of the Pearl River, College of Biology and Food Engineering, Qujing Normal University, Qujing, Yunnan 655011, China; 2 Center of Excellence in Fungal Research, Mae Fah Luang University, Chiang Rai 57100, Thailand; 3 School of Science, Mae Fah Luang University, Chiang Rai 57100, Thailand; 4 Center of Excellence in Microbial Diversity and Sustainable Utilization, Faculty of Science, Chiang Mai University, Chiang Mai 50200, Thailand; 5 National Fungal Culture Collection of India (NFCCI), Biodiversity & Palaeobiology (Fungi) Group, Agharkar Research Institute, Gopal Ganesh Agarkar Road, Pune 411 004, Maharashtra, India; 6 Department of Botany and Microbiology, College of Science, King Saud University, PO 2455, Riyadh 11451, Saudi Arabia; 7 Department of Biology, Faculty of Science, Chiang Mai University, Chiang Mai 50200, Thailand; 8 Innovative Institute for Plant Health, College of Agriculture and Biology, Zhongkai University of Agriculture and Engineering, Guangzhou 510225, Guangdong, China

**Keywords:** *Coffeaarabica*, Fungal diversity, new taxa, southwest China, taxonomy

## Abstract

Arabica coffee (*Coffeaarabica*) is the most cultured and popular coffee bean in today’s world. Yunnan Province is well known as China’s largest arabica coffee cultivation region. Fungi represent an important group of microorganisms associated with coffee, profoundly influencing its yield and quality. In this study, twelve fungal collections growing on dead and decaying twigs of coffee were collected and isolated to systematically document microfungi associated with coffee plants in Yunnan Province. Ten novel species, each representing a unique family within *Pleosporales*, were identified and introduced, based on comprehensive morphological analyses and multigene phylogenetic studies. The ten new species belong to the families *Bambusicolaceae*, *Didymellaceae*, *Didymosphaeriaceae*, *Longiostiolaceae*, *Lophiostomataceae*, *Massarinaceae*, *Neomassariaceae*, *Occultibambusaceae*, *Roussoellaceae* and *Thyridariaceae* with each family containing one new species. Macro- and micro-characteristics, descriptions and phylogenetic trees indicating the placement of the new taxa are provided. In addition, pairwise homoplasy index (PHI) test results and morphological comparisons between the new species and closely-related taxa are given. This study also establishes a comprehensive global inventory of saprobic fungi associated with coffee, which is intended to help researchers and professionals worldwide with practical information. This research enhances the understanding of coffee-associated fungal diversity in China and underscores the importance of introducing new saprobic fungal taxa related to coffee.

## ﻿Introduction

*Coffea* L. (*Rubiaceae* Juss.) is the world’s 124^th^ most-traded product and the second most popular beverage worldwide (Adhikari et al. 2020; ALAsmari et al. 2020). The coffee industry is a powerhouse, contributing to the economies of both exporting and importing countries. Its annual income is estimated to exceed $200 billion, providing livelihoods for around 100 million families (Adugna 2024; Freitas et al. 2024). The genus *Coffea* boasts over 120 species, but only two, Arabica coffee (*Coffeaarabica* L., 75%) and Robusta coffee (*Coffeacanephora* Pierre ex A. Froehner, 25%), hold significant economic importance, making them the key players in the industry (Lu et al. 2022e; Gole and Seyoum 2024). China now ranks as the 13^th^ largest coffee producer globally (https://colipsecoffee.com/blogs/coffee/countries) and the cultivation areas are mainly distributed in Yunnan and Hainan Provinces, with Yunnan’s coffee planting areas, production and agricultural output value accounting for more than 98% of the country’s total, making it a significant contributor to China’s economy (Neilson and Wang 2019; Zhang et al. 2021). Arabica coffee is the most widely grown and popular coffee in China, especially in Yunnan Province (Rigal et al. 2020; Zhang et al. 2021). It is worth noting that fungi are a common presence during the different stages of coffee processing, indicating the need for further research and development in this area (De Bruyn et al. 2017).

There are an estimated 2.5 million species of fungi, but only about 165,000 have been described thus far (Hyde et al. 2022; Niskanen et al. 2023; Kraisitudomsook et al. 2024; https://www.indexfungorum.org (accessed on 12 January 2025)). The first coffee-associated microorganism described was the arbuscular mycorrhizal fungi (AMF) colonising the roots of *Coffeaarabica* and *C.liberica* Hiern (Janse 1897). Fungi associated with coffee exist in different life modes, including endophytes, pathogens and saprobes (Duong et al. 2020; Lu et al. 2022a). Based on the review of coffee-associated fungi, 648 species have been reported (Lu et al. 2022a). Amongst them, pathogens are the most widely studied fungi in coffee as their infection can impact coffee production and quality, with 295 species reported (Lu et al. 2022a). Coffee endophytes have also been extensively studied for their potential as biocontrol agents (Quesada-Moraga et al. 2014; Monteiro et al. 2020; Lu et al. 2022b), with 138 species reported (Lu et al. 2022a). However, the potential of using the saprobic microflora isolated from coffee berry surfaces to manage coffee berry disease, as shown by Masaba (1993) and Gichuru (2005), is an intriguing area of research, despite only 30 saprobic fungi being reported worldwide (Lu et al. 2022a). There were only a few reports on coffee-associated saprobic fungi in China before 2020. After 2020, there has been a significant increase in the reports of saprobic fungi on coffee in China, keeping the researchers informed and up-to-date (Lu et al. 2022c, d, e; Hyde et al. 2023; Lu et al. 2024; Liu et al. 2024).

The Greater Mekong Subregion (GMS) region in Yunnan Province has an enormous fungal diversity (Hyde et al. 2018; Chethana et al. 2021). In this study, we conducted a comprehensive study of microfungi collected from the GMS. Our efforts have led to the discovery of ten new species of *Pleosporales* Luttrell ex M.E. Barr, associated with dead branches of *Coffeaarabica*, collected from Baoshan, Dali, Lincang, Pu’er and Xishuangbanna in Yunnan Province, China. These new species, distributed in ten different genera and families in *Pleosporales*, not only showcase the diversity of this group of fungi, but also have the potential to significantly impact future research on coffee-associated saprobic fungi in Yunnan Province. We provide full descriptions, photo plates of macro- and micro-morphological characteristics and comparisons with closely-related taxa and phylogenetic trees to indicate the placement of these meticulously researched new taxa. Furthermore, we have provided a comprehensive global list of 62 coffee-associated saprobic fungi (See Suppl. material 1: table S1), including the year, host and country. This global perspective is crucial for understanding these fungi’s full diversity and distribution. In addition, the diversity of coffee-associated saprobic fungi was analysed in the past four years and compared to previous studies. This comparison is not just a comparison; it is a testament to the thoroughness and comprehensive nature of our research, providing a valuable benchmark for our current findings.

## ﻿Methods

### ﻿Literature survey

To comprehensively review the diversity of saprobic fungi associated with coffee trees globally, we conducted a literature survey using academic databases, including the esteemed Google Scholar and the USDA Fungal Databases (https://fungi.ars.usda.gov/). Keywords such as “coffee”, “fungi” and “saprobic” were employed to identify relevant studies. The data we extracted on fungal species, geographic locations and host plants were compiled into Suppl. material 1: table S1.

### ﻿Sample collection, morphological observation and isolation

Yunnan, one of China’s most biodiverse provinces, is a key region for studying fungal communities and their unique ecological roles in coffee ecosystems. We randomly obtained dead and decaying twigs of coffee plant samples with fungal fruiting bodies from coffee plantations in subtropical regions (Baoshan, Dali, Lincang and Pu’er) to tropical regions (Xishuangbanna) of Yunnan Province, China, from 2020 to 2022, noting important collection information (Rathnayaka et al. 2024). Saprobic fungi introduced in this study were isolated from Arabica coffee. Samples were put in paper bags, taken to the mycology laboratory at Qujing Normal University and kept in boxes until observed. The vertical portions of the fruiting body structures were prepared for photomicrography. The measurements were processed in Tarosoft (R) Image Frame Work v. 0.9.7 and photographic plates were made in Adobe Photoshop CC (2018). The single spore isolation method described by Senanayake et al. (2020) was followed to obtain pure cultures on potato dextrose agar (PDA) with antibiotic amoxicillin to prevent bacterial growth (for 500 ml PDA, 2 g of amoxicillin powder was added: Renhe brand amoxicillin capsules). Herbarium specimens were deposited in Kunming Institute of Botany Academia Sinica (HKAS) and Mycological Herbarium of Zhongkai University of Agriculture and Engineering (MHZU), living cultures growing on PDA were deposited in Kunming Institute of Botany Culture Collection (KUNCC) and culture collection of Zhongkai University of Agriculture and Engineering (ZHKUCC). Faces of fungi (FoF) numbers and Index Fungorum (IF) numbers were obtained, as explained in Jayasiri et al. (2015) and Index Fungorum (https://www.indexfungorum.org, accessed on 12 January 2025).

### ﻿Molecular studies

The Biospin Fungus Genomic DNA Extraction Kit-BSC14S1 (BioFlux, China) was used to extract genomic DNA from fresh fungal mycelia cultivated on PDA for two weeks, following the manufacturer’s instructions. The polymerase chain reaction (PCR) amplification was conducted following the method described by Lu et al. (2022b). This study used six loci; their primers and amplification reactions are shown in Suppl. material 1: tables S2, S3. The PCR products were sequenced at Sango Biotechnology Co., Ltd. (Shanghai, China). All sequences generated in this study were deposited in GenBank (https://www.ncbi.nlm.nih.gov/genbank/) and used for the phylogenetic analysis. Information on all sequences used for phylogenetic analyses is available in the supplementary materials (See Suppl. material 1: tables S4–S13). All the obtained alignments and phylogenetic trees were deposited in Figshare (https://figshare.com/).

### ﻿Phylogenetic analyses

Raw forward and reverse reads produced in this study were assembled with Geneious v. 9.1.2 (https://www.geneious.com) and initial identification was performed by BLASTn search in GenBank. The related sequence data were obtained from GenBank, based on the latest literature. Single gene sequence alignments were conducted with the online programme MAFFT v. 7.110 (https://mafft.cbrc.jp/alignment/server/). TrimAL v. 1.2 (http://trimal.cgenomics.org) was used to remove the uninformative gaps and ambiguous regions and Sequence Matrix v. 1.7.8 was used to concatenate the individual alignments (Vaidya et al. 2011). The fasta files were converted to Nexus files using AliView v. 1.28 (Larsson 2014). Maximum Likelihood (ML) and Bayesian Inference (BI) algorithms were used to perform phylogenetic analyses of the aligned sequences, as explained by Dissanayake et al. (2020), conducted on the CIPRES Science Gateway portal (https://www.phylo.org). A Maximum Likelihood analysis was performed using RAxML-HPC v.8, with rapid bootstrap analysis, followed by 1000 bootstrap replicates; the GTRGAMMA model was used for all partitions. The Bayesian analysis was used to evaluate posterior probabilities (PP) by MrBayes on XSEDE v. 3.2.7a; six simultaneous Markov Chains were run for two million generations and trees were sampled at every 200^th^ generation (resulting in 10,000 trees) and these chains stopped when all convergences met and the standard deviation fell below 0.01. The phylogenetic trees were viewed by FigTree v. 1.4.2 (Rambaut 2014) and edited using Microsoft Office PowerPoint (2020) and Adobe Illustrator CS6 (Adobe Systems Inc., USA). The bootstrap support values for ML equal to or greater than 70% and PP equal to or greater than 0.90 were mentioned above the nodes.

### ﻿Genealogical concordance phylogenetic species recognition analysis

Genealogical concordance phylogenetic species recognition analysis (GCPSR) by the pairwise homoplasy index (PHI) test was used to determine the recombination level within closely-related species (Bruen et al. 2006). The data were analysed using the software SplitsTree v. 4 (Bruen et al. 2006; Huson and Bryant 2006). The relationships between closely-related taxa were visualised by constructing split graphs from concatenated datasets using the LogDet transformation and split decomposition options. If the PHI test value is lower than (Φw) ≤ 0.05, it indicates significant recombination within the dataset. This is an important method to provide further evidence to justify a species.

## ﻿Results

### ﻿Global diversity of coffee-associated saprobic fungi

The literature survey revealed a limited diversity of saprobic fungi associated with coffee trees globally, with only 62 species reported worldwide, including ten new species described in this study. A total of 62 coffee-associated saprobic species are distributed in 18 orders, 37 families and 47 genera (See Suppl. material 1: table S1), highlighting the global reach of our research. Amongst 62 species, 31 coffee-associated saprobic fungi were recorded worldwide from 1980 to 2020 and another 31 species were reported from Yunnan Province, China, from 2021 to 2024. The discovery of 31 new species in Yunnan, China, from 2021 to 2024 is a testament to the potential novelty of coffee-associated fungi in this region, a finding that is sure to spark scientific curiosity.

**Figure 1.**
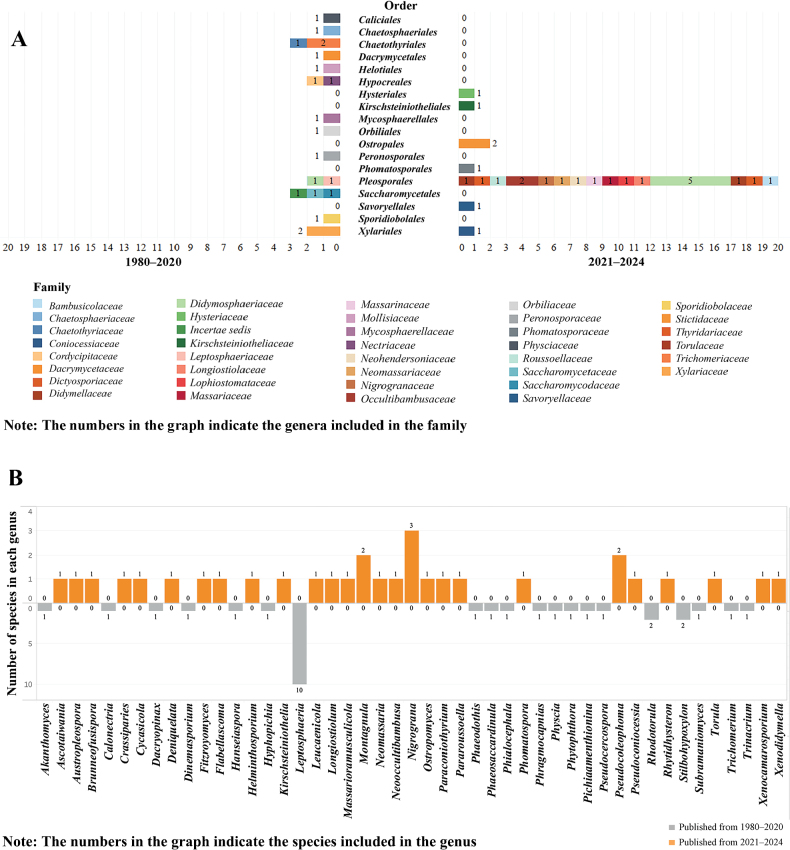
Analysis of the diversity of coffee-associated saprobic fungi from 1980 to 2020 (first period) and from 2021 to 2024 (Second period). **A** comparison of taxonomic distribution at the order and family levels between the two periods; **B** comparison of taxonomic distribution at the genus level between the two periods.

From 1980 to 2020, the 31 species reported were distributed across 13 orders, 18 families and 20 genera. In contrast, from 2021 to 2024, 31 species were reported across seven orders, 21 families and 27 genera (Fig. 1A, B). Additionally, no overlap in the species was reported between these two periods (Fig. 1B). This indicates that the diversity of coffee-associated saprobic fungi reported in Yunnan, China, is greater than that previously documented globally. This underscores the urgent and significant need for further exploration and study of saprobic fungi on coffee, a call to action for the scientific community.

Amongst the 62 species, the most frequently segregated order is *Pleosporales*, encompassing 17 families, 22 genera and 35 species (Fig. 1A). This paper introduces ten new taxa within this order, distributed across different genera and families. The discovery of these new taxa is significant as it not only adds to the diversity of coffee-associated saprobic fungi, but also underscores the need for further exploration and study in this field.

### ﻿Taxonomy

#### ﻿*Fungi*

***Ascomycota* Caval. Sm.**

***Dothideomycetes* O. E. Erikss. & Winka.**

***Pleosporales* Luttr. ex M.E. Barr, Prodr. Cl. Loculoasc. (Amherst): 67 (1987).**

##### *Bambusicolaceae* D.Q. Dai & K.D. Hyde, Fungal Diversity 63 (1): 49 (2013).

###### ﻿ Leucaenicola

Taxon classificationFungiPleosporalesBambusicolaceae

Jayasiri, E.B.G. Jones & K.D. Hyde, Mycosphere 10 (1): 37 (2019).

05B46DD9-A75B-56A1-8FB2-B15D6330643D

####### Notes.

*Leucaenicola* was introduced by Jayasiri et al. (2019) to accommodate two new coelomycetous species, with *L.aseptata* Jayasiri, E.B.G. Jones & K.D. Hyde, as the type species. *Leucaenicola* contains five species; only their anamorph has been documented (Jayasiri et al. 2019; Ariyawansa et al. 2020a, b; Hyde et al. 2024). Herein, we introduce a new species of *Leucaenicola* with both anamorph and teleomorph and this is the first report of *Leucaenicola* from Arabica coffee plants.

###### ﻿ Leucaenicola coffeae

Taxon classificationFungiPleosporalesBambusicolaceae

L. Lu & Tibpromma sp. nov.

E306F86C-A8F5-5F0C-99AC-553C61B33021

Index Fungorum number: IF90099

Facesofungi number: FoF14724

Fig. 2

####### Etymology.

The species epithet “*coffeae*” refers to the host plant genus “*Coffea*” from which the fungus was isolated.

####### Diagnosis.

Differs from *L.phraeana* Jayasiri, E.B.G. Jones & K.D. Hyde, by the brown and large conidia (5–6 × 2.5–3.5 μm vs. 3–4 × 1.5–2 μm) and distinct guttules.

####### Holotype.

HKAS 137605.

####### Description.

***Saprobic*** on decaying branch of *C.arabica*. Teleomorph: ***Ascomata*** 200–350 high × 200–300 µm diam. (x− = 239 × 273 µm), solitary or scattered, immersed, raised as brown to black spots on the substrate, globose to subglobose, coriaceous, uniloculate with ostioles. ***Peridium*** 25–35 μm wide (x− = 30 μm, n = 20), thin-walled, composed of dark brown and 2–4 layers of ***textura angularis*** cells, with the basal part composed of thinner, hyaline, smaller cells. ***Hamathecium*** 1.5–3.5 µm wide (x− = 2.7 µm, n = 20), dense, comprising numerous pseudoparaphyses, filamentous, hyaline, cellular, branched, with distinct septa. ***Asci*** 40–80 × 8–15 µm (x− = 57 × 9.6 µm, n = 20), 4–8-spored, bitunicate, fissitunicate, cylindrical, long-stalked with club-shape, apically rounded, with a shallow ocular chamber. ***Ascospores*** 15–25 × 4–8 µm (x− = 21 × 6 µm, n = 30), overlapping, uniseriate to biseriate, fusiform to ellipsoidal, straight, hyaline, mainly 1-septate, sometimes 2–3 septate, constricted at the centre septa, conical at both ends, upper cell wider than the lower cell, guttulate, mucilaginous sheath. Anamorph on PDA: ***Mycelium*** 1.5–2.5 μm broad (x− = 2.2 μm, n = 20), hyaline, septate, branched. ***Conidia*** 5–6 × 2.5–3.5 μm (x− = 5.6 × 3.1 μm, n = 30), ellipsoidal to cylindrical, hyaline when young, brown when mature, thin and smooth-walled, aseptate, with 1–2-guttules.

####### Culture characteristics.

Ascospores germinating on PDA within 24 h. Colonies reached 3.5 cm in diameter after one month at 25 °C. Colonies circular, slightly fluffy with an entire margin, white; the reverse is white to yellowish. After four months, conidia mass formed as globose to subglobose, dark brown to black spots in culture.

####### Materials examined.

China • Yunnan Province, Pu’er, on a decaying branch of *Coffeaarabica* (*Rubiaceae*) (23°43'01"N, 101°73'90"E, 1085 m alt.), 18 November 2020, LiLu, MJ-C8 (HKAS 137605, holotype), isotype MHZU 23-0058, ex-type living culture KUNCC 24-18335 = KUNCC 24-18336, ex-isotype living culture ZHKUCC 23-0626 = ZHKUCC 23-0627.

####### Notes.

**Figure 2.**
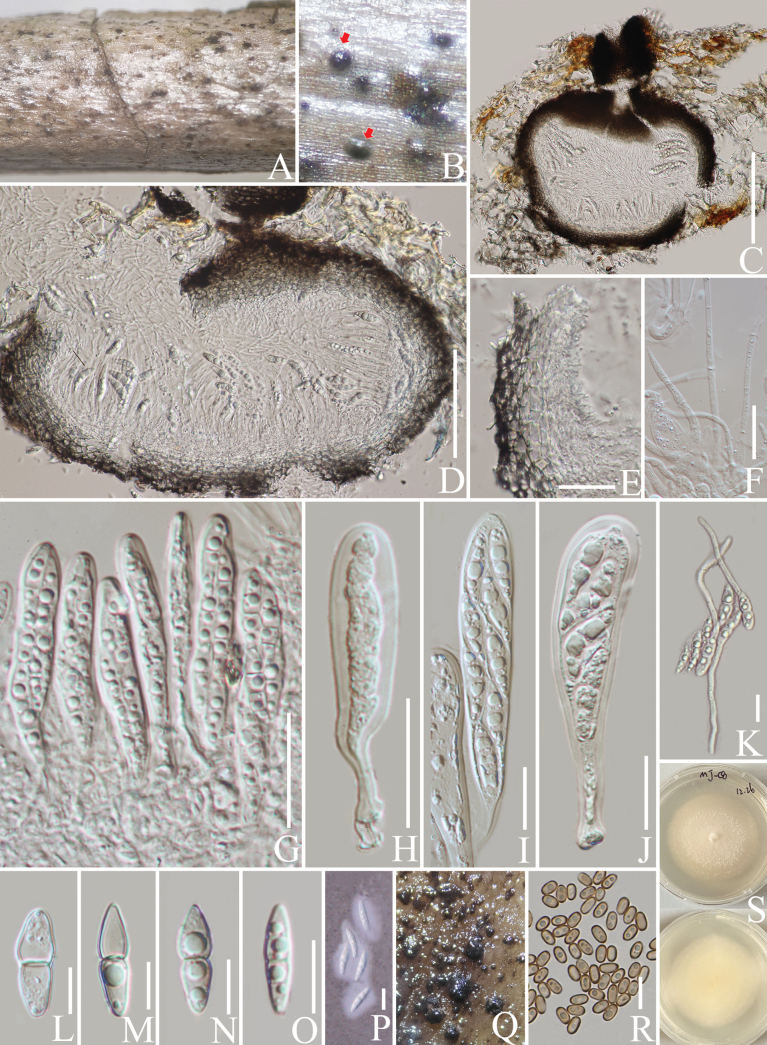
*Leucaenicolacoffeae* (HKAS 137605, holotype). **A, B** appearance of ascomata on a decaying branch of *C.arabica*; **C, D** longitudinal section of ascomata; **E** peridium wall; **F** pseudoparaphyses; **G–J** asci; **K** germinated ascospores; **L–O** ascospores; **P** ascospores stained with Indian ink; **Q** conidia mass in culture after four months; **R** conidia; **S** culture on PDA from obverse and reverse. Scale bars: 150 μm (**C, D**); 50 μm (**E**); 100 μm (**F**); 20 μm (**G–K**); 10 μm (**L–P, R**).

**Figure 3.**
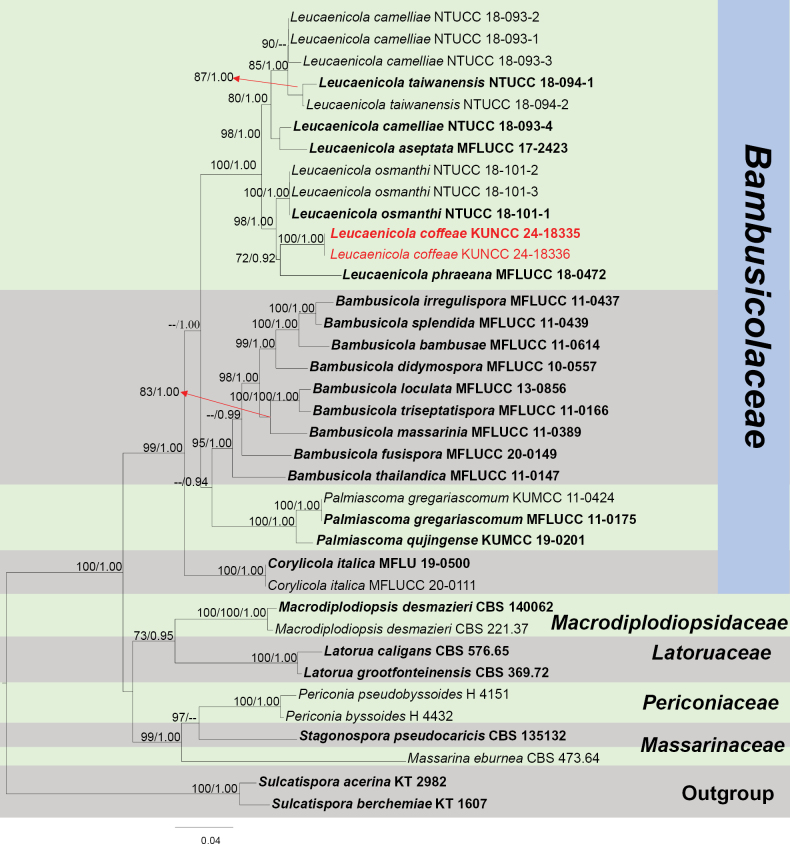
Phylogram generated from the best scoring RAxML tree, based on a combined ITS LSU, SSU, *RPB*2 and *TEF*1-α sequence dataset. *Sulcatisporaacerina* Kaz. Tanaka & K. Hiray., (KT 2982) and *S.berchemiae* Kaz. Tanaka & K. Hiray., (KT 1607) are selected as the outgroup taxa. Bootstrap support values for ML equal to or greater than 70% and PP equal to or greater than 0.90 are indicated at the nodes as ML/PP. All type strains are in bold and newly-generated sequences are in red.

Based on the multi-gene sequence analysis, *Leucaenicolacoffeae* forms an independent lineage allied to *L.phraeana* (Fig. 3). In the NCBI BLASTn searches, the ITS, LSU, SSU and *TEF*1-α sequences are similar to *L.camelliae* Ariyaw., I. Tsai & Thambugala, (MT112301, 98%), (MT071278, 90%), (MT071229, 99.9%) and (MT374091, 97%), respectively, while *RPB*2 is 97% similar to *L.osmanthi* Ariyaw., I. Tsai & Thambugala, (MN915020). *Leucaenicolacoffeae* is the first species with a teleomorph known in *Leucaenicola*. The teleomorph characteristics of *L.coffeae* conform to the basic concept of *Bambusicolaceae*. The anamorph can be distinguished from *L.phraeana* by the brown and large conidia (5–6 × 2.5–3.5 μm vs. 3–4 × 1.5–2 μm) and distinct guttules (Fig. 2; Jayasiri et al. (2019)). Based on nucleotide comparisons, *L.coffeae* (ZHKUCC 23-0626) differs from *L.phraeana* (MFLUCC 18-0472) by 102/418 bp (24%, 3 gaps) in ITS, 80/844 bp (9%, without gaps) in LSU, 2/1017 bp (0.2%, without gaps) in SSU, 16/920 bp (1.7%, without gaps) in *TEF*1-α and 30/1036 bp (2.9%, without gaps) in *RPB*2; and differs from *L.osmanthi* (NTUCC 18-101-3) by 10/620 bp (1.6%, without gaps) in ITS, 78/756 bp (10%, without gaps) in LSU, 2/780 bp (0.2%, without gaps) in SSU, 21/816 bp (2.5%, without gaps) in *TEF*1-α and 26/818 bp (3%, without gaps) in *RPB*2. In addition, the PHI test results (Fig. 22a) revealed no significant recombination relationships between *L.coffeae* and its phylogenetically related taxa within the dataset. Therefore, we introduce *L.coffeae* as a new species in *Leucaenicola* with the first record of a teleomorph associated with the decaying branch of *C.arabica*.

##### *Didymosphaeriaceae* Munk, Dansk botanisk Arkiv 15 (2): 128 (1953).

###### ﻿ Montagnula

Taxon classificationFungiPleosporalesDidymosphaeriaceae

Berl., Icones Fungorum. Pyrenomycetes 2: 68 (1896).

4BA23DEB-447B-550B-9D36-CF20C5AD1CF7

####### Notes.

Berlese (1896) introduced *Montagnula* typified by *M.infernalis* (Niessl) Berl. Most species of *Montagnula* have been found on dead leaves and twigs by their teleomorph with a wide geographical and host distribution (Tibpromma et al. 2018; Mapook et al. 2020; Du et al. 2021). Only *M.cylindrospora* Valenz.-Lopez, Cano, Guarro & Stchigel, has been reported as a coelomycete (Crous et al. 2020). This study introduces a novel species from an Arabica coffee plant in Yunnan Province, China.

###### ﻿ Montagnula coffeae

Taxon classificationFungiPleosporalesDidymosphaeriaceae

L. Lu & Tibpromma sp. nov.

34CAA4C4-DC5E-5F03-8ACF-EF437007DC07

Index Fungorum number: IF900999

Facesofungi number: FoF14725

Fig. 4

####### Etymology.

The species epithet “*coffeae*” refers to the host plant genus “*Coffea*” from which the fungus was isolated.

####### Diagnosis.

Differs from *M.appendiculata* (Aptroot) Wanas., E.B.G. Jones & K.D. Hyde, by 6–8-spored asci, differing from *M.chromolaenae* Mapook & K.D. Hyde, by guttulate ascospores and differing from *M.chiangraiensis* Mapook & K.D. Hyde, by the presence of appendages on its ascospores.

####### Holotype.

HKAS 137611.

####### Description.

***Saprobic*** on decaying branch of *C.arabica*. Teleomorph: ***Ascomata*** 120–180 µm high × 150–220 µm diam. (x− = 150 × 183 µm, n = 15), immersed, separate beneath a clypeus or sometimes gregarious beneath fused clypei, visible as black, solitary or scattered, globose to subglobose, unilocular, black, with ostioles. ***Ostioles*** papillate, central. ***Clypeus*** brown or sometimes with a halo around the central pore, margin indistinct, consisting of dark, thick-walled hyphae in both epidermal and subepidermal cells. ***Peridium*** 7–11 µm wide (x− = 8.8 µm, n = 15), fused with host tissue, comprising 2–3 layers of pale brown to brown cells of ***textura prismatica***. ***Hamathecium*** 3–6 µm wide (x− = 4 µm, n = 20) µm wide, branched, hyaline, septate, numerous pseudoparaphyses. ***Asci*** 60–75 × 6–10 µm (x− = 65 × 8.5 µm, n = 20), bitunicate, fissitunicate, 6–8-spored, clavate, long-stalked with club-shape, straight. ***Ascospores*** 12–16 × 3–6 µm (x− = 14 × 4.8 µm, n = 30), hyaline to yellowish when immature, brown to red-brown when mature, overlapping uniseriate or biseriate, fusiform to ellipsoid, 1-septate, guttulate, constricted at the septum, upper cell wider and shorter than lower cell and tapering towards ends, sheath drawn out to form polar appendages 4–9 µm long × 1.5–2.5 µm wide (x− = 6 × 1.9 µm, n = 30), from both ends of the ascospores, straight. Anamorph: Not observed.

####### Culture characteristics.

Ascospores germinating on PDA within 24 h, colonies reached 3.5–4 cm in diameter after one month at 25 °C. Colonies on PDA irregular, flat or slightly raised, filamentous, smooth, with undulate margin, from above, hyaline, from below, yellowish at the centre, hyaline at the edge.

####### Materials examined.

China • Yunnan Province, Lincang, on a decaying branch of *Coffeaarabica* (*Rubiaceae*) (24°17'N, 99°99'E, 960 m alt.), 28 July 2022, LiLu, LC1-C5 (HKAS 137611, holotype), isotype MHZU 23-0060, ex-type living culture KUNCC 24-18337 = KUNCC 24-18338, ex-isotype living culture ZHKUCC 23-0630 = ZHKUCC 23-0631.

####### Notes.

**Figure 4.**
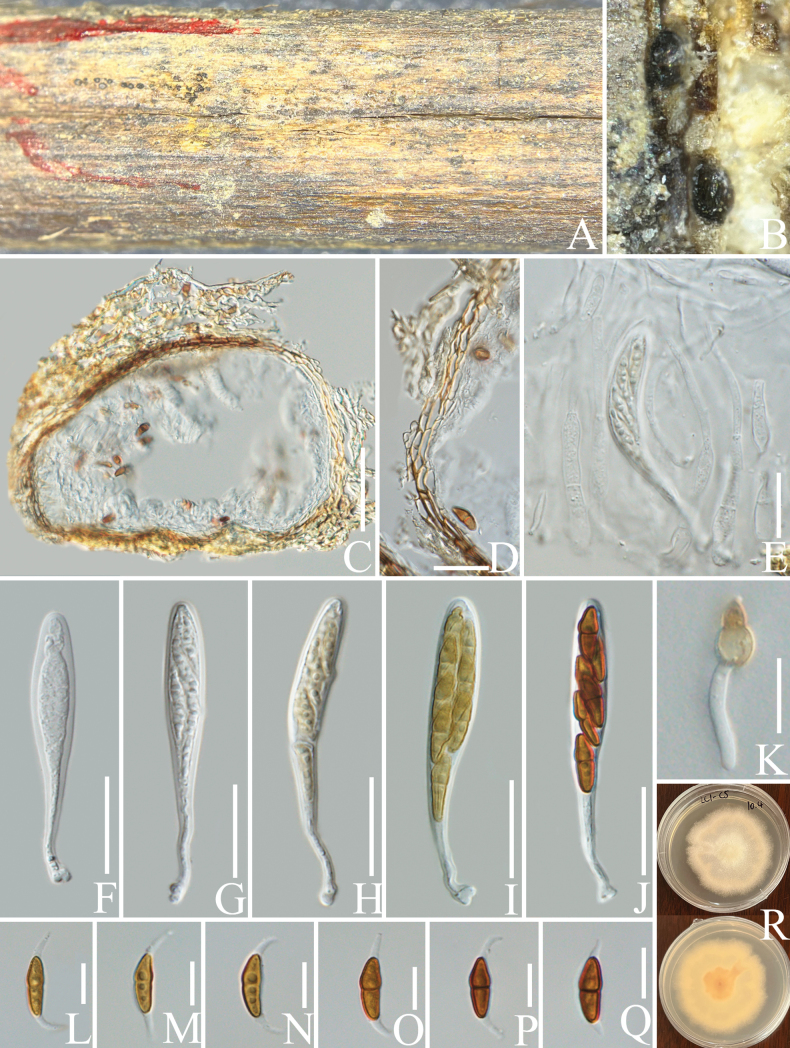
*Montagnulacoffeae* (HKAS 137611, holotype). **A, B** ascomata on a decaying branch of *C.arabica*; **C** longitudinal section of an ascoma; **D** peridium wall; **E** pseudoparaphyses; **F–J** immature and mature asci; **K** germinated ascospore; **L–Q** ascospores; **R** culture on PDA from obverse and reverse. Scale bars: 50 μm (**C**); 20 μm (**D–K**); 10 μm (**L–Q**).

**Figure 5.**
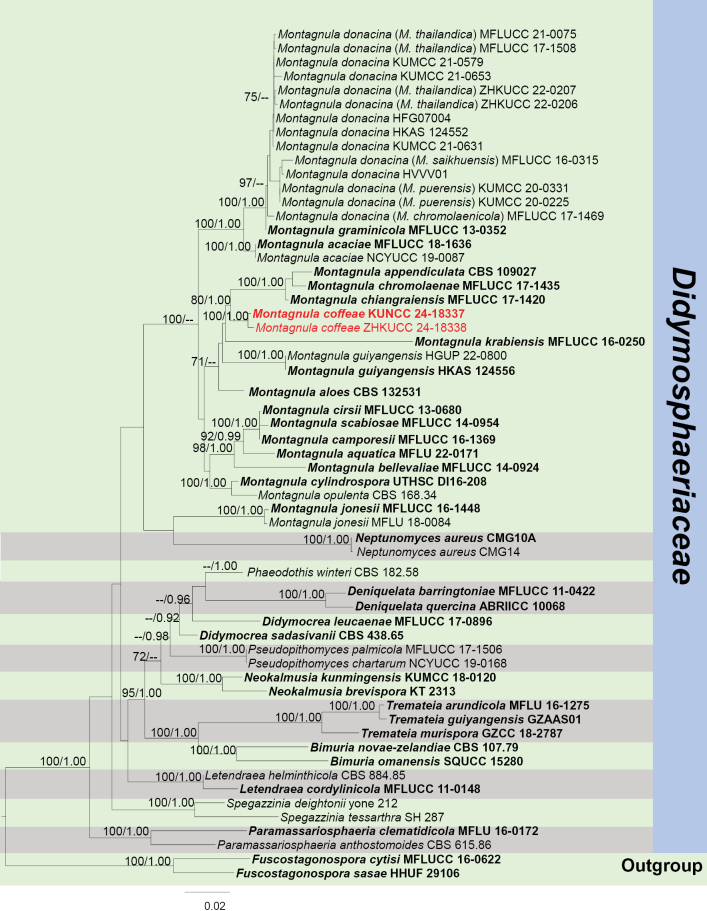
Phylogram generated from the best scoring RAxML tree, based on a combined ITS, LSU, SSU and *TEF*1-α sequence dataset. *Fuscostagonosporacytisi* Jayasiri, Camporesi & K.D. Hyde, (MFLUCC 16-0622) and *F.sasae* Kaz. Tanaka & K. Hiray., (HHUF 29106) are selected as the outgroup taxa. Bootstrap support values for ML equal to or greater than 70% and PP equal to or greater than 0.90 are given above the nodes. All type strains are in bold and newly-generated sequences are in red.

In the concatenated phylogenetic analysis, *Montagnulacoffeae* forms a distinct lineage and is basal to *M.appendiculata*, *M.chromolaenae* and *M.chiangraiensis* (Fig. 5). Based on nucleotide comparisons, *M.coffeae* (ZHKUCC 23-0630) differs from *M.appendiculata* (CBS 109027) by 30/497 bp (6%, without gaps) in ITS and 14/833 bp (1.7%, without gaps) in LSU; differs from *M.chromolaenae* (MFLUCC 17-1435) by 24/497 bp (4.8%, without gaps) in ITS, 12/903 bp (1.3%, without gaps) in LSU and 15/1006 bp (1.5%, without gaps) in SSU; differs from *M.chiangraiensis* (MFLUCC 17-1420) by 20/498 bp (4%, without gaps) in ITS, 13/903 bp (1.4%, without gaps) in LSU and 10/1006 bp (1%, without gaps) in SSU. The morphology of *M.coffeae* is similar to *M.appendiculata* and *M.chromolaenae* in having fusiform, brown and fusiform ascospores with appendages. However, *M.coffeae* has 6–8-spored asci, while *M.appendiculata* has 8-spored asci (Fig. 4; Aptroot (2004)). *Montagnulacoffeae* have guttulate ascospores, while no guttules have been observed on the ascospores of *M.chromolaenae* (Mapook et al. 2020). *Montagnulacoffeae* can be distinguished from *M.chiangraiensis* by the appendages on its ascospores (Mapook et al. 2020). In addition, the PHI test results (Fig. 22b) revealed no significant recombination relationships between *M.coffeae* and its phylogenetically related taxa. The morphological differences and phylogenetic analysis results support the discovery of *M.coffeae* as a new species.

##### *Didymellaceae* Gruyter, Aveskamp & Verkley, Mycological Research 113 (4): 516 (2009).

###### ﻿ Xenodidymella

Taxon classificationFungiPleosporalesDidymellaceae

Qian Chen & L. Cai, Stud. Mycol. 82: 205 (2015).

7E5A5E0D-8E68-5567-8587-EC0BEDEB2D8A

####### Notes.

*Xenodidymella* typified by *X.applanate* (Niessl) Qian Chen & L. Cai, was established by Chen et al. (2015) to accommodate several phoma-like taxa. The new species, *X.coffeae*, is introduced here through phylogenetic and morphological comparisons and isolated from the Arabica coffee plant in China. This is the first *Xenodidymella* species reported from coffee.

###### ﻿ Xenodidymella coffeae

Taxon classificationFungiPleosporalesDidymellaceae

L. Lu & Tibpromma sp. nov.

84877ED1-0F98-503D-88D3-B8FDD7CE48E4

Index Fungorum number: IF901000

Facesofungi number: FoF14726

Fig. 6

####### Etymology.

The species epithet “*coffeae*” refers to the host plant genus “*Coffea*” from which the fungus was isolated.

####### Diagnosis.

Differs from *X.clematidis* Phukhams., Camporesi & K.D. Hyde, by shorter ostioles and cylindrical conidia.

####### Holotype.

HKAS 137610.

####### Description.

***Saprobic*** on decaying branch of *C.arabica*. Teleomorph: Not observed. Anamorph: ***Conidiomata*** 50–80 µm high × 50–90 µm diam. (x− = 62 × 70 µm, n = 20), separate or scattered, semi-immersed, black, globose to subglobose or pyriform, pycnidial, with short ostioles. ***Conidiomatalwall*** 6–12 µm wide (x− = 9 µm, n = 30), composed of 1–3 layers of light brown to brown cells of ***textura angularis***, heavily pigmented at the outer layers, lined with a hyaline layer bearing conidiogenous cells. ***Conidiophores*** inconspicuous or micronematous, often reduced to conidiogenous cells. ***Conidiogenous cells*** lining inner cavity, 4–6 × 4–7 µm (x− = 5.3 × 5.4 µm, n = 30), hyaline, globose to ampulliform, enteroblastic, phialidic. ***Conidia*** 10–15 × 3–4 µm (x− = 12 × 3.3 µm, n = 30), solitary, hyaline, oblong to cylindrical with rounded ends, aseptate, mostly straight, surface smooth or rough.

####### Culture characteristics.

Conidia germinating on PDA within 24 h, colonies reached 6 cm in diameter after two months at 25 °C, filamentous, filiform margin, smooth, flat, with aerial mycelium, from above, brown at the centre, dark brown at the edge, from below, dark brown to black.

####### Materials examined.

China • Yunnan Province, Dali, on a decaying branch of *Coffeaarabica* (*Rubiaceae*) (26°09'N, 101°91'E, 1415 m alt.), 25 July 2022, LiLu, DL-C11 (HKAS 137610, holotype), isotype MHZU 23-0064, ex-type living culture KUNCC 24-18339 = KUNCC 24-18340, ex-isotype living culture ZHKUCC 23-0638 = ZHKUCC 23-0639.

####### Notes.

**Figure 6.**
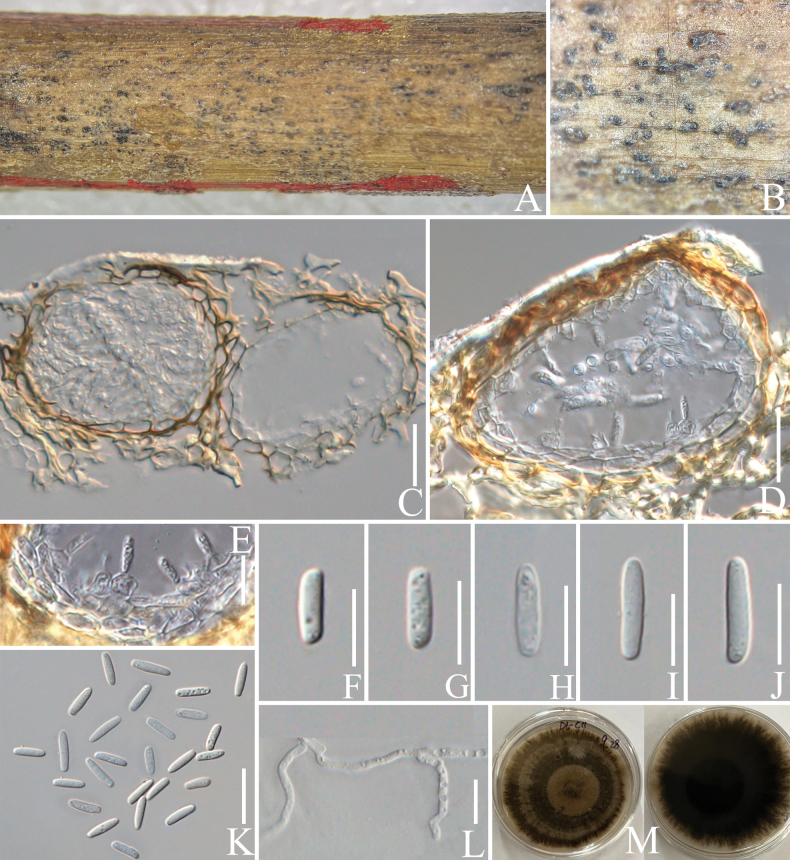
*Xenodidymellacoffeae* (HKAS 137610, holotype). **A, B** conidiomata on a decaying branch of *C.arabica*; **C, D** longitudinal section of a conidioma and conidioma wall; **E** conidiogenous cells with conidia; **F–K** conidia; **L** germinated conidium; **M** culture on PDA from obverse and reverse. Scale bars: 20 μm (**C, D, K, L**); 10 μm (**E–J**).

**Figure 7.**
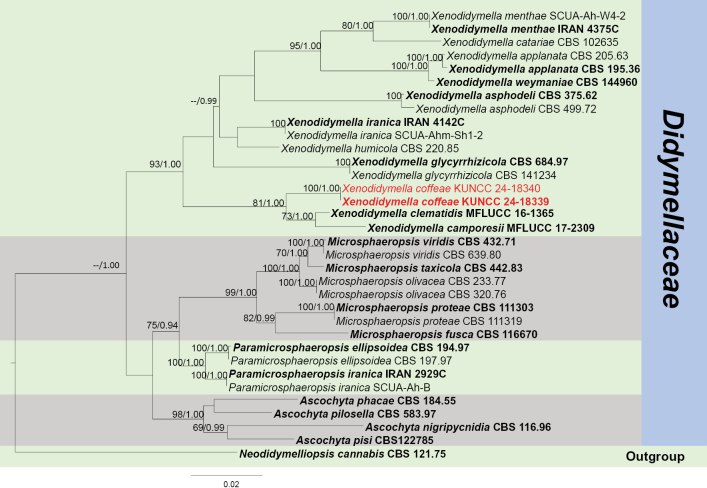
Phylogram generated from the best scoring RAxML tree, based on a combined ITS LSU, *TUB* and *RPB*2 sequence dataset. *Neodidymelliopsiscannabis* (G. Winter) Qian Chen & L. Cai, (CBS 121.75) is selected as the outgroup taxon. Bootstrap support values for ML equal to or greater than 70% and PP equal to or greater than 0.90 are given above the nodes. All type strains are in bold and newly-generated sequences are in red.

In the concatenated phylogenetic analysis, *Xenodidymellacoffeae* shows a close relationship with *X.clematidis* and *X.camporesii* D. Pem, Doilom & K.D. Hyde (Fig. 7). *Xenodidymellacoffeae* conforms to the characteristics of *Xenodidymella* by globose to ampulliform conidiogenous cells and hyaline conidia (Hyde et al. 2020). *Xenodidymellacoffeae* has short ostioles and cylindrical conidia, while *X.clematidis* has long ostioles with oblong-elliptical or oval conidia (Fig. 6; Phukhamsakda et al. (2020)). Besides, the conidia of our species are larger than those of *X.clematidis* (10–15 × 3–4 µm vs. 4–8 × 2–5 μm). *Xenodidymellacamporesii* has only been reported based on its teleomorph (Hyde et al. 2020). Based on nucleotide comparisons, *X.coffeae* (ZHKUCC 23-0638) is different from *X.clematidis* (MFLUCC 16-1365) by 8/482 bp (1.6%, without gaps) of the ITS, 11/786 bp (1.4%, without gaps) of the LSU; and different from *X.camporesii* (MFLUCC 17-2309) by 10/518 bp (2%, with one gap) of the ITS, 16/890 bp (1.8%, without gaps) of the LSU and 26/282 bp (9%, without gaps) of the *TUB*. In addition, the PHI test results (Fig. 22c) revealed no significant recombination relationships between *X.coffeae* and its phylogenetically related taxa. This fungus is, therefore, introduced as a new species of *Xenodidymella*, following the guidelines of Chethana et al. (2021) and Pem et al. (2021).

##### *Lophiostomataceae* Sacc., Syll. Fung. 2: 672 (1883).

###### ﻿ Flabellascoma

Taxon classificationFungiPleosporalesLophiostomataceae

A. Hashim., K. Hiray. & Kaz. Tanaka, Studies in Mycology 90: 167 (2018).

589B3E2B-05CD-55C7-A169-763598CC72B9

####### Notes.

*Flabellascoma* was proposed by Hashimoto et al. (2018) and was typified by *F.minimum* A. Hashim., K. Hiray. & Kaz. Tanaka. *Flabellascoma* species were collected from terrestrial and freshwater habitats with anamorphs and teleomorphs (Hashimoto et al. 2018; Bao et al. 2019; Yu et al. 2022). A new species is added to *Flabellascoma* with a teleomorph from the Arabica coffee plant in Yunnan Province, China and this is the first report of *Flabellascoma* from coffee.

###### ﻿ Flabellascoma coffeae

Taxon classificationFungiPleosporalesLophiostomataceae

L. Lu & Karun. sp. nov.

25C2628D-27BB-53D9-8AED-C97755A97AC6

Index Fungorum number: IF901001

Facesofungi number: FoF14727

Fig. 8

####### Etymology.

The species epithet “*coffeae*” refers to the host plant genus “*Coffea*” from which the fungus was isolated.

####### Diagnosis.

Differs from *F.fusiforme* D.F. Bao, Z.L. Luo, K.D. Hyde & H.Y. Su, by an internal chamber at both ends of ascospores.

####### Holotype.

HKAS 137607.

####### Description.

***Saprobic*** on decaying branch of *C.arabica*. Teleomorph: ***Ascomata*** 280–450 µm high × 200–280 µm diam. (x− = 356 × 230 µm, n = 20) (including neck), solitary, rarely clustered, immersed, visible as black, crest-like ostiolar neck on the substrate, globose to subglobose, uniloculate. ***Ostioles*** central, with a pore-like opening, periphysate. ***Peridium*** 15–30 µm wide (x− = 24 µm, n = 30), composed of several layers of brown, thick-walled cells of ***textura angularis***. ***Hamathecium*** 1.5–3 µm wide (x− = 2.2 µm, n = 30), hyphae-like, septate, branched, pseudoparaphyses. ***Asci*** 60–100 × 10–15 µm (x− = 75 × 12 µm, n = 30), 8-spored, bitunicate, fissitunicate, cylindrical to clavate, straight, with a short furcate sessile, apically rounded with a broad ocular chamber. ***Ascospores*** 20–25 × 5–7 µm (x− = 22 × 6 µm, n = 30), overlapping biseriate, fusiform, hyaline, 1-septate, constricted at the septum, the upper cell slightly wider than the lower cell, guttulate, smooth-walled, with a narrow bipolar sheath. **Sheath** drawn-out at both ends, 4–7 µm long × 2–3 µm wide (x− = 5.6 × 2.5 µm, n = 30), with an internal chamber at both ends of ascospores. Anamorph: Not observed.

####### Culture characteristics.

Ascospores germinating on PDA within 24 h, colonies reached 1.5–2 cm in diameter after twenty days at 25 °C, circular, flat to umbonate, fluffy, smooth, with entire margin, from above, grey, from below, dark grey at the centre, white at the edge.

####### Materials examined.

China, Yunnan Province, Baoshan, on a decaying branch of *Coffeaarabica* (*Rubiaceae*) (24°9'N, 98°8'E, 1050 m alt.), 30 July 2022, LiLu, BS1-C3 (HKAS 137607, holotype), isotype MHZU 23-0063, ex-type living culture KUNCC 24-18341 = KUNCC 24-18342, ex-isotype living culture ZHKUCC 23-0636 = ZHKUCC 23-0637; China, Yunnan Province, Dali, on a decaying branch of *C.arabica*, (26°09'N, 101°91'E, 1416.46 m alt.), 25 July 2022, LiLu, DL-C41 (HKAS 137612, paratype), isoparatype MHZU 23-0062, ex-paratype living culture KUNCC 24-18343 = KUNCC 24-18344, ex-isoparatype living culture ZHKUCC 23-0634 = ZHKUCC 23-0635.

####### Notes.

**Figure 8.**
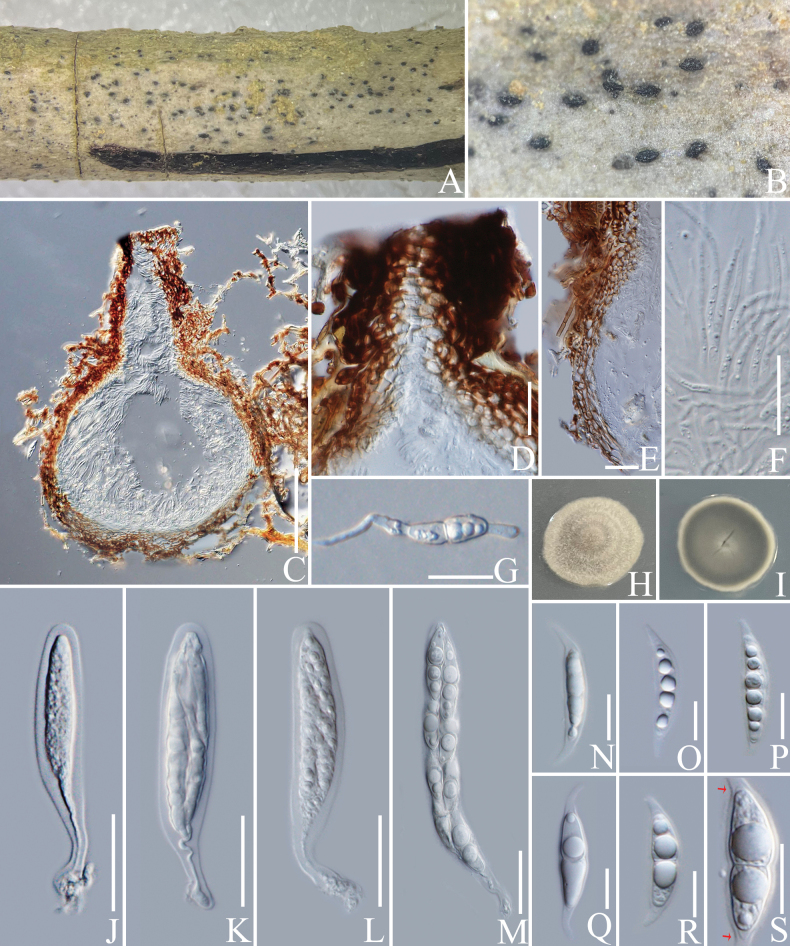
*Flabellascomacoffeae* (HKAS 137607, holotype). **A, B** ascomata on a decaying branch of *C.arabica*; **C** a longitudinal section of an ascoma; **D** ostioles; **E** peridium wall; **F** pseudoparaphyses; **G** germinated ascospore; **H, I** culture on PDA from the obverse and reverse; **J–M** asci; **N–S** ascospores (S: arrowheads indicate an internal chamber in ascospore). Scale bars: 100 μm (**C**); 20 μm (**D–G, J–M**); 10 μm (**N–S**).

**Figure 9.**
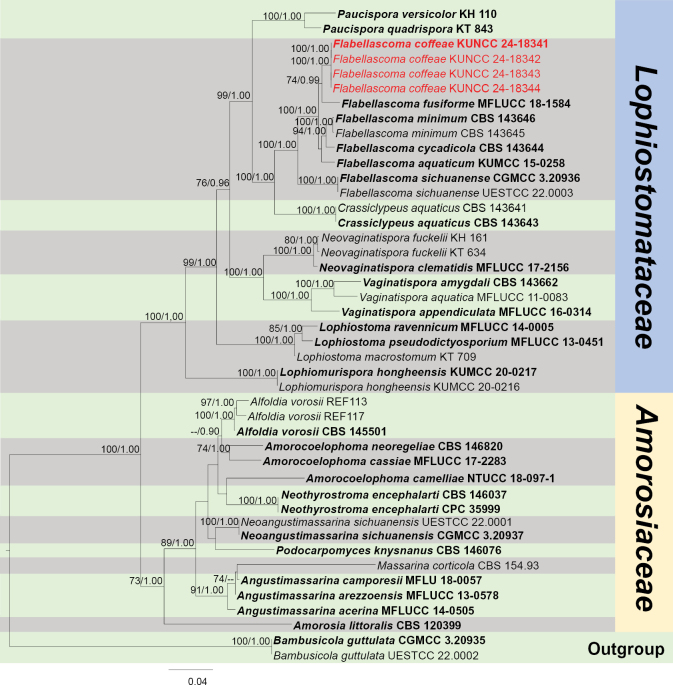
Phylogram generated from the best scoring RAxML tree, based on a combined ITS LSU, SSU, *RPB*2 and *TEF*1-α sequence dataset. *Bambusicolaguttulata* X.D. Yu, S.N. Zhang & Jian K. Liu, (CGMCC 3.20935 and UESTCC 22.0002) are selected as the outgroup taxa. Bootstrap support values for ML equal to or greater than 70% and PP equal to or greater than 0.90 are given above the nodes. All type strains are in bold and newly-generated sequences are in red.

The phylogenetic result, based on SSU, ITS, LSU, *RPB*2 and *TEF*1-α sequence data, showed our new collection *Flabellascomacoffeae* is close to *F.fusiforme* (Fig. 9). *Flabellascomacoffeae* can be distinguished from *F.fusiforme* in having an internal chamber at both ends of ascospores (Fig. 8; Bao et al. (2019)). *Flabellascomacoffeae* also fits well with the morphological characteristics of *Flabellascoma*, such as immersed ascomata with crest-like ostiolar neck, cylindrical-clavate asci and fusiform, hyaline, 1-septate ascospores with a narrow bipolar sheath (Fig. 8; Hashimoto et al. (2018)). Based on nucleotide comparisons, *F.coffeae* (ZHKUCC 23-0636) is different from *F.fusiforme* (MFLUCC 18-1584) by 19/500 bp (3.8%, without gaps) of the ITS, 7/835 bp (0.8%, without gaps) of the LSU and 22/839 bp (2.7%, without gaps) of the *TEF*1-α. In addition, the PHI test results (Fig. 22d) revealed no significant recombination relationships between *F.coffeae* and its phylogenetically related taxa. Therefore, we introduce *F.coffeae* as a new species from coffee in China, based on morphology and multigene phylogeny.

##### *Longiostiolaceae* Phukhams., Doilom & K.D. Hyde, Fungal Diversity 102: 43 (2020).

###### ﻿ Longiostiolum

Taxon classificationFungiPleosporalesLongiostiolaceae

Doilom, Ariyaw. & K.D. Hyde, Fungal Diversity 78: 55 (2016).

7F0AA999-61DC-58BE-A496-44FBCA452713

####### Notes.

*Longiostiolum* was introduced by Li et al. (2016) in *Pleosporales* suborder *Massarineae* M.E. Barr, *incertae sedis*, with *L.tectonae* Doilom, D.J. Bhat & K.D. Hyde, as the type species. Later, Phukhamsakda et al. (2020) introduced the new family *Longiostiolaceae* to accommodate this distinct lineage, based on morphology and phylogenetic analysis. *Longiostiolum* is a monotypic genus with anamorphs and teleomorphs (Li et al. 2016). In this study, *L.coffeae* is introduced with a teleomorph from an Arabica coffee plant in Yunnan, China. This is the first report of *Longiostiolum* from a coffee host.

###### ﻿ Longiostiolum coffeae

Taxon classificationFungiPleosporalesLongiostiolaceae

L. Lu & Tibpromma sp. nov.

E1C61982-6955-5D41-ABE1-427B40234BF7

Index Fungorum number: IF901002

Facesofungi number: FoF14728

Fig. 10

####### Etymology.

The species epithet “*coffeae*” refers to the host plant genus “*Coffea*” from which the fungus was isolated.

####### Diagnosis.

Differs from *L.tectonae* by the presence of sheath in ascospores.

####### Holotype.

HKAS 137602.

####### Description.

***Saprobic*** on decaying branch of *C.arabica*. Teleomorph: ***Ascomata*** 160–280 µm high × 180–280 µm diam. (x− = 212 × 238 µm, n = 15), black spots on the substrate, solitary to scattered, immersed to semi-immersed, when cut horizontally, locules visible as white contents, unilocular, globose to subglobose, with central and short ostioles. ***Peridium*** 20–30 µm thick (x− = 23 µm, n = 15), outer layer consists of 2–4 layers of ***textura angularis***, brown and thick-walled cells, inner layer consists of multi-layers of ***textura angularis***, hyaline and thin-walled cells. ***Hamathecium*** 2–3 µm wide (x− = 2.5 µm, n = 20), numerous, hypha-like, filiform, septate, branched, cellular, pseudoparaphyses, embedded in a gelatinous matrix. ***Asci*** 90–140 × 20–28 µm (x− = 115 × 23 µm, n = 20), bitunicate, 8-spored, cylindrical to clavate, with a short pedicellate, apically rounded, with an ocular chamber. ***Ascospores*** 45–55 × 8–11 µm (x− = 47 × 9 µm, n = 20), overlapping uniseriate to 3-seriate, hyaline, fusoid, often enlarged at the fourth cell, with one transverse septum when young, 7–8 transverse septa when mature, constricted at the centre septa, slightly constricted at other septa, smooth-walled, sheath present. Anamorph: Not observed.

####### Culture characteristics.

Ascospores germinating on PDA within 24 h, colonies reached 4 cm in diameter after one month at 25 °C, circular, with filiform margin, aerial, medium spare, flat or effuse, from above, hyaline, from below, hyaline to light brown at the centre, hyaline at the edge.

####### Materials examined.

China, Yunnan Province, Lincang, on a decaying branch of *Coffeaarabica* (*Rubiaceae*) (22°8'N, 99°4'E, 870 m alt.), 28 July 2022, LiLu, LC3-C3 (HKAS 137602, holotype), isotype MHZU 23-0059, ex-type living culture KUNCC 24-18345 = KUNCC 24-18346, ex-isotype living culture ZHKUCC 23-0628 = ZHKUCC 23-0629.

####### Notes.

**Figure 10.**
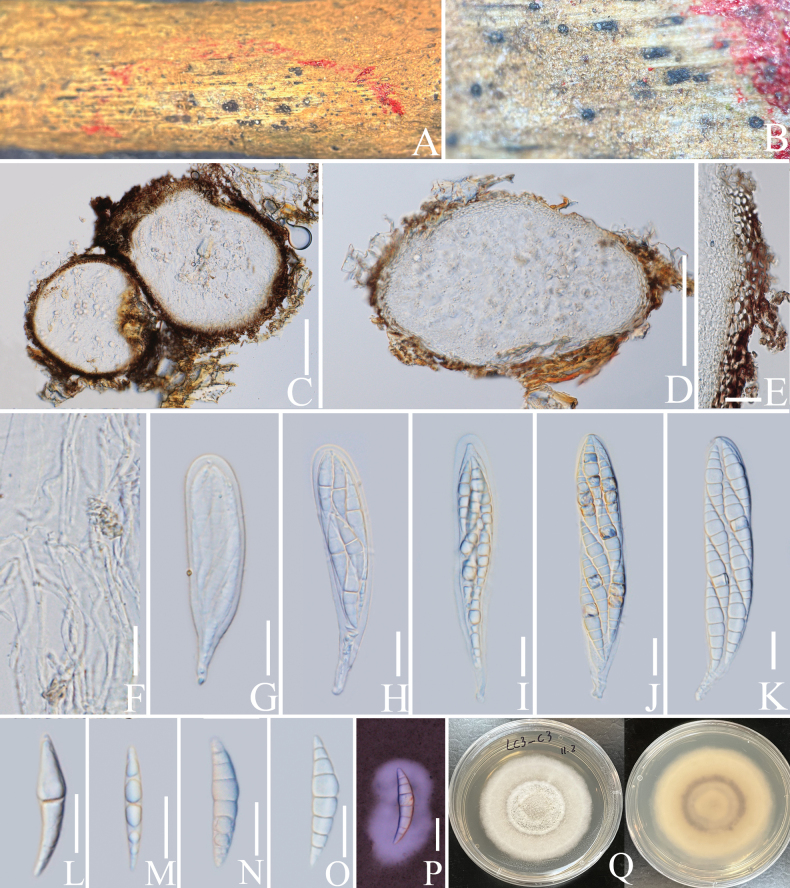
*Longiostiolumcoffeae* (HKAS 137602, holotype). **A, B** ascomata on a decaying branch of *C.arabica*; **C, D** longitudinal section of ascomata; **E** peridium wall; **F** pseudoparaphyses; **G–K** asci; **L–O** ascospores; **P** an ascospore stained with Indian ink; **Q** culture on PDA from obverse and reverse. Scale bars: 100 μm (**C, D**); 20 μm (**E–P**).

**Figure 11.**
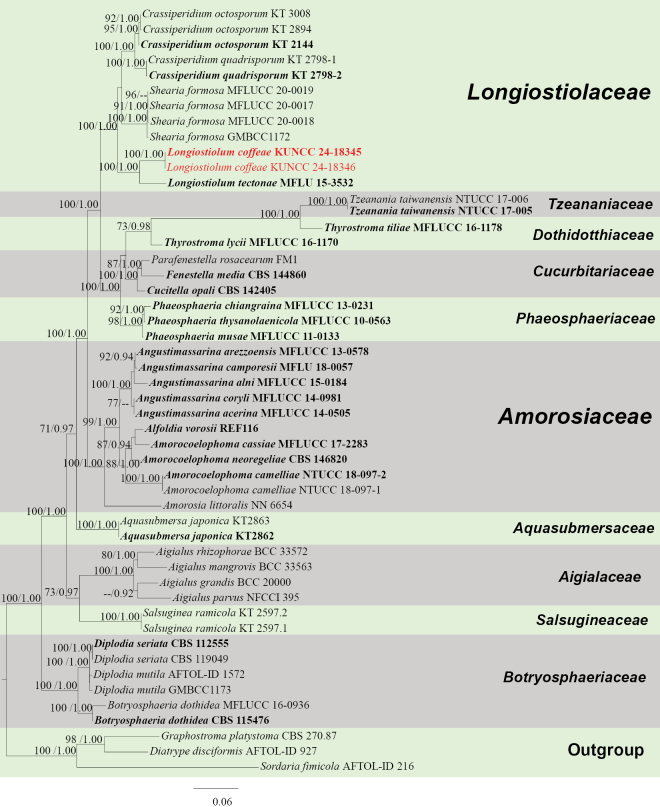
Phylogram generated from the best scoring RAxML tree, based on a combined ITS LSU, SSU, *RPB*2 and *TEF*1-α sequence dataset. *Diatrypedisciformis* (Hoffm.) Fr., (AFTOL-ID 927), *Graphostromaplatystoma* (Schwein.) Piroz. (CBS 270.87) and *Sordariafimicola* (Roberge ex Desm.) Ces. & De Not., (AFTOL-ID 216) are selected as the outgroup taxa. Bootstrap support values for ML equal to or greater than 70% and PP equal to or greater than 0.90 are given above the nodes. All type strains are in bold and newly-generated sequences are in red.

In the concatenated phylogenetic analysis, *Longiostiolumcoffeae* formed a sister branch with *L.tectonae* (MFLU 15-3532) (Fig. 11). Based on nucleotide comparisons, *L.coffeae* (HKAS 137602) is different from *L.tectonae* (MFLU 15-3532) by 70/480 bp (14%, without gaps) of the ITS, 13/863 bp (1.5%, without gaps) of the LSU, 4/665 bp (0.6%, without gaps) of the SSU and 75/920 bp (8%, without gaps) of the *TEF*1-α. Based on morphology, our novel taxon is similar to *L.tectonae* in the hyaline and fusoid ascospores, but differs in the sheath of ascospores, as *L.tectonae* lacks a sheath. Besides, our species has 1–8 transverse septa, while *L.tectonae* has 7–10 transverse septa (Fig. 10; Li et al. (2016); Phukhamsakda et al. (2020)). In addition, the PHI test results (Fig. 22e) revealed no significant recombination relationships between *L.coffeae* and its phylogenetically related taxa. The morphological differences and phylogenetic analyses support the discovery of *L.coffeae* as a new species.

##### *Massarinaceae* Munk, Friesia 5 (3–5): 305 (1956).

###### ﻿ Helminthosporium

Taxon classificationFungiPleosporalesMassarinaceae

Link, Mag. Ges. Naturf. Freunde Berlin 3 (1): 10 (1809).

06AC8DCD-4362-517A-9687-5D16B3BE516C

####### Notes.

*Helminthosporium* was established by Link (1809) and typified by *H.velutinum* Link; it is a polyphyletic genus in *Massarinaceae* of *Pleosporales* (Chen et al. 2022). Members of *Helminthosporium* mainly occur as anamorphs. Little is known about the teleomorph of *Helminthosporium* (Hughes 1953; Subramanian 1987; Tanaka et al. 2015). Herein, we introduce *H.puerensis* as a new species with a teleomorph from the Arabica coffee plant in Yunnan, China.

###### ﻿ Helminthosporium puerensis

Taxon classificationFungiPleosporalesMassarinaceae

L. Lu & Tibpromma sp. nov.

91BBAEBC-14E9-5A8A-8846-E95E252E2BF2

Index Fungorum number: IF901003

Facesofungi number: FoF14729

Fig. 12

####### Etymology.

The epithet refers to the location “Pu’er“ from where the holotype was collected.

####### Diagnosis.

Differs from *H.quercinum* Voglmayr & Jaklitsch and *H.microsorum* D. Sacc., by the hyaline ascospores with inconspicuous sheath.

####### Holotype.

HKAS 137606.

####### Description.

***Saprobic*** on decaying branch of *C.arabica*. Teleomorph: ***Ascomata*** 250–400 × 220–400 µm (x− = 320 × 310 µm, n = 15, including ostioles), solitary to scattered, immersed, visible as black dots with black hair on the host surface, globose to subglobose. ***Ostiolar*** neck central, cylindrical to papillate, surrounded by dark brown clypeus-like structure, without periphyses. ***Peridium*** 20–30 µm wide (x− = 26, n = 20), composed of 4–6 layers of ***textura angularis*** cells, polygonal to rectangular, light brown. ***Hamathecium*** 2–3 µm wide (x− = 2.2, n = 20), hyaline, filiform, branched, septate, pseudoparaphyses numerous. ***Asci*** 80–170 × 15–25 µm (x− = 130 × 19 µm, n = 20), 4–8-spored, fissitunicate, bitunicate, clavate, straight, rounded at the apex, with a narrow apical chamber and faint ring, short-stalked with club-shape, sometimes with long stipes. ***Ascospores*** 25–30 × 7–11 µm (x− = 26.5 × 9 µm, n = 30), mostly straight, 1–3-septate, constricted at the septum, asymmetric, with wider upper cell, hyaline, guttulate, smooth-walled, sheath present. Anamorph: Not observed.

####### Culture characteristics.

Ascospores germinating on PDA within 12 h, colonies reached 4 cm in diameter after two months at 25 °C, surface smooth, circular, flat, with entire margin, from above, hyaline to light yellow, from below, dark brown at the centre, hyaline at the margin.

####### Materials examined.

China, Yunnan Province, Pu’er, on a decaying branch of *Coffeaarabica* (*Rubiaceae*) (22°70'12"N, 101°34'78"E, 900 m alt.), 6 September 2020, LiLu, QX-C7 (HKAS 137606, holotype), isotype MHZU 23-0055, ex-type living culture KUNCC 24-18347 = KUNCC 24-18348, ex-isotype living culture ZHKUCC 23-0620 = ZHKUCC 23-0621.

####### Notes.

**Figure 12.**
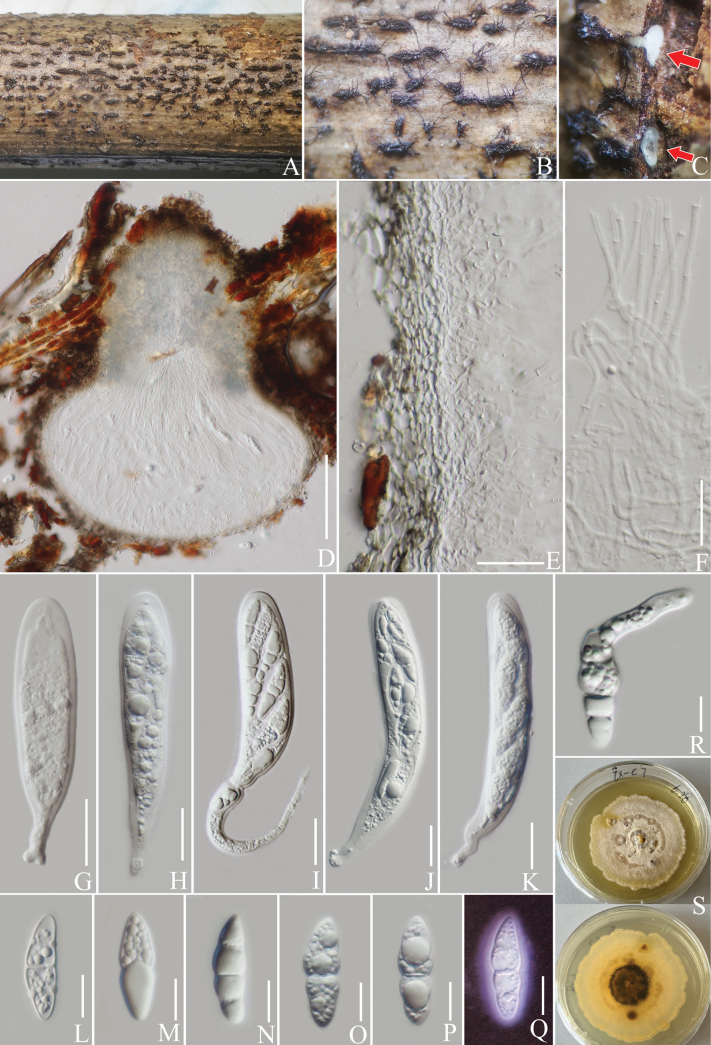
*Helminthosporiumpuerensis* (HKAS 137606, holotype). **A, B** ascomata on a decaying branch of *C.arabica*; **C, D** vertical section of ascomata; **E** peridium wall; **F** pseudoparaphyses; **G–K** asci; **L–P** ascospores; **Q** an ascospore stained with Indian ink; **R** germinated ascospore; **S** culture on PDA from obverse and reverse. Scale bars: 100 μm (**D**); 20 μm (**E–K**); 10 μm (**L–R**).

**Figure 13.**
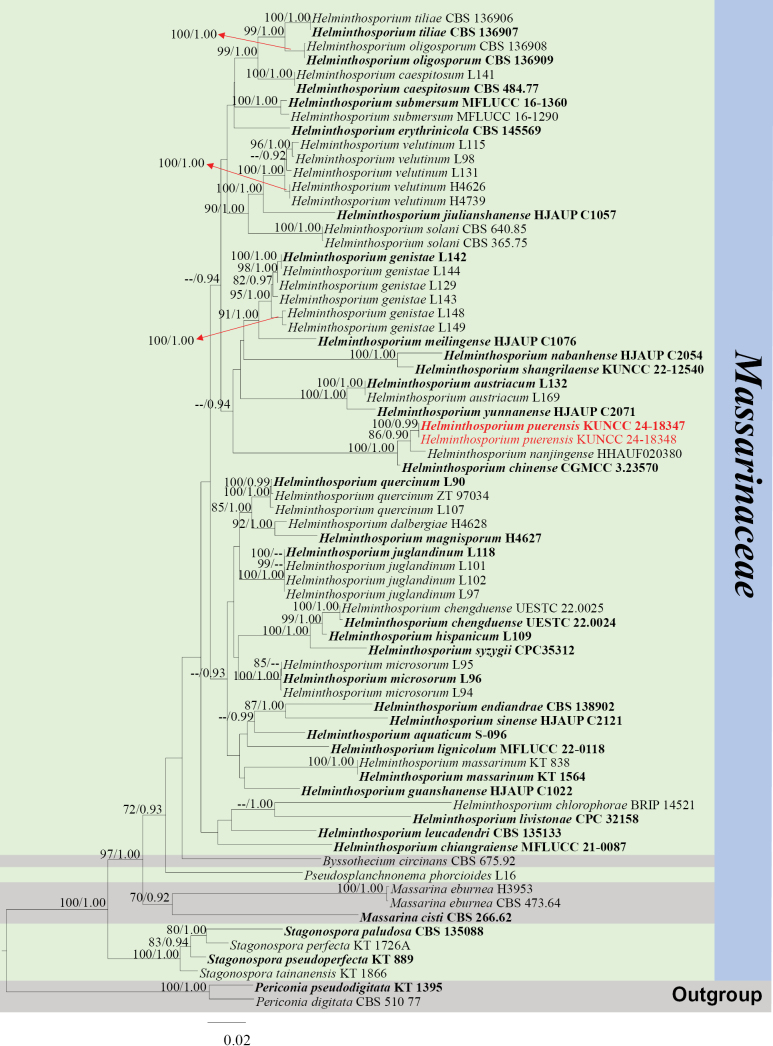
Phylogram generated from the best scoring RAxML tree, based on a combined ITS LSU, SSU, *RPB*2 and *TEF*1-α sequence dataset. *Periconiapseudodigitata* Kaz. Tanaka & K. Hiray., (KT 1395) and *P.digitata* (Cooke) Sacc., (CBS 510.77) are selected as the outgroup taxa. Bootstrap support values for ML equal to or greater than 70% and PP equal to or greater than 0.90 are given above the nodes. All type strains are in bold and newly-generated sequences are in red.

Phylogenetic analyses show that *Helminthosporiumpuerensis* groups with *H.chinense* Y.P. Chen & Maharachch., (CGMCC 3.23570) and *H.nanjingense* Meng Zhang, Xiao J. Wang & H.Y. Wu, (HHAUF 020380) (Fig. 13). *Helminthosporiumchinense* and *H.nanjingense* were only reported as anamorphs from decaying branches of palm trees (Sichuan Province, China) and dead branches of an unidentified tree (Jiangsu Province, China), respectively. Based on nucleotide comparisons, *H.puerensis* (ZHKUCC 23-0620) is different from *H.chinense* (CGMCC 3.23570) by 18/578 bp (3%, without gaps) of the ITS, 3/749 bp (0.4%, without gaps) of the LSU, 16/780 bp (2%, without gaps) of the SSU and 8/320 bp (2.5%, without gaps) of the *TEF*1-α. In comparison, it is different from *H.nanjingense* (HHAUF 020380) in 7/454 bp (1.5%, without gaps) of the ITS (*H.nanjingense* only has ITS sequence data available). *Helminthosporiumchinense* and *H.nanjingense* showed only a small difference in ITS by 7/447 (1.6%, without gaps). Based on BLASTn search results of sequence data, ITS is 97.4% similar to *H.chinense* (ON557754), LSU, SSU and *RPB*2 are closely related to *H.quercinum*, with similarity rates of 97% (KY984338), 98.8% (NG_062196) and 92.5% (KY984398), respectively and *TEF*1-α is 94% (KY984448) similar to *H.microsorum*. In terms of morphological characteristics, our species can be distinguished from *H.quercinum* and *H.microsorum* by the hyaline ascospores with inconspicuous sheath; *H.quercinum* and *H.microsorum* are hyaline to brown ascospores with conspicuous sheath (Voglmayr and Jaklitsch 2017). Our species is most similar to the teleomorph *H.massarinum* in that it has ellipsoidal and hyaline ascospores (Fig. 12; Tanaka et al. (2015)). In addition, the PHI test results (Fig. 22f) revealed no significant recombination relationships between *H.puerensis* and its phylogenetically related taxa. Therefore, the morphological differences and phylogenetic analyses support the introduction of *H.puerensis* as a new species.

##### *Neomassariaceae* H.A. Ariyaw., Jaklitsch & Voglmayr, Cryptogamie, Mycologie 39 (3): 367 (2018).

###### ﻿ Neomassaria

Taxon classificationFungiPleosporalesNeomassariaceae

Mapook, Camporesi & K.D. Hyde, Fungal Diversity 80: 74 (2016).

E76B34EF-DF45-566C-98AA-679167576193

####### Notes.

*Neomassaria* was proposed by Hyde et al. (2016) in *Massariaceae* Nitschke, with *N.fabacearum* Mapook, Camporesi & K.D. Hyde, as the type species. Later, Ariyawansa et al. (2018) collected a neomassaria-like species (*N.formosana* Ariyaw., Jaklitsch & Voglmayr), which formed a sister group with *N.fabacearum* and was well-separated from *Massaria* De Not. species (*Massariaceae*). Therefore, *Neomassaria* species were transferred to the new family *Neomassariaceae* (Ariyawansa et al. 2018; Yang et al. 2022). *Neomassaria* species comprise only teleomorphs (Hyde et al. 2016; Ariyawansa et al. 2018; de Silva et al. 2022; Yang et al. 2022). In this study, we introduce a new *Neomassaria* species with teleomorph, *N.coffeae*, from an Arabica coffee plant in Yunnan Province, China and this is the first report of *Neomassaria* from the coffee host.

###### ﻿ Neomassaria coffeae

Taxon classificationFungiPleosporalesNeomassariaceae

L. Lu & Tibpromma sp. nov.

28F3DF64-E5C4-5242-A224-BB5340C0CA5D

Index Fungorum number: IF901004

Facesofungi number: FoF14730

Fig. 14

####### Etymology.

The species epithet “*coffeae*” refers to the host plant genus “*Coffea*” from which the fungus was isolated.

####### Diagnosis.

Differs from *N.fabacearum* by having guttulate ascospores with mucilaginous sheath and ***textura prismatica*** peridium.

####### Holotype.

HKAS 137608.

####### Description.

***Saprobic*** on a decaying branch of *C.arabica*. Teleomorph: ***Ascomata*** 150–220 µm high × 150–250 µm diam. (x− = 191 × 210 µm, n = 15), solitary to gregarious, semi-immersed to immersed, coriaceous, visible as black dots on the substrate, unilocular, globose or subglobose, ostioles central. ***Peridium*** 10–20 µm wide (x− = 14 µm, n = 15), outer walls comprising 3–4 layers of ***textura prismatica*** cells, brown to dark brown, inner walls thin, hyaline and density. ***Hamathecium*** 1.5–2.5 µm wide (x− = 2 µm, n = 20), hyaline, filiform, septate, branched, cellular, numerous pseudoparaphyses. ***Asci*** 80–110 × 10–15 µm (x− = 93 × 13 µm, n = 20), 8-spored, bitunicate, fissitunicate, oblong to cylindrical, straight, sometimes with short pedicellate, with ocular chamber. ***Ascospores*** 15–18 × 5–7 µm (x− = 16.5 × 5.6 µm, n = 30), uniseriate to biseriate, hyaline, yellowish when mature, ellipsoid to broadly fusiform, 1-septate in the middle, constricted at the septum, guttulate, surrounded by mucilaginous sheath observed clearly when mature. Anamorph: Not observed.

####### Culture characteristics.

Ascospore germinating within 24 h on PDA. Colonies reached 4 cm in diameter after two months at 25 °C. Colonies obverse: circular, flat to slightly raised, fluffy, with filiform margin, white; reverse: brown in centre with yellowish to white edges.

####### Materials examined.

China, Yunnan Province, Baoshan, on a decaying branch of *Coffeaarabica* (*Rubiaceae*) (24°9'N, 98°8'E, 1210 m alt.), 30 July 2022, LiLu, BS2-C19 (HKAS 137608, holotype), isotype MHZU 23-0066, ex-type living culture KUNCC 24-18349 = KUNCC 24-18350, ex-isotype living culture ZHKUCC 23-0642 = ZHKUCC 23-0643.

####### Notes.

**Figure 14.**
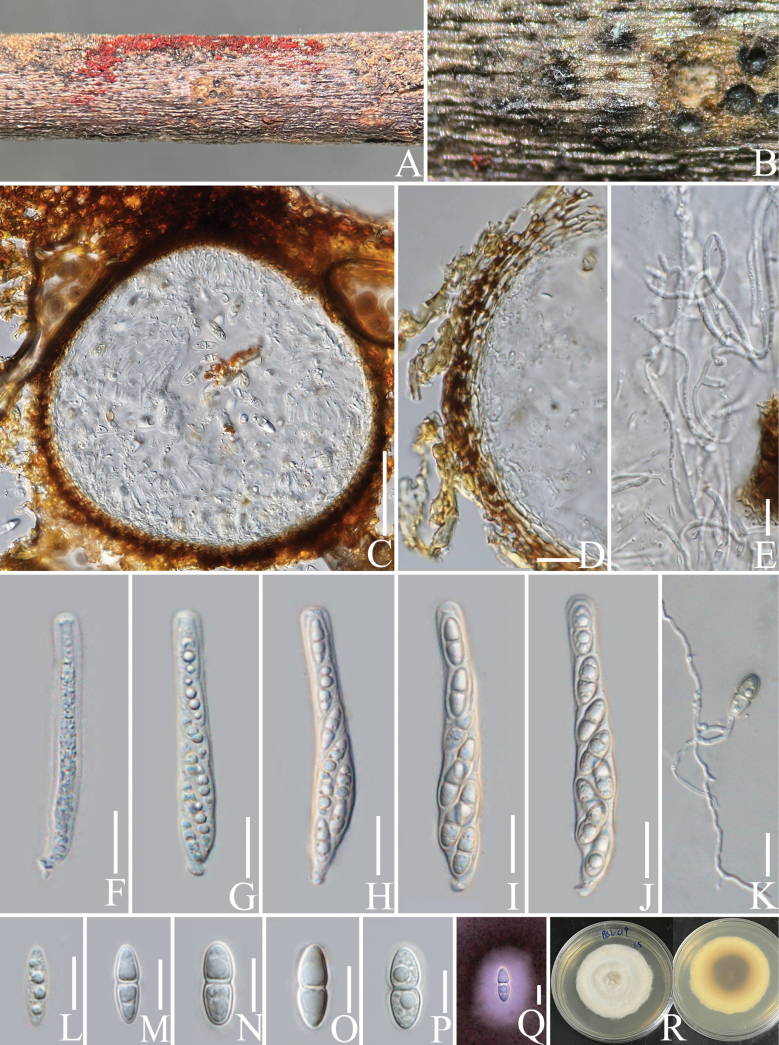
*Neomassariacoffeae* (HKAS 137608, holotype). **A, B** ascomata on a decaying branch of *C.arabica*; **C** longitudinal section of an ascoma; **D** peridium wall; **E** pseudoparaphyses; **F–J** asci; **K** germinated ascospore; **L–P** ascospores; **Q** an ascospore stained with Indian ink; **R** culture on PDA from obverse and reverse. Scale bars: 50 μm (**C**); 10 μm (**D, E, L–Q**); 20 μm (**F–K**).

**Figure 15.**
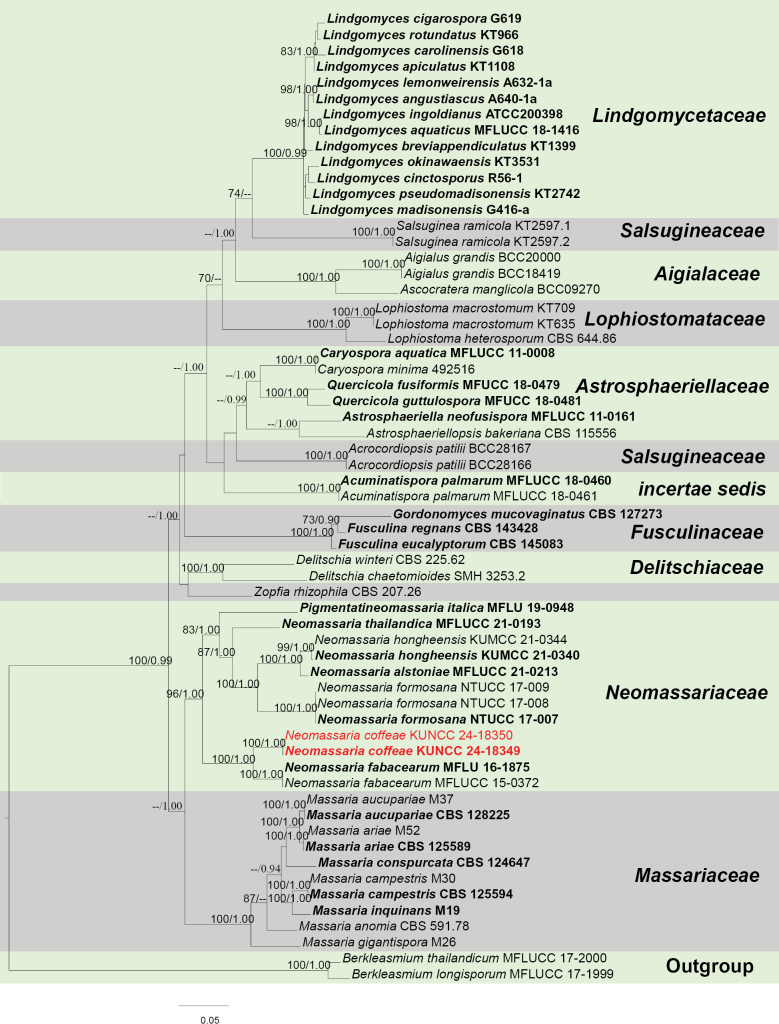
Phylogram generated from the best scoring RAxML tree, based on a combined ITS LSU, SSU, *RPB*2 and *TEF*1-α sequence dataset. *Berkleasmiumlongisporum* Y.Z. Lu, J.C. Kang & K.D. Hyde, (MFLUCC 17-1999) and *B.thailandicum* (Tanney & A.N. Mill.) Y.Z. Lu & K.D. Hyde, (MFLUCC 17-2000) are selected as the outgroup taxa. Bootstrap support values for ML equal to or greater than 70% and PP equal to or greater than 0.90 are given above the nodes. All type strains are in bold and newly-generated sequences are in red.

According to the multi-gene phylogeny, *Neomassariacoffeae* forms a sister lineage to *N.fabacearum* (Fig. 15). Morphologically, *N.coffeae* can be distinguished from *N.fabacearum* by having guttulate ascospores with mucilaginous sheath and ***textura prismatica*** peridium (Fig. 14; Hyde et al. (2016)). Based on nucleotide comparisons, *N.coffeae* (ZHKUCC 23-0642) is different from the type species *N.fabacearum* (MFLU 16-1875) by 18/883 bp (2%, without gaps) of the LSU, 5/868 bp (0.6%, without gaps) of the SSU and 46/843 bp (5.5%, without gaps) of the *TEF*1-α. In addition, the PHI test results (Fig. 22g) revealed no significant recombination relationships between *N.coffeae* and its phylogenetically related taxa. Therefore, based on morphological characteristics and phylogenetic analyses, we introduce our strains as a new species, *N.coffeae*.

##### *Occultibambusaceae* D.Q. Dai & K.D. Hyde, Fungal Diversity 82: 25 (2016).

###### ﻿ Neooccultibambusa

Taxon classificationFungiPleosporalesOccultibambusaceae

Doilom & K.D. Hyde, Fungal Diversity 82: 126 (2016).

A86E1A78-616B-5A07-AE59-DE44A30619A2

####### Notes.

*Neooccultibambusa* was introduced in *Occultibambusaceae*, based on phenotypic characteristics and phylogenetic analyses, with *N.chiangraiensis* Doilom & K.D. Hyde as the type species (Doilom et al. 2017). In this study, a new species of *Neooccultibambusa* is introduced from an Arabica coffee plant in Yunnan Province, China, which is the first report of *Neooccultibambusa* from a coffee host.

###### ﻿ Neooccultibambusa coffeae

Taxon classificationFungiPleosporalesOccultibambusaceae

L. Lu & Tibpromma sp. nov.

F8BBE76F-1564-57E2-B0BB-2D432791B52B

Index Fungorum number: IF901005

Facesofungi number: FoF14731

Fig. 16

####### Etymology.

The species epithet “*coffeae*” refers to the host plant genus “*Coffea*” from which the fungus was isolated.

####### Diagnosis.

Differs from *N.chiangraiensis* and *N.kaiyangensis* X.D. Yu, S.N. Zhang & Jian K. Liu by the greyish-green ascospores.

####### Holotype.

HKAS 137604.

####### Description.

***Saprobic*** on decaying branch of *C.arabica*. Teleomorph: ***Ascomata*** 140–180 × 200–250 µm (x− = 156 × 218 µm, n = 10), superficial to semi-immersed, solitary to gregarious, small, black spots on host surface, unilocular, globose or subglobose, some with ostiolate. ***Peridium*** 10–20 µm wide (x− = 15 µm, n = 20), outer walls comprising 2–4 layers of ***textura angularis*** cells, brown to dark brown, inner walls thin, hyaline and density. ***Hamathecium*** 2–4 µm wide (x− = 3.2 µm, n = 20), hyphae-like, hyaline, filiform, branched, pseudoparaphyses numerous. ***Asci*** 100–200 × 20–30 µm (x− = 156 × 24 µm, n = 20), 8-spored, bitunicate, cylindrical-clavate, straight, with a short furcate, apically rounded, with an ocular chamber. ***Ascospores*** 30–40 × 8–12 µm (x− = 36.6 × 10.3 µm, n = 50), overlapping biseriate, hyaline when young, greyish-green when mature, fusoid or elliptical, 1–3-septate, guttulate, smooth-walled, mucilaginous sheath present. Anamorph: Not observed.

####### Culture characteristics.

Ascospores germinating on PDA within 24 h, colonies reached 4 cm in diameter after two months at 25 °C, mycelia superficial, filamentous, with filiform margin, flat, smooth, from above, brown at the centre, dark brown at the edge, from below, dark brown.

####### Materials examined.

China, Yunnan Province, Xishuangbanna, Pu’wen Town, on a decaying branch of *Coffeaarabica* (*Rubiaceae*) (22°31'18"N, 101°2'44"E, 850 m alt.), 15 September 2021, LiLu, JHPW 13 (HKAS 137604, holotype), isotype MHZU 23-0056, ex-type living culture KUNCC 24-18351, ex-isotype living culture ZHKUCC 23-0622; China, Yunnan Province, Pu’er, on a decaying branch of *C.arabica*, (22°36'2"N, 101°0'59"E, 1015 m alt.), 16 September 2021, LiLu, Pu’er 1-5 (HKAS 137603, paratype) , isoparatype MHZU 23-0057, ex-paratype living culture KUNCC 24-18353, ex-isoparatype living culture ZHKUCC 23-0624.

####### Notes.

**Figure 16.**
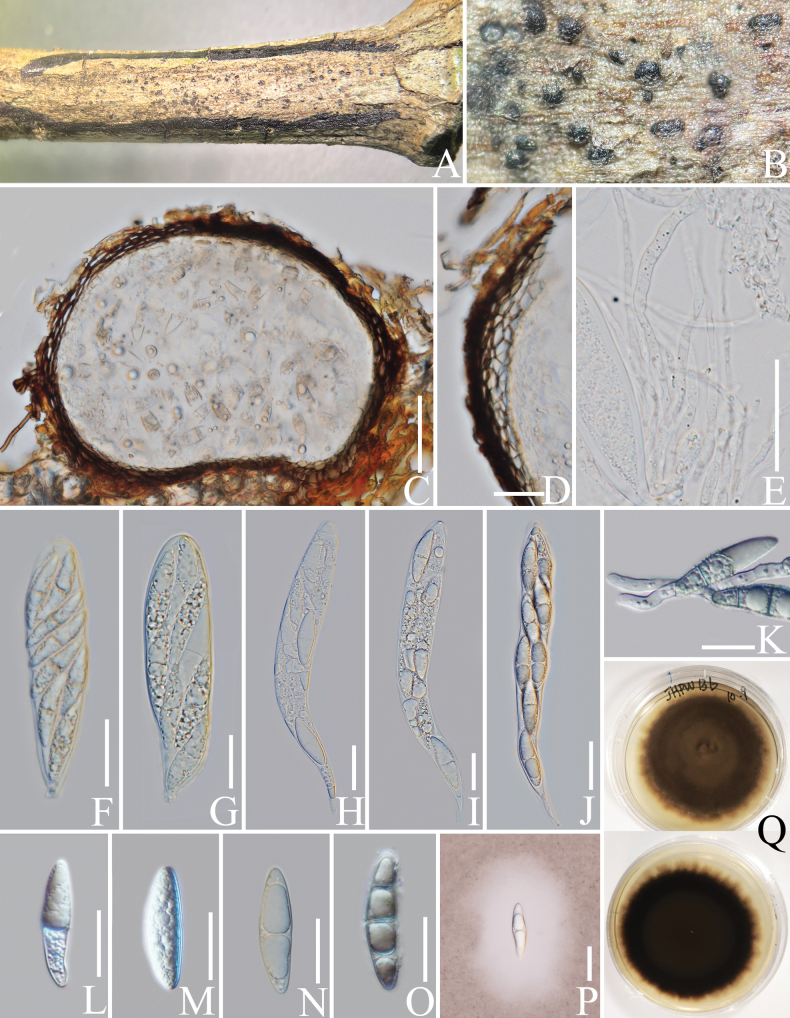
*Neooccultibambusacoffeae* (HKAS 137604, holotype). **A, B** ascomata on a decaying branch of *C.arabica*; **C** longitudinal section of an ascoma; **D** peridium wall; **E** pseudoparaphyses; **F–J** asci; **L–O** ascospores; **P** an ascospore stained with Indian ink; **K** germinated ascospores; **Q** culture on PDA from obverse and reverse. Scale bars: 50 μm (**C**); 20 μm (**D, E, K–P**); 30 μm (**F–J**).

**Figure 17.**
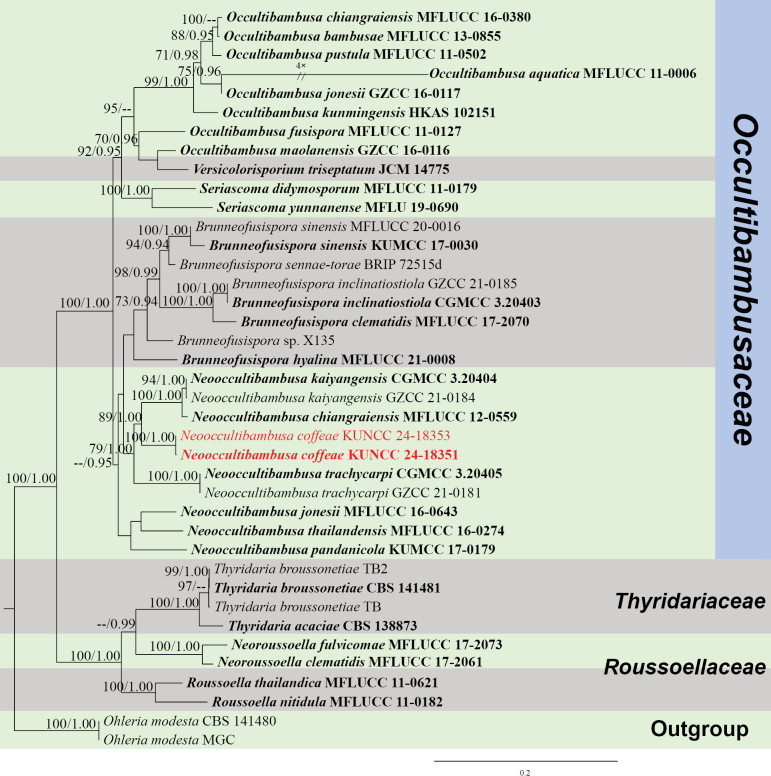
Phylogram generated from the best scoring RAxML tree, based on a combined ITS LSU, SSU, *RPB*2 and *TEF*1-α sequence dataset. *Ohleriamodesta* Fuckel, (CBS 141480 and MGC) are selected as the outgroup taxa. Bootstrap support values for ML equal to or greater than 70% and PP equal to or greater than 0.90 are given above the nodes. All type strains are in bold and newly-generated sequences are in red.

In the concatenated phylogenetic analysis, *Neooccultibambusacoffeae* forms a distinct lineage within *Neooccultibambusa*, closely related to *N.chiangraiensis* and *N.kaiyangensis* (Fig. 17). Morphologically, the new species resembles *N.chiangraiensis* and *N.kaiyangensis* in shape, but differs in the colour of ascospores. The ascospores of *N.coffeae* are greyish-green when mature, while the ascospores of *N.chiangraiensis* and *N.kaiyangensis* are pale brown (Fig. 16; Doilom et al. (2017); Yu et al. (2021)). Based on nucleotide comparisons, *N.coffeae* (ZHKUCC 23-0622) is different from *N.chiangraiensis* (MFLUCC 12-0559) by 50/494 bp (10%, without gaps) of the ITS, 22/872 bp (2.5%, without gaps) of the LSU, 16/943 bp (1.6%, without gaps) of the SSU and 32/613 bp (5%, without gaps) of the *TEF*1-α; In comparison, it is different from *N.kaiyangensis* (CGMCC 3.20404) in 42/467 bp (9%, without gaps) of the ITS, 20/836 bp (2%, without gaps) of the LSU, 16/1009 bp (1.5%, without gaps) of the SSU, 42/963 bp (4%, without gaps) of the *TEF*1-α and 88/886 bp (10%, without gaps) of the *RPB*2. In the BLASTn NCBI GenBank database search of ITS, LSU, SSU, *RPB*2 and *TEF*1-α sequences, ITS results are similar to *Brunneofusisporahyalina* M.S. Calabon & K.D. Hyde, (MFLU 21-0016) with 90% similarity, LSU is similar to *N.trachycarpi* X.D. Yu, S.N. Zhang & Jian K. Liu, (CGMCC 3.20405) with 98% similarity, SSU is similar to *Roussoella* sp. (GMB1323) with 93% similarity, *RPB*2 and *TEF*1-α results are similar to *N.kaiyangensis* (MFLUCC 17-2128) with 90% and 96% similarity, respectively. In addition, the PHI test results (Fig. 22h) revealed no significant recombination relationships between *N.coffeae* and its phylogenetically related taxa. The morphological differences and phylogenetic analyses support the introduction of *N.coffeae* as a new species.

##### *Roussoellaceae* J.K. Liu, Phookamsak, D.Q. Dai & K.D. Hyde, Phytotaxa 181 (1): 7 (2014).

###### ﻿ Pararoussoella

Taxon classificationFungiPleosporalesRoussoellaceae

Wanas., E.B.G. Jones & K.D. Hyde, Fungal Diversity 89: 169 (2018).

FEAB8D68-EA25-5692-A0E7-DD8D43D1BD2A

####### Notes.

*Pararoussoella* was proposed by Wanasinghe et al. (2018) with the type species *P.rosarum* Wanas., E.B.G. Jones & K.D. Hyde. The genus was introduced for the species that are distantly related to the type species of *Roussoella* Sacc., *R.nitidula* Sacc. & Paol. (Phukhamsakda et al. 2020). *Pararoussoella* comprises only five species and is reported as anamorph and teleomorph (Crous et al. 2019, 2020). *Pararoussoellacoffeae* is introduced herein as a new species with anamorph; it was isolated from an Arabica coffee plant in Yunnan Province, China and this is the first report of *Pararoussoella* from a coffee host.

###### ﻿ Pararoussoella coffeae

Taxon classificationFungiPleosporalesRoussoellaceae

L. Lu & Tibpromma sp. nov.

A1C97988-2DA5-5D04-B162-A06E676361ED

Index Fungorum number: IF901006

Facesofungi number: FoF14732

Fig. 18

####### Etymology.

The species epithet “*coffeae*” refers to the host plant genus “*Coffea*” from which the fungus was isolated.

####### Diagnosis.

Differs from other *Pararoussoella* species by the subcylindrical to ellipsoid or, sometimes, ovoid conidia.

####### Holotype.

HKAS 137609.

####### Description.

***Saprobic*** on decaying branch of *C.arabica*. Teleomorph: Not observed. Anamorph: ***Conidiomata*** 60–120 µm high × 100–160 µm diam. (x− = 92 × 121 µm, n = 15), pycnidial, immersed, globose to subglobose, brown, with central ostioles. ***Conidiomatalwall*** 15–25 µm wide (x− = 19 µm, n = 20), hyaline to light brown, thick, 4–6 layers, outer layer composed of brown cells of ***textura angularis***, lined with a hyaline layer bearing conidiogenous cells. ***Conidiophores*** inconspicuous or micronematous, often reduced to conidiogenous cells. ***Conidiogenous cells*** lining the inner cavity, hyaline, smooth, oval to obpyriform or doliiform, phialidic with periclinal thickening at apex, 3–5 × 2–5 µm (x− = 4 × 3.5 µm, n = 30). ***Conidia*** 3.5–5 × 2–3 µm (x− = 4.4 × 2.4 µm, n = 30), aseptate, solitary, guttulate, subcylindrical to ellipsoid or, sometimes, ovoid, smooth, apex bluntly rounded, base truncate, hyaline when young, becoming light brown when mature.

####### Culture characteristics.

Conidia germinating on PDA within 24 h, colonies reached 2.5–3 cm in diameter after one month at 25 °C, filamentous, with entire margin, flat to raised, with many white aerial mycelia, from above, white at the centre, yellowish at the edge, from below, yellowish.

####### Materials examined.

China, Yunnan Province, Lincang, on a decaying branch of *Coffeaarabica* (*Rubiaceae*) (24°17'N, 99°99'E, 960 m alt.), 28 July 2022, LiLu, LC1-C3 (HKAS 137609, holotype), isotype MHZU 23-0061, ex-type living culture KUNCC 24-18355 = KUNCC 24-18356, ex-isotype living culture ZHKUCC 23-0632 = ZHKUCC 23-0633.

####### Notes.

**Figure 18.**
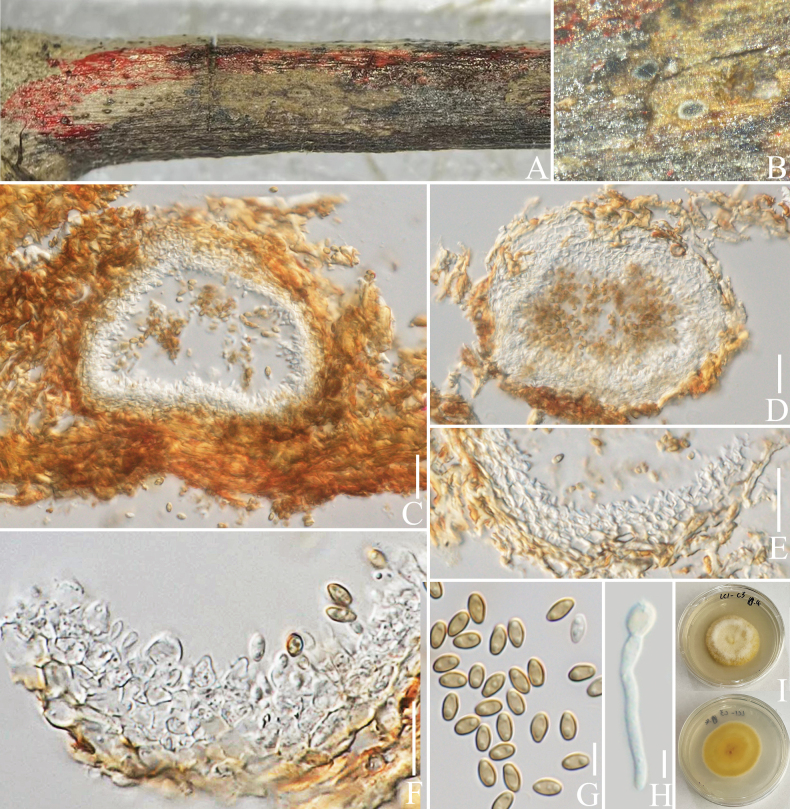
*Pararoussoellacoffeae* (HKAS 137609, holotype). **A, B** appearance of conidiomata on a decaying branch of *C.arabica*; **C, D** longitudinal section of conidiomata; **E** conidiomata wall; **F** conidiogenous cells and conidia; **G** conidia; **H** germinated conidium; **I** culture on PDA from the obverse and reverse. Scale bars: 20 μm (**C–F**); 5 μm (**G, H**).

**Figure 19.**
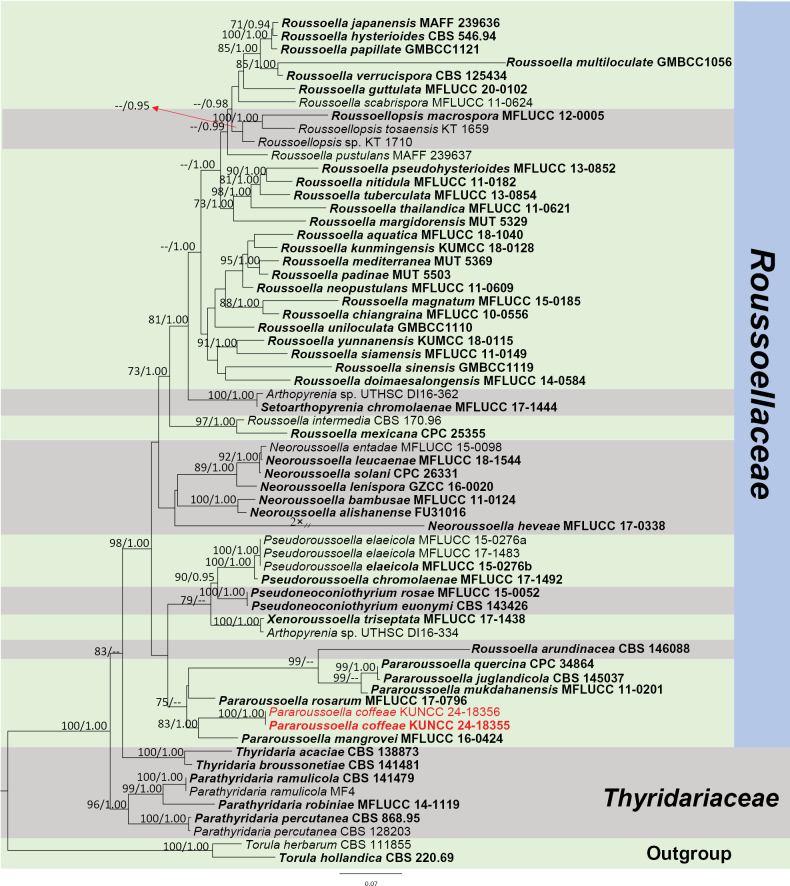
Phylogram generated from the best scoring RAxML tree. based on a combined ITS LSU, SSU, *RPB*2 and *TEF*1-α sequence dataset. *Torulaherbarum* (Pers.) Link, (CBS 111855) and *T.hollandica* Crous, (CBS 220.69) are selected as the outgroup taxa. Bootstrap support values for ML equal to or greater than 70% and PP equal to or greater than 0.90 are given above the nodes. All type strains are in bold and newly-generated sequences are in red.

In the concatenated phylogenetic analysis, *Pararoussoellacoffeae* forms a sister branch basal to *P.mangrovei* (Phukhams. & K.D. Hyde) Phukhams. & K.D. Hyde (Fig. 19). Since *P.mangrovei* has only been reported as teleomorph, we performed nucleotide comparisons; *P.coffeae* (ZHKUCC 23-0632) is different from *P.mangrovei* (MFLUCC 16-0424) by 20/434 bp (4.6%, without gaps) of the ITS, 14/805 bp (1.7%, without gaps) of the LSU, 99/796 bp (12%, without gaps) of the *RPB*2 and 28/718 bp (3.8%, without gaps) of the *TEF*1-α. Based on morphology, our novel taxon in *Pararoussoella* is similar to the species *P.juglandicola* Crous & R.K. Schumach. in aseptate and brown conidia, but differs in the shape of conidia. The conidia of our new species are subcylindrical to ellipsoid, sometimes ovoid, while the conidia of *P.juglandicola* are subcylindrical; besides, the characteristics of guttulate in our species are more distinct than *P.juglandicola* (Fig. 18; Crous et al. (2019)). In addition, the PHI test results (Fig. 22i) revealed no significant recombination relationships between *P.coffeae* and its phylogenetically related taxa. Therefore, the morphological differences and phylogenetic analyses support the introduction of *P.coffeae* as a new species.

##### *Thyridariaceae* Q. Tian & K.D. Hyde, Fungal Diversity 63 (1): 254 (2013).

###### ﻿ Cycasicola

Taxon classificationFungiPleosporalesThyridariaceae

Wanas., E.B.G. Jones & K.D. Hyde, Fungal Diversity 89: 161 (2018).

A315A2EC-065B-5A32-8661-9560D53384EF

####### Notes.

*Cycasicola*, typified by *Cy.goaensis* Wanas., E.B.G. Jones & K.D. Hyde, was introduced by Wanasinghe et al. (2018), based on multi-gene phylogenetic analyses. This genus consists of only two species: *Cy.goaensis* and *Cy.leucaenae* Jayasiri, E.B.G. Jones & K.D. Hyde; both have been reported as saprobic with an anamorph from *Cycas* sp. in India and *Leucaena* sp. in Thailand, respectively (Wanasinghe et al. 2018; Jayasiri et al. 2019). In this study, we provide an updated tree for the family and introduce a new species, *Cy.coffeae*, from an Arabica coffee plant. This is the first report of *Cycasicola* from a coffee host.

###### ﻿ Cycasicola coffeae

Taxon classificationFungiPleosporalesThyridariaceae

L. Lu & Tibpromma sp. nov.

A5329802-AD05-50E6-BFC3-BFD94F135D2F

Index Fungorum number: IF901007

Facesofungi number: FoF14733

Fig. 20

####### Etymology.

The species epithet “*coffeae*” refers to the host plant genus “*Coffea*” from which the fungus was isolated.

####### Diagnosis.

Differs from *Cy.goaensis* by the ellipsoid and larger conidia.

####### Holotype.

HKAS 137613.

####### Description.

***Saprobic*** on decaying branch of *C.arabica*. Teleomorph: Not observed. Anamorph: Coelomycetous. ***Conidiomata*** 100–180 µm high × 120–200 µm diam. (x− = 132 × 160 µm, n = 20), pycnidial, solitary, gregarious or confluent, immersed, unilocular, globose to subglobose or irregular, brown, with central ostiolar. ***Conidiomatalwall*** 15–20 µm wide (x− = 17.5 µm, n = 30), composed of 2–3 brown cells in the outer layers and two hyaline cells in the inner layer, with ***textura angularis*** cells. ***Conidiophores*** inconspicuous or micronematous, often reduced to conidiogenous cells. ***Conidiogenous cells*** 4–6 × 3–5 µm (x− = 5 × 4 µm, n = 30), phialidic, hyaline, cylindrical to ampulliform, smooth-walled. ***Conidia*** 4–7 × 2–3 µm (x− = 5.5 × 2.7 µm, n = 30), hyaline to brownish-orange, ellipsoid to cylindrical or some ovoid, continuous, straight or slightly curved, obtuse at apex and base, aseptate, guttulate, smooth-walled.

####### Culture characteristics.

Conidia germinating on PDA within 24 h, colonies reached 3.5 cm in diameter after one month at 25 °C, circular, radially striated, with a filiform edge, flat, smooth, colonies from above brown at the centre, hyaline to grey at the middle, dark green at the edge, from below, dark green to black.

####### Materials examined.

China, Yunnan Province, Dali, on a decaying branch of *Coffeaarabica* (*Rubiaceae*) (26°09'N, 101°91'E, 1415 m alt.), 25 July 2022, LiLu, DL-C4 (HKAS 137613, holotype), isotype MHZU 23-0065, ex-type living culture KUNCC 24-18357 = KUNCC 24-18358, ex-isotype living culture ZHKUCC 23-0640 = ZHKUCC 23-0641.

####### Notes.

**Figure 20.**
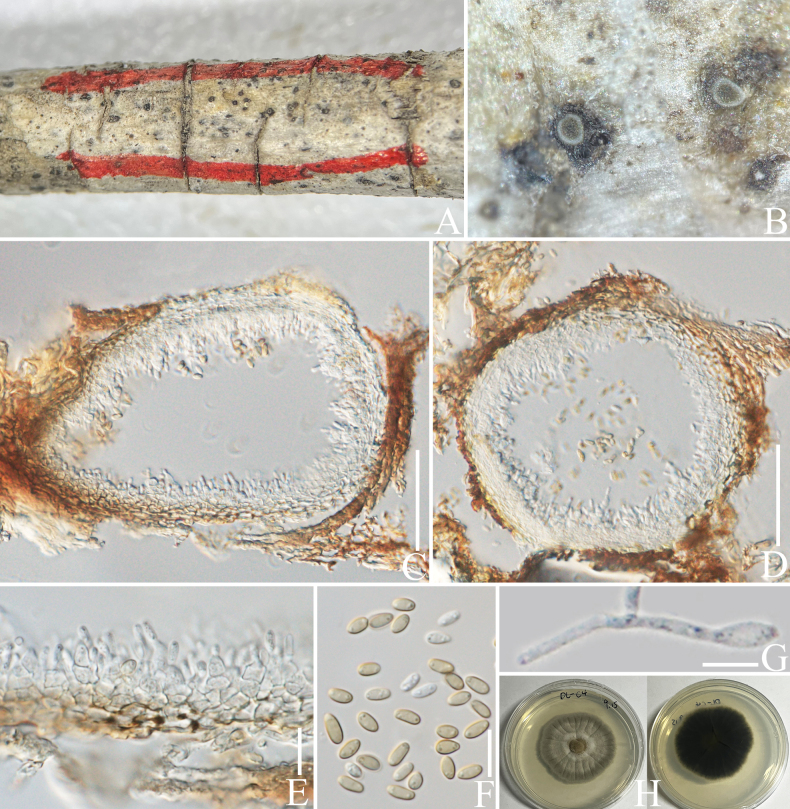
*Cycasicolacoffeae* (HKAS 137613, holotype). **A, B** conidiomata on a decaying branch of *C.arabica*; **C, D** longitudinal section of conidiomata; **E** conidioma wall and conidiogenous cells; **F** conidia; **G** germinated conidium; **H** culture on PDA from the obverse and reverse. Scale bars: 50 μm (**C, D**); 10 μm (**E–G**).

**Figure 21.**
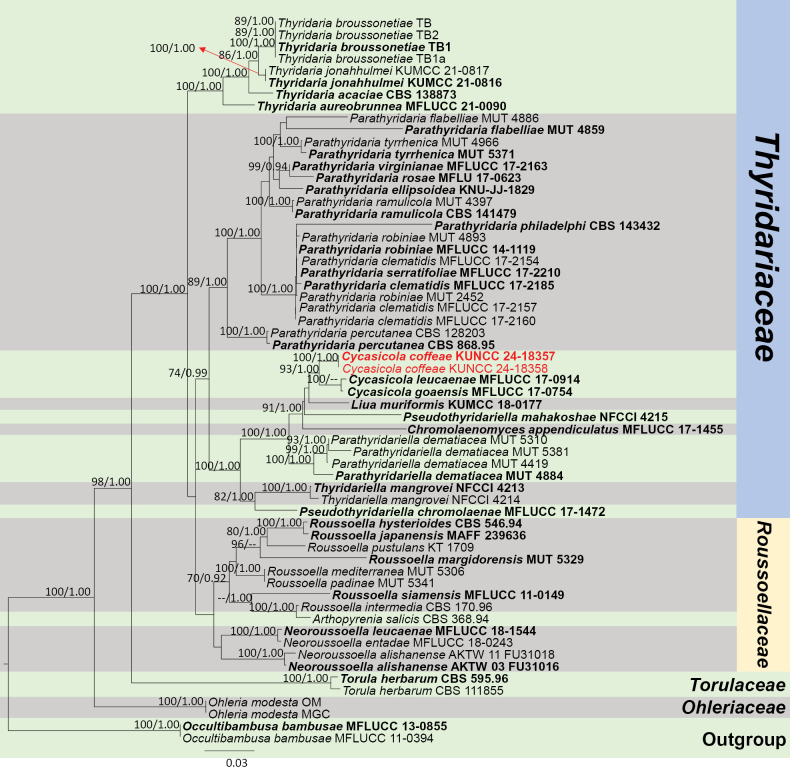
Phylogram generated from the best scoring RAxML tree, based on a combined ITS LSU, SSU and *TEF*1-α sequence dataset. *Occultibambusabambusae* D.Q. Dai & K.D. Hyde, (MFLUCC 13-0855 and MFLUCC 11-0394) are selected as the outgroup taxa. Bootstrap support values for ML equal to or greater than 70% and PP equal to or greater than 0.90 are given above the nodes. All type strains are in bold and newly-generated sequences are in red.

**Figure 22.**
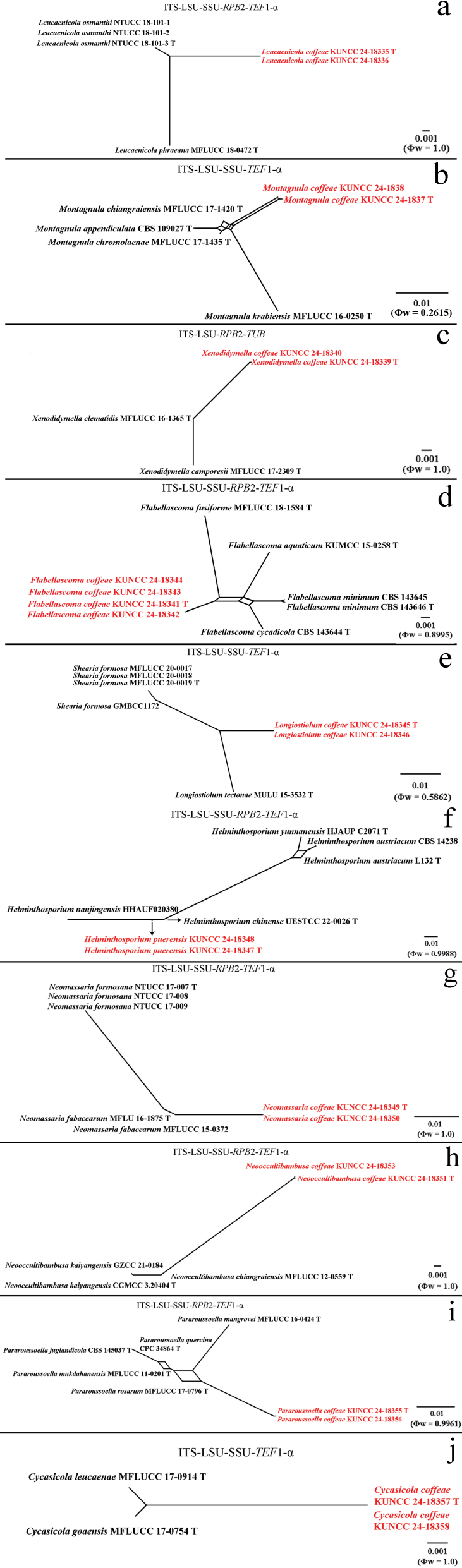
Split graphs showing the results of the pairwise homoplasy index (PHI) test of the new taxa and closely-related taxa using LogDet transformation and splits decomposition. **a***Leucaenicolacoffeae*; **b***Montagnulacoffeae*; **c***Xenodidymellacoffeae*; **d***Flabellascomacoffeae*; **e***Longiostiolumcoffeae*; **f***Helminthosporiumpuerensis*; **g***Neomassariacoffeae*; **h***Neooccultibambusacoffeae*; **i***Pararoussoellacoffeae*; **j***Cycasicolacoffeae*. PHI test result P‐value (Φw) ≤ 0.05 indicates that there is significant recombination between the isolates included in the alignment. The new taxa are in red, “T” indicate the type species and each PHI test value and scale bars are given in the bottom right corner.

In the concatenated phylogenetic analysis, *Cycasicolacoffeae* forms a distinct allied basal lineage with *Cy.goaensis* and *Cy.leucaenae* (Fig. 21). Based on morphology, the conidia of our fungus are very similar to those of *Cy.goaensis* (type species). However, the conidia of *Cy.coffeae* (4–7 × 2–3 µm) are larger than *Cy.goaensis* (3.5–5 × 2.2–2.6 µm) (Fig. 20, Wanasinghe et al. (2018)). Based on nucleotide comparisons, *Cy.coffeae* (ZHKUCC 23-0640) is different from *Cy.goaensis* (MFLUCC 17-0754) by 31/485 bp (6.5%, without gaps) of the ITS, 4/850 bp (0.5%, without gaps) of the LSU, 2/1036 bp (0.2%, without gaps) of the SSU and 25/925 bp (2.7%, without gaps) of the *TEF*1-α. In addition, the PHI test results (Fig. 22j) revealed no significant recombination relationships between *Cy.coffeae* and its phylogenetically related taxa. Therefore, the morphological differences and phylogenetic analyses support the introduction of *Cy.coffeae* as a new species.

## ﻿Discussion

So far, 10,233 new fungal species have been reported in China, mainly distributed in southern China, viz. Yunnan (2136), Taiwan (1470), Guangdong (614), Hainan (532), Sichuan (473), Guizhou (451) and Guangxi (404) provinces (Wang and Cai 2023). Yunnan’s unique geographical location enhances its rich natural resources, with the western region supporting a highland cold-resistant biome and the southern and south-western regions featuring a tropical biome (Shen et al. 2022). This diverse ecological landscape makes Yunnan a region of significant importance for fungal discoveries, contributing 1/5 of the total number of new fungal species identified in China (Wang and Cai 2023). This study introduces ten new species associated with Arabica coffee distributed across ten different genera and families in the order *Pleosporales*, from Yunnan, China. Amongst the ten genera, two genera, viz. *Helminthosporium* and *Montagnula*, have been reported from coffee previously. However, eight genera, viz. *Cycasicola*, *Flabellascoma*, *Leucaenicola*, *Longiostiolum*, *Neomassaria*, *Neooccultibambusa*, *Pararoussoella* and *Xenodidymella* are reported for the first time from Arabica coffee in this study, marking a significant contribution to the field.

In the previous study, *Helminthosporiumcanephorae* Steyaert, *H.coffeae* Massee, *H.glabroides* F. Steven, and *H.ubangiense* Henn. have been reported as pathogens in coffee from Africa (the Democratic Republic of the Congo, Ethiopia and Ghana) and America (Nicaragua) (Lu et al. 2022a). However, most *Helminthosporium* species are usually found as common saprobic fungi from various hosts worldwide; some can be found as pathogens; for example, *H.oryzae* Breda de Haan has been reported as an economically important pathogen that causes brown spot disease in various crops with worldwide occurrences (Alcorn 1983; Voglmayr and Jaklitsch 2017; Boonmee et al. 2021) and only a few have been reported as endophytes with the ability to promote plant growth (Diene et al. 2010). Another genus, *Montagnula* has also been reported from coffee; for example, *Montagnulathailandica* Mapook & K.D. Hyde has been reported as a saprobe in coffee from China (Lu et al. 2022d). Additionally, *Montagnula* species are distributed on various hosts (such as *Arecaceae* Bercht. & J.Presl, *Asparagaceae* Juss., *Cactaceae* Juss., *Fabaceae* Lindl., *Poaceae* Juss. and *Rubiaceae*) and across different habitats (freshwater to terrestrial) worldwide (Wanasinghe et al. 2024). It is important to note that most *Montagnula* species have been found as saprobes with teleomorphs (Sun et al. 2023); however, only *M.cylindrospora* has been isolated from human skin with its anamorph from culture (Crous et al. 2020). The life modes of those fungi are relatively monotonous, with a notable lack of reports on anamorphs occurring on natural substrates. Further investigation into their life modes and complete morphology is essential to understanding their potential impacts on ecosystems.

In addition, the members of the two genera introduced in this study can be found as pathogens and saprobes. For example, three *Leucaenicola* species were reported to be associated with leaf lesions from different hosts from China, but their pathogenicity has not been confirmed (Ariyawansa 2020a, b); another two species have been reported as saprobes from Thailand (Jayasiri et al. 2019). *Xenodidymella* species are notable as pathogens causing cane blight, leaf blight, necrosis, leaf spot or spur blight in different plants and are mainly distributed in Iran and New Zealand (Ahmadpour et al. 2022; Crous et al. 2024; Karimi et al. 2024) and fewer are saprobes and mainly distributed on Germany and Italy (Chen et al. 2015; Hyde et al. 2020). The adaptability of the two genera to diverse niches, with pathogenic members posing significant threats to plant health in certain regions (Lu et al. 2022a), is truly impressive. This highlights the importance of understanding their behaviour and potential impact on plant health. Saprobic counterparts contribute to organic matter decomposition in various ecosystems, which is essential in maintaining nutrient cycling (Karaman et al. 2012). We also hypothesised that *Leucaenicola* and *Xenodidymella* might be able to switch their life mode from pathogens to saprobes, as fungi are capable of switching their modes of nutrition; many pathogenic fungi may persist as saprobes once the plant organ on which they reside, senesces (Hyde et al. 2007).

The rest of the members in *Cycasicola*, *Flabellascoma*, *Longiostiolum*, *Neomassaria*, *Neooccultibambusa* and *Pararoussoella*, all consist entirely as only saprobic species, which have been reported from various hosts. *Cycasicola* species have been reported from India and Thailand (Wanasinghe et al. 2018; Jayasiri et al. 2019), all *Flabellascoma* species have been reported from China (Hashimoto et al. 2018; Bao et al. 2019; Yu et al. 2022), the monotypic genus *Longiostiolum* was described from Thailand (Li et al. 2016), both *Neomassaria* and *Neooccultibambusa* species have been found in China, Thailand and Italy (Hyde et al. 2016; Ariyawansa et al. 2018; de Silva et al. 2022; Yang et al. 2022) and *Pararoussoella* species have been identified from China, Germany, Thailand, Ukraine and the UK (Crous et al. 2019, 2020; Phukhamsakda et al. 2020). These genera have only been reported as saprobic fungi, which play a crucial role in decomposing organic matter across various environments, including coffee plantations, facilitating the renewal and recycling of nutrients and carbon through the action of multiple enzymes, thereby maintaining ecosystem balance (Karaman et al. 2012).

Saprobic fungi have recently garnered significant attention as biocontrol agents and resistance inducers due to their potential spectrum of antifungal activity (de Oliveira et al. 2023). They are capable of secreting enzymes and activating plant defence responses (Yedidia et al. 2003), while also releasing metabolites that inhibit the growth of phytopathogenic communities (de Oliveira et al. 2023). All of the new species described in this study were found to be associated with dead coffee plants, a clear indication of these fungi’s crucial role in the decomposition of organic matter in coffee plantation ecosystems. This study significantly enriches the knowledge of the diversity of coffee-associated saprobic fungi in Yunnan Province of China, paving the way for potential applications such as biocontrol of coffee diseases and discoveries in mycology.

*Pleosporales*, the largest order of *Dothideomycetes*, was proposed by Luttrell (1955) and formally established by Barr (1990). It comprises a quarter of all dothideomycetous species and contains two major suborders: *Massarineae* and *Pleosporineae* M.E. Barr (Zhang et al. 2012). *Pleosporales*, a significant player in the top 50 fungal operational taxonomic units in plants’ diversity of fungal community composition (Yang et al. 2023) and home to about 91 genera and 700 species (Hyde et al. 2024) has been a subject of our intensive study. Our research, conducted rapidly, has unveiled a significant revelation – the order *Pleosporales* is notably predominant in the diversity of saprobic fungi associated with coffee, particularly in the Yunnan Province of China. Over a span of just four years, we have documented 31 new species and records, with 24 of these belonging to *Pleosporales* (See Suppl. material 1: table S1).

Interestingly, this study has expanded the morphological characteristics of *Leucaenicola*. This genus was previously represented by five species known only from their anamorph, including *Leucaenicolaaseptata* and *L.phraeana* isolated from decaying pods of *Leucaena* sp. in Thailand, *L.osmanthi* associated with leaf lesions of *Osmanthusfragrans* in Taiwan (China) and *L.camelliae* and *L.taiwanensis* Ariyaw., I. Tsai & Thambug., isolated from leaf lesions of *Camelliasinensis* (L.) Kuntze, in the same areas as *L.osmanthi*. Herein, *Leucaenicolacoffeae*, supported by combined phylogenetic analysis and morphological evidence from both teleomorph and anamorph stages, is presented as the first teleomorph within the genus (Jayasiri et al. 2019; Ariyawansa et al. 2020a, b). This phenomenon may be attributed to the specific growth conditions in Yunnan, such as the temperature, humidity and nutrient availability, which likely favour the production of the teleomorph. As a result, it offers more comprehensive data for future researchers studying and identifying this genus.

Another fascinating aspect of our research is the adaptability of individual fungal species to different geographical and climatic conditions. For instance, *Neooccultibambusacoffeae* was observed in subtropical (Pu’er) and tropical regions (Xishuangbanna), highlighting its remarkable capacity to overcome geographical and climatic barriers. This adaptability underscores these fungal species’ resilience and potential to colonise diverse ecosystems, offering valuable insights into their ecological roles and exciting potential applications in mycology and environmental science.

## ﻿Conclusion

In conclusion, our research on coffee-associated saprobic fungi across five regions of Yunnan Province (Baoshan, Dali, Lincang, Pu’er and Xishuangbanna), China, revealed a rich diversity and numerous species novelties, with ten new taxa formally described in this study. Compared to other coffee-associated saprobic fungi reported from different regions (1980–2020 vs. 2021–2024), our findings suggest unique fungal community structures that local environmental conditions and agricultural practices may influence. For instance, the high prevalence of saprophytic fungi in our samples may reflect the organic matter decomposition dynamics specific to the study region. These findings, a stepping stone for further research, contribute to understanding coffee-associated fungal ecology and provide a foundation for developing sustainable agricultural practices. This is just the beginning of our journey. We believe that additional potentially new taxonomic taxa, new host records and geographic records are waiting to be discovered in subsequent studies. The taxa with distinctive distributions in this region are yet to be fully explored. Some coffee-associated saprobic fungi may serve as potential biocontrol agents, effectively suppressing the growth of pathogens responsible for coffee plant diseases, while also contributing to the balance of organic matter within coffee ecosystems. However, the full potential of these fungi remains untapped. Urgent and comprehensive research is needed to explore fungi’s diversity, community composition and ecological roles in coffee environments. Research should also investigate the environmental factors influencing their ecological preferences and potential host-specific interactions between pathogenic and saprobic species. Additionally, elucidating the biological functions of coffee-associated saprobic fungi, understanding their host or environmental preferences and studying their interactions with other microorganisms within their habitats are crucial for advancing coffee cultivation and production in China and globally.

## Supplementary Material

XML Treatment for Leucaenicola

XML Treatment for Leucaenicola coffeae

XML Treatment for Montagnula

XML Treatment for Montagnula coffeae

XML Treatment for Xenodidymella

XML Treatment for Xenodidymella coffeae

XML Treatment for Flabellascoma

XML Treatment for Flabellascoma coffeae

XML Treatment for Longiostiolum

XML Treatment for Longiostiolum coffeae

XML Treatment for Helminthosporium

XML Treatment for Helminthosporium puerensis

XML Treatment for Neomassaria

XML Treatment for Neomassaria coffeae

XML Treatment for Neooccultibambusa

XML Treatment for Neooccultibambusa coffeae

XML Treatment for Pararoussoella

XML Treatment for Pararoussoella coffeae

XML Treatment for Cycasicola

XML Treatment for Cycasicola coffeae

## ﻿Additional information

### Adherence to national and international regulations

All the fungal strains used in this study have been legally obtained, respecting the Convention on Biological Diversity (Rio Convention).

### Author ORCIDs

Li Lu https://orcid.org/0000-0003-0977-6414

Samantha C. Karunarathna https://orcid.org/0000-0001-7080-0781

Kunhiraman C. Rajeshkumar https://orcid.org/0000-0003-0401-8294

Abdallah M. Elgorban https://orcid.org/0000-0003-3664-7853

Ruvishika S. Jayawardena https://orcid.org/0000-0001-7702-4885

Sinang Hongsanan https://orcid.org/0000-0003-0550-3152

Nakarin Suwannarach https://orcid.org/0000-0002-2653-1913

Jaturong Kumla https://orcid.org/0000-0002-3673-6541

Yin-Ru Xiong https://orcid.org/0000-0002-4673-606X

Kevin D. Hyde https://orcid.org/0000-0002-2191-0762

Mei-Yan Han https://orcid.org/0009-0004-3705-5408

De-Ge Zheng https://orcid.org/0009-0001-9199-6784

Qiang Li https://orcid.org/0000-0002-9735-8214

Dong-Qin Dai https://orcid.org/0000-0001-8935-8807

Saowaluck Tibpromma https://orcid.org/0000-0002-4706-6547

### Data availability

All tree alignments generated and/or analysed during the current study were deposited to Figshare (https://doi.org/10.6084/m9.figshare.28268366).

## Supplementary materials

Supplementary material 1 Supplementary tables 1–14 https://doi.org/10.3897/imafungus.16.144874.suppl1 Data type docx Explanation note **table S1**: Checklist of coffee-associated saprobic fungi worldwide. **tables S2, S3**: Partial gene regions, primers and amplification procedure. **tables S4–S13**: Names, voucher numbers and corresponding GenBank numbers of the taxa used in the phylogenetic analyses of this study. **table S14**: Phylogenetic statistics in each tree legend. https://binary.pensoft.net/file/1277001This dataset is made available under the Open Database License (http://opendatacommons.org/licenses/odbl/1.0/). The Open Database License (ODbL) is a license agreement intended to allow users to freely share, modify, and use this Dataset while maintaining this same freedom for others, provided that the original source and author(s) are credited.Li Lu, Samantha C. Karunarathna, Kunhiraman C. Rajeshkumar, Abdallah M. Elgorban, Ruvishika S. Jayawardena, Sinang Hongsanan, Nakarin Suwannarach, Jaturong Kumla, Yin-Ru Xiong, Kevin D. Hyde, Mei-Yan Han, De-Ge Zheng, Qiang Li, Dong-Qin Dai, Saowaluck Tibpromma

Supplementary material 2 Tree alignment https://doi.org/10.3897/imafungus.16.144874.suppl2 Data type zip https://binary.pensoft.net/file/1277002This dataset is made available under the Open Database License (http://opendatacommons.org/licenses/odbl/1.0/). The Open Database License (ODbL) is a license agreement intended to allow users to freely share, modify, and use this Dataset while maintaining this same freedom for others, provided that the original source and author(s) are credited.Li Lu, Samantha C. Karunarathna, Kunhiraman C. Rajeshkumar, Abdallah M. Elgorban, Ruvishika S. Jayawardena, Sinang Hongsanan, Nakarin Suwannarach, Jaturong Kumla, Yin-Ru Xiong, Kevin D. Hyde, Mei-Yan Han, De-Ge Zheng, Qiang Li, Dong-Qin Dai, Saowaluck Tibpromma
