# Supplementary material for: ﻿Unveiling fungal diversity associated with coffee trees in China using a polyphasic approach and a global review of coffee saprobic fungi
Source: IMA Fungus. 2025 Mar 10;16:e144874. doi: 10.3897/imafungus.16.144874 (PMC11915015; doi:10.3897/imafungus.16.144874)
Supplement: Supplementary material 1 — Supplementary tables 1–14 [file imafungus-16-e144874-s001.docx]

**Supplementary Table 1**. Checklist of coffee-associated saprobic fungi worldwide.

| Species | Family | Order | Host | Substrate | Country | Reference |
| --- | --- | --- | --- | --- | --- | --- |
| *Akanthomyces johnsonii* | *Cordycipitaceae* | *Hypocreales* | *Coffea* sp. | Decaying leaves | Ohio, The USA | Vincent et al. (1988) |
| *Ascotaiwania coffeae* | *Savoryellaceae* | *Savoryellales* | *Coffea arabica* | Dead and decaying twigs | Yunnan, China | Liu et al. (2024a) |
| *Austropleospora keteleeriae* | *Didymosphaeriaceae* | *Pleosporales* | *Coffea arabica* var. *catimor* | Dead and decaying twigs | Yunnan, China | Lu et al. (2022a) |
| *Brunneofusispora baoshanensis* | *Occultibambusaceae* | *Pleosporales* | *Coffea* sp. | Dead and decaying twigs | Yunnan, China | Liu et al. (2024b) |
| *Calonectria cylindrospora* | *Nectriaceae* | *Hypocreales* | *Coffea arabica* | / | Taiwan, China | Matsushima (1980) |
| *Crassiparies yunnanensis* | *Neohendersoniaceae* | *Pleosporales* | *Coffea arabica* | Dead and decaying twigs | Yunnan, China | Lu et al. (2022b) |
| *Cycasicola coffeae* | *Thyridariaceae* | *Pleosporales* | *Coffea arabica* | Dead and decaying twigs | Yunnan, China | **This study** |
| *Dacryopinax spathularia* | *Dacrymycetaceae* | *Dacrymycetales* | *Coffea arabica* | Plant | Venezuela | Urtiaga (1986) |
| *Deniquelata yunnanensis* | *Didymosphaeriaceae* | *Pleosporales* | *Coffea* sp. | Dead and decaying twigs | Yunnan, China | Lu et al. (2022a) |
| *Dinemasporium coffeanum* | *Chaetosphaeriaceae* | *Chaetosphaeriales* | *Coffea arabica* | Dead twigs | Brazil | Nag Raj (1993) |
| *Fitzroyomyces yunnanensis* | *Stictidaceae* | *Ostropales* | *Coffea* sp. | Dead wood | Yunnan, China | Lu et al. (2021) |
| *Flabellascoma coffeae* | *Lophiostomataceae* | *Pleosporales* | *Coffea arabica* | Dead and decaying twigs | Yunnan, China | **This study** |
| *Hanseiaspora valbyensiskloecker* | *Saccharomycodaceae* | *Saccharomycetales* | *Coffea arabica* | / | Puerto Rico, Virgin Islands (The USA) | Gizaw et al. (2016) |
| *Helminthosporium puerensis* | *Massarinaceae* | *Pleosporales* | *Coffea arabica* | Dead and decaying twigs | Yunnan, China | **This study** |
| *Hyphopichia burtonii* | *Incertae sedis* | *Saccharomycetales* | *Coffea* sp. | Coffee waste | Ethiopia (Sidama and Gedio) | Gizaw et al. (2016) |
| *Kirschsteiniothelia puerensis* | *Kirschsteiniotheliaceae* | *Kirschsteiniotheliales* | *Coffea* sp. | Dead and decaying twigs | Yunnan, China | Hyde et al. (2023) |
| *Leptosphaeria africana* | *Leptosphaeriaceae* | *Pleosporales* | *Coffea robusta* | / | South Africa | Crane and Shearer (1991) |
| *Leptosphaeria centrafricana* | *Leptosphaeriaceae* | *Pleosporales* | *Coffea excelsa* | / | Central African Republic | Crane and Shearer (1991) |
| *Leptosphaeria cylindrospora* | *Leptosphaeriaceae* | *Pleosporales* | *Coffea robusta* | / | Central African Republic | Crane and Shearer (1991) |
| *Leptosphaeria excelsa* | *Leptosphaeriaceae* | *Pleosporales* | *Coffea excelsa* | / | Central African Republic | Crane and Shearer (1991) |
| *Leptosphaeria gigaspora* | *Leptosphaeriaceae* | *Pleosporales* | *Coffea robusta* | / | Central African Republic | Crane and Shearer (1991) |
| *Leptosphaeria lobayensis* | *Leptosphaeriaceae* | *Pleosporales* | *Coffea excelsa* | / | Central African Republic | Crane and Shearer (1991) |
| *Leptosphaeria longispora* | *Leptosphaeriaceae* | *Pleosporales* | *Coffea excelsa* | / | Central African Republic | Crane and Shearer (1991) |
| *Leptosphaeria macrorostra* | *Leptosphaeriaceae* | *Pleosporales* | *Coffea robusta* | / | Central African Republic | Crane and Shearer (1991) |
| *Leptosphaeria oubanguiensis* | *Leptosphaeriaceae* | *Pleosporales* | *Coffea robusta* | / | Central African Republic | Crane and Shearer (1991) |
| *Leptosphaeria tetraspora* | *Leptosphaeriaceae* | *Pleosporales* | *Coffea robusta* | / | Central African Republic | Crane and Shearer (1991) |
| *Leucaenicola coffeae* | *Bambusicolaceae* | *Pleosporales* | *Coffea arabica* | Dead and decaying twigs | Yunnan, China | **This study** |
| *Longiostiolum coffeae* | *Longiostiolaceae* | *Pleosporales* | *Coffea arabica* | Dead and decaying twigs | Yunnan, China | **This study** |
| *Massarioramusculicola chiangraiensis* | *Massariaceae* | *Pleosporales* | *Coffea arabica* | Dead and decaying twigs | Yunnan, China | Hyde et al. (2024) |
| *Montagnula coffeae* | *Didymosphaeriaceae* | *Pleosporales* | *Coffea arabica* | Dead and decaying twigs | Yunnan, China | **This study** |
| *Montagnula thailandica* | *Didymosphaeriaceae* | *Pleosporales* | *Coffea arabica* var. *catimor* | Dead and decaying twigs | Yunnan, China | Lu et al. (2022a) |
| *Neomassaria coffeae* | *Neomassariaceae* | *Pleosporales* | *Coffea arabica* | Dead and decaying twigs | Yunnan, China | **This study** |
| *Neooccultibambusa coffeae* | *Occultibambusaceae* | *Pleosporales* | *Coffea arabica* | Dead and decaying twigs | Yunnan, China | **This study** |
| *Nigrograna asexualis* | *Nigrogranaceae* | *Pleosporales* | *Coffea arabica* | Dead and decaying twigs | Yunnan, China | Lu et al. (2022c) |
| *Nigrograna coffeae* | *Nigrogranaceae* | *Pleosporales* | *Coffea arabica* | Dead and decaying twigs | Yunnan, China | Lu et al. (2022c) |
| *Nigrograna puerensis* | *Nigrogranaceae* | *Pleosporales* | *Coffea arabica* | Dead and decaying twigs | Yunnan, China | Lu et al. (2022c) |
| *Ostropomyces pruinosella* | *Stictidaceae* | *Ostropales* | *Coffea* sp. | Dead wood | Yunnan, China | Lu et al. (2021) |
| *Paraconiothyrium yunnanensis* | *Didymosphaeriaceae* | *Pleosporales* | *Coffea* sp. | Dead and decaying twigs | Yunnan, China | Lu et al. (2022a) |
| *Pararoussoella coffeae* | *Roussoellaceae* | *Pleosporales* | *Coffea arabica* | Dead and decaying twigs | Yunnan, China | **This study** |
| *Phaeodothis winteri* | *Didymosphaeriaceae* | *Pleosporales* | *Coffea arabica*, *C. robusta* | Leaves | Brazil, India, Malaysia | Aptroot (1995) |
| *Phaeosaccardinula coffeicola* | *Chaetothyriaceae* | *Chaetothyriales* | *Coffea arabica* | Leaves | Chiang Mai, Thailand | Maharachchikumbura et al. (2018) |
| *Phialocephala mexicana* | *Mollisiaceae* | *Helotiales* | *Coffea arabica* | Dead leaves | Cote d'Ivoire | Onofri and Zucconi (1984) |
| *Phomatospora uniseriata* | *Phomatosporaceae* | *Phomatosporales* | *Coffea* sp. | Dead and decaying twigs | Yunnan, China | Hyde et al. (2024) |
| *Phragmocapnias asiaticus* | *Trichomeriaceae* | *Chaetothyriales* | *Coffea arabica* | / | Thailand | Chomnunti et al. (2011) |
| *Physcia* sp. | *Physciaceae* | *Caliciales* | *Coffea arabica* | Plant | Venezuela | Urtiaga (1986) |
| *Phytophthora* sp. | *Peronosporaceae* | *Peronosporales* | *Coffea arabica* | / | Panama | Piepenbring (2006) |
| *Pichiaamenthionina* var. *menthionina* | *Saccharomycetaceae* | *Saccharomycetales* | *Coffea* sp., coffee waste | Leaves | Brazil, India, Malaysia | Gizaw et al. (2016) |
| *Pseudocercospora coffeigena* | *Mycosphaerellaceae* | *Mycosphaerellales* | *Coffea arabica* | Plant | Yunnan, China | Guo (1999) |
| *Pseudocoleophoma puerensis* | *Dictyosporiaceae* | *Pleosporales* | *Coffea arabica var. catimor* | Dead and decaying twigs | Yunnan, China | Lu et al. (2022a) |
| *Pseudocoleophoma yunnanensis* | *Dictyosporiaceae* | *Pleosporales* | *Coffea* sp. | Dead and decaying twigs | Yunnan, China | Lu et al. (2022a) |
| *Pseudoconiocessia xishuangbannaensis* | *Coniocessiaceae* | *Xylariales* | *Coffea liberica* | Dead and decaying twigs | Yunnan, China | Lu et al. (2024) |
| *Rhodotorula aurantiaca* | *Sporidiobolaceae* | *Sporidiobolales* | *Coffea* sp. | Coffee waste | Ethiopia (Sidama and Gedio) | Gizaw et al. (2016) |
| *Rhodotorula hylophila* | *Sporidiobolaceae* | *Sporidiobolales* | *Coffea* sp. | Coffee waste | Ethiopia (Sidama and Gedio) | Gizaw et al. (2016) |
| *Rhytidhysteron coffeae* | *Hysteriaceae* | *Hysteriales* | *Coffea* sp. | Dead and decaying twigs | Yunnan, China | Du et al. (2023) |
| *Stilbohypoxylon coffeicola* | *Xylariaceae* | *Xylariales* | *Coffea arabica* | / | Guadeloupe | Petrini (2004) |
| *Stilbohypoxylon quisquiliarum* | *Xylariaceae* | *Xylariales* | *Coffea robusta* | / | Central Africa | Petrini (2004) |
| *Subramaniomyces fusisaprophyticus* | *Xylariaceae* | *Xylariales* | *Coffea* sp. | Dead, fallen leaves | Ethiopia | Bhat and Sutton (1985) |
| *Torula mackenziei* | *Torulaceae* | *Pleosporales* | *Coffea* sp. | Dead and decaying twigs | Yunnan, China | Hyde et al. (2024) |
| *Trichomerium chiangmaiensis* | *Trichomeriaceae* | *Chaetothyriales* | *Coffea arabica* | Leaves | Chiang Mai, Thailand | Maharachchikumbura et al. (2018) |
| *Trinacrium indica* | *Orbiliaceae* | *Orbiliales* | *Coffea arabica* | Decaying leaves | India | Bhat (2008) |
| *Xenocamarosporium acaciae* | *Didymosphaeriaceae* | *Pleosporales* | *Coffea* sp. | Dead and decaying twigs | Yunnan, China | Lu et al. (2022a) |
| *Xenodidymella coffeae* | *Didymellaceae* | *Pleosporales* | *Coffea arabica* | Dead and decaying twigs | Yunnan, China | **This study** |

“/” indicates the substrate unavailability.

**Supplementary Table 2** Partial gene regions and primers used in this study

| Gene region/loci | PCR primers (forward/reverse) | References |
| --- | --- | --- |
| ITS | ITS5/ITS4 | White et al. (1990) |
| LSU | LR0R/LR5 | Vilgalys and Hester (1990) |
| *RPB*2 | fRPB2-5F/fRPB2-7cR | Liu et al. (1999) |
| SSU | NS1/NS4 | White et al. (1990) |
| *TEF*1-α | 983F/2218R | Rehner and Buckley (2005) |
| *TUB* | Bt2a/Bt2b | Sheir-Neiss et al. (1978) |

**Supplementary Table 3** Partial gene regions and amplification procedure used in this study

| Step | ITS/LSU/SSU/*TEF*1-α/*TUB* | | | *RPB*2 | | |
| --- | --- | --- | --- | --- | --- | --- |
|  | Temperature | Time | Cycle | Temperature | Time | Cycle |
| Initial denaturation | 94 ℃ | 3 min | - | 95 ℃ | 5 min | - |
| Denaturation | 94 ℃ | 45 s | 35 | 95 ℃ | 1 min | 40 |
| Annealing | 55 ℃ | 50 s |  | 56 ℃ | 2 min |  |
| Extension | 72 ℃ | 1 min |  | 72 ℃ | 90 s |  |
| Final extension | 72 ℃ | 10 min | - | 72 ℃ | 10 min | - |

**Supplementary Table 4**. Names, voucher numbers, and corresponding GenBank numbers of the taxa used in the phylogenetic analyses of *Leucaenicola*.

| Species | | Voucher number | GenBank accession number | | | | | References |
| --- | --- | --- | --- | --- | --- | --- | --- | --- |
|  |  |  | LSU | SSU | ITS | *RPB*2 | *TEF*1-α |  |
| *Bambusicola bambusae* | MFLUCC 11-0614 | | JX442035 | JX442039 | NR_121546 | KP761718 | KP761722 | Dai et al. (2012) |
| *Bambusicola didymospora* | MFLUCC 10-0557 | | KU863105 | KU872111 | KU940117 | KU940164 | KU940188 | Dai et al. (2016) |
| *Bambusicola fusispora* | MFLUCC 20-0149 | | MW076531 | MW076529 | MW076532 | MW034589 | – | Monkai et al. (2021) |
| *Bambusicola irregulispora* | MFLUCC 11-0437 | | JX442036 | JX442040 | NR_121547 | KP761719 | KP761723 | Dai et al. (2012) |
| *Bambusicola loculata* | MFLUCC 13-0856 | | KP761729 | KP761735 | KP761732 | KP761715 | KP761724 | Dai et al. (2015) |
| *Bambusicola massarinia* | MFLUCC 11-0389 | | KU863111 | KU872115 | NR_121548 | KU940169 | KU940192 | Dai et al. (2012) |
| *Bambusicola splendida* | MFLUCC 11-0439 | | KU863110 | JX442042 | NR_121549 | KU940168 | KP761726 | Dai et al. (2012) |
| *Bambusicola thailandica* | MFLUCC 11-0147 | | KU863108 | KU872113 | KU940119 | KU940166 | KU940191 | Dai et al. (2016) |
| *Bambusicola triseptatispora* | MFLUCC 11-0166 | | KU863109 | – | NR_153624 | KU940167 | – | Dai et al. (2016) |
| *Corylicola italica* | MFLU 19-0500 | | MT554926 | MT554923 | MT554925 | MT590776 | – | Wijesinghe et al. (2020a) |
| *Corylicola italica* | MFLUCC 20-0111 | | MT626713 | MT633084 | MT633085 | MT635596 | MT590777 | Wijesinghe et al. (2020a) |
| *Latorua caligans* | CBS 576.65 | | KR873266 | – | KR873232 | – | – | Crous et al. (2015) |
| *Latorua grootfonteinensis* | CBS 369.72 | | KR873267 | – | – | – | – | Crous et al. (2015) |
| *Leucaenicola aseptata* | MFLUCC 17-2423 | | NG_066309 | NG_065776 | NR_163332 | MK434891 | MK360059 | Jayasiri et al. (2019) |
| *Leucaenicola camelliae* | NTUCC 18-093-1 | | MT071273 | MT071224 | MT112297 | MT743280 | MT249023 | Ariyawansa et al. (2020a) |
| *Leucaenicola camelliae* | NTUCC 18-093-2 | | MT071274 | MT071225 | MT112298 | MT743281 | MT249024 | Ariyawansa et al. (2020a) |
| *Leucaenicola camelliae* | NTUCC 18-093-3 | | MT071276 | MT071227 | MT112300 | MT743282 | MT346593 | Ariyawansa et al. (2020a) |
| *Leucaenicola camelliae* | NTUCC 18-093-4 | | MT071278 | MT071229 | MT112302 | MT743283 | MT374091 | Ariyawansa et al. (2020a) |
| ***Leucaenicola coffeae*** | **KUNCC24-18335** | | **OR428417** | **OR428375** | **OR428395** | **OR515519** | **OR509749** | **This study** |
| ***Leucaenicola coffeae*** | **KUNCC24-18336** | | **OR428418** | **OR428376** | **OR428396** | **OR515520** | **OR509750** | **This study** |
| *Leucaenicola osmanthi* | NTUCC 18-101-3 | | MN908610 | MN908607 | MN908564 | MN915019 | MN918598 | Ariyawansa et al. (2020b) |
| *Leucaenicola osmanthi* | NTUCC 18-101-2 | | MN908611 | MN908608 | MN908566 | MN915018 | MN918597 | Ariyawansa et al. (2020b) |
| *Leucaenicola osmanthi* | NTUCC 18-101-1 | | MN908612 | MN908609 | MN908565 | MN915020 | MN918596 | Ariyawansa et al. (2020b) |
| *Leucaenicola phraeana* | MFLUCC 18-0472 | | MK348003 | MK347892 | MK347785 | MK434867 | MK360060 | Ariyawansa et al. (2020a) |
| *Leucaenicola taiwanensis* | NTUCC 18-094-2 | | MT071275 | MT071226 | MT112299 | MT743285 | MT316124 | Ariyawansa et al. (2020a) |
| *Leucaenicola taiwanensis* | NTUCC 18-094-1 | | MT071277 | MT071228 | MT112301 | MT743284 | MT364370 | Ariyawansa et al. (2020a) |
| *Macrodiplodiopsis desmazieri* | CBS 221.37 | | DQ678065 | – | KR873236 | – | – | Crous et al. (2015) |
| *Macrodiplodiopsis desmazieri* | CBS 140062 | | KR873272 | – | KR873240 | – | – | Crous et al. (2015) |
| *Massarina eburnea* | CBS 473.64 | | GU301840 | GU296170 | – | GU371732 | GU349040 | Schoch et al. (2009) |
| *Palmiascoma gregariascomum* | KUMCC 11-0424 | | – | – | MW075279 | – | – | Monkai et al. (2021) |
| *Palmiascoma gregariascomum* | MFLUCC 11-0175 | | KP744495 | KP753958 | KP744452 | KP998466 | – | Monkai et al. (2021) |
| *Palmiascoma qujingense* | KUMCC 19-0201 | | MT477185 | MT477186 | MT477183 | MT495782 | – | Wang et al. (2022) |
| *Periconia byssoides* | H 4432 | | AB807570 | AB797280 | LC014581 | – | AB808546 | Tanaka et al. (2015) |
| *Periconia pseudobyssoides* | H 4151 | | AB807568 | AB797278 | LC014587 | – | AB808544 | Tanaka et al. (2015) |
| *Stagonospora pseudocaricis* | CBS 135132 | | KF251762 | – | KF251259 | KF252264 | – | Phukhamsakda et al. (2018) |
| *Sulcatispora acerina* | KT 2982 | | LC014610 | LC014605 | LC014597 | – | LC014615 | Wanasinghe et al. (2022) |
| *Sulcatispora berchemiae* | KT 1607 | | AB807534 | AB797244 | AB809635 | – | AB808509 | Wanasinghe et al. (2022) |

Newly generated sequences are in bold, and “–” indicates the sequence unavailability.

**Supplementary Table 5.** Names, voucher numbers, and corresponding GenBank numbers of the taxa used in the phylogenetic analyses of *Montagnula*.

| Species | Voucher number | GenBank accession number | | | | References |
| --- | --- | --- | --- | --- | --- | --- |
|  |  | LSU | SSU | ITS | *TEF*1-α |  |
| *Bimuria novae-zelandiae* | CBS 107.79 | AY016356 | – | MH861181 | DQ471087 | Spatafora et al. (2016) |
| *Bimuria omanensis* | SQUCC 15280 | MT271820 | – | MT274326 | MT279046 | Wijesinghe et al. (2020b) |
| *Deniquelata barringtoniae* | MFLUCC 11-0422 | JX254655 | JX254656 | NR_111779 | – | Ariyawansa et al. (2013) |
| *Deniquelata quercina* | ABRIICC 10068 | MH316157 | MH316155 | MH316153 | – | Alidadi et al. (2019) |
| *Didymocrea leucaenae* | MFLUCC 17-0896 | NG_066304 | MK347826 | NR_164298 | MK360052 | Dubey (2021) |
| *Didymocrea sadasivanii* | CBS 438.65 | DQ384103 | DQ384066 | MH870299 | – | Kruys et al. (2006) |
| *Fuscostagonospora cytisi* | MFLUCC 16-0622 | KY770978 | KY770977 | – | KY770979 | Hyde et al. (2017) |
| *Fuscostagonospora sasae* | HHUF 29106 | NG_059395 | NG_061003 | NR_153964 | AB808524 | Tanaka et al. 2015 |
| *Letendraea cordylinicola* | MFLUCC 11-0148 | NG_059530 | NG_068362 | NR_154118 | – | Ariyawansa et al. (2014) |
| *Letendraea helminthicola* | CBS 884.85 | AY016362 | AY016345 | MK404145 | MK404174 | Ariyawansa et al. (2014) |
| *Montagnula acaciae* | MFLUCC 18-1636 | ON117298 | ON117267 | ON117280 | ON158093 | Tennakoon et al. (2022) |
| *Montagnula acaciae* | NCYUCC 19-0087 | ON117299 | ON117268 | ON117281 | ON158094 | Tennakoon et al. (2022) |
| *Montagnula aloes* | CBS 132531 | NG_042676 | – | NR_111757 | – | Crous et al. (2012) |
| *Montagnula appendiculata* | CBS 109027 | AY772016 | – | DQ435529 | – | Wanasinghe et al. (2016a) |
| *Montagnula aquatica* | MFLU 22-0171 | OP605986 | OP600504 | OP605992 | – | Sun et al. (2023) |
| *Montagnula bellevaliae* | MFLUCC 14-0924 | KT443902 | KT443904 | KT443906 | KX949743 | Hongsanan et al. (2015) |
| *Montagnula camporesii* | MFLUCC 16-1369 | NG_070946 | NG_068418 | MN401746 | MN397908 | Hyde et al. (2020) |
| *Montagnula chiangraiensis* | MFLUCC 17-1420 | NG_068707 | NG_070155 | NR_168864 | – | Mapook et al. (2020) |
| *Montagnula chromolaenae* | MFLUCC 17-1435 | NG_068708 | NG_070156 | NR_168865 | – | Mapook et al. (2020) |
| *Montagnula cirsii* | MFLUCC 13-0680 | KX274249 | KX274255 | KX274242 | KX284707 | Hyde et al. (2016) |
| ***Montagnula coffeae*** | **KUNCC24-18337** | **OR428421** | **OR428379** | **OR428399** | **OR509753** | **This study** |
| ***Montagnula coffeae*** | **KUNCC24-18338** | **OR428422** | **OR428380** | **OR428400** | **OR509754** | **This study** |
| *Montagnula cylindrospora* | UTHSC DI16-208 | LN907351 | – | LT796834 | LT797074 | Crous et al. (2020) |
| *Montagnula donacina* | HFG07004 | MF183940 | – | MF967419 | – | Zhao et al. (2017) |
| *Montagnula donacina* | HKAS 124552 | OP605987 | – | OP605991 | – | Sun et al. (2023) |
| *Montagnula donacina* | HVVV01 | KJ628377 | KJ628376 | KJ628375 | – | Pitt et al. (2014) |
| *Montagnula donacina* | KUMCC 21-0579 | OP059054 | OP059005 | OP058963 | OP135940 | Ren et al. (2021) |
| *Montagnula donacina* | KUMCC 21-0631 | OP059053 | OP059004 | OP058962 | OP135939 | Ren et al. (2021) |
| *Montagnula donacina* | KUMCC 21-0653 | OP059052 | OP059003 | OP058961 | OP135938 | Ren et al. (2021) |
| *Montagnula donacina (M. chromolaenicola)* | MFLUCC 17-1469 | NG_070948 | NG_070157 | NR_168866 | MT235773 | Mapook et al. (2020) |
| *Montagnula donacina (M. puerensis)* | KUMCC 20-0225 | MW575866 | MW575864 | MW567739 | MW573959 | Du et al. (2021) |
| *Montagnula donacina (M. puerensis)* | KUMCC 20-0331 | MW575867 | MW575865 | MW567740 | MW573960 | Du et al. (2021) |
| *Montagnula donacina (M. saikhuensis)* | MFLUCC 16-0315 | KU743210 | KU743211 | KU743209 | – | Wanasinghe et al. (2016a) |
| *Montagnula donacina (M. thailandica)* | MFLUCC 17-1508 | NG_070949 | NG_070158 | MT214352 | MT235774 | Mapook et al. (2020) |
| *Montagnula donacina (M. thailandica)* | MFLUCC 21-0075 | MZ538549 | – | MZ538515 | – | Lu et al. (2022a) |
| *Montagnula donacina (M. thailandica)* | ZHKUCC 22-0206 | OP297777 | OP297791 | OP297807 | OP321576 | Lu et al. (2022a) |
| *Montagnula donacina (M. thailandica)* | ZHKUCC 22-0207 | OP297778 | OP297792 | OP297808 | OP321577 | Boonmee et al. (2021) |
| *Montagnula graminicola* | MFLUCC 13-0352 | KM658315 | KM658316 | KM658314 | – | Liu et al. (2015) |
| *Montagnula guiyangensis* | HGUP 800 | OP600485 | OP600501 | OP605990 | – | Sun et al. (2023) |
| *Montagnula guiyangensis* | HKAS 124556 | OP600484 | OP600500 | OP605989 | – | Sun et al. (2023) |
| *Montagnula jonesii* | MFLU 18-0084 | ON117300 | ON117269 | ON117282 | ON158095 | Tennakoon et al. (2022) |
| *Montagnula jonesii* | MFLUCC 16-1448 | KY273276 | KY313618 | KY313619 | KY313620 | Tennakoon et al. (2016) |
| *Montagnula krabiensis* | MFLUCC 16-0250 | NG_068826 | NG_068385 | NR_168179 | MH412776 | Tibpromma et al. (2018) |
| *Montagnula opulenta* | CBS 168.34 | NG_027581 | AF164370 | AF383966 | LT797074 | Liew et al. (2002) |
| *Montagnula scabiosae* | MFLUCC 14-0954 | KT443903 | KT443905 | KT443907 | – | Hongsanan et al. (2015) |
| *Neokalmusia brevispora* | KT 2313 | AB524601 | AB524460 | NR_154262 | AB539113 | Tanaka et al. (2009) |
| *Neokalmusia kunmingensis* | KUMCC 18-0120 | MK079889 | MK079887 | MK079886 | MK070172 | Hyde et al. (2020) |
| *Neptunomyces aureus* | CMG10A | – | – | MK912119 | MK947998 | Goncalves et al. (2019) |
| *Neptunomyces aureus* | CMG14 | – | – | MK912123 | MK948002 | Goncalves et al. (2019) |
| *Paramassariosphaeria anthostomoides* | CBS 615.86 | MH873693 | GU205246 | MH862005 | – | Wanasinghe et al. (2016a) |
| *Paramassariosphaeria clematidicola* | MFLU 16-0172 | KU743207 | KU743208 | KU743206 | – | Wanasinghe et al. (2016a) |
| *Phaeodothis winteri* | CBS 182.58 | GU301857 | GU296183 | – | – | Schoch et al. (2009) |
| *Pseudopithomyces chartarum* | NCYUCC 19-0168 | MW063220 | MW079349 | MW063159 | – | Zeng et al. (2024) |
| *Pseudopithomyces palmicola* | MFLUCC 17-1506 | MT214447 | – | MT214353 | – | Mapook et al. (2020) |
| *Spegazzinia deightonii* | yone 212 | AB807582 | AB797292 | – | AB808558 | Samarakoon et al. (2020) |
| *Spegazzinia tessarthra* | SH 287 | AB807584 | – | JQ673429 | AB808560 | Samarakoon et al. (2020) |
| *Tremateia arundicola* | MFLU 16-1275 | KX274248 | KX274254 | KX274241 | KX284706 | Wanasinghe et al. (2016a) |
| *Tremateia guiyangensis* | GZAAS01 | KX274247 | KX274253 | KX274240 | KX284705 | Hyde et al. (2016) |
| *Tremateia murispora* | GZCC 18-2787 | MK972751 | MK972750 | NR_165916 | MK986482 | Feng et al. (2019) |

Newly generated sequences are in bold, and “–” indicates the sequence unavailability.

**Supplementary Table 6**. Names, voucher numbers, and corresponding GenBank numbers of the taxa used in the phylogenetic analyses of *Xenodidymella*.

| Species | Voucher number | GenBank accession number | | | | References |
| --- | --- | --- | --- | --- | --- | --- |
|  |  | ITS | LSU | *TUB* | *RPB*2 |  |
| *Ascochyta nigripycnidia* | CBS 116.96 | GU237756 | GU238118 | GU237637 | MT018253 | Aveskamp et al. (2010) |
| *Ascochyta phacae* | CBS 184.55 | KT389475 | KT389692 | KT389769 | MT018255 | Chen et al. (2015) |
| *Ascochyta pilosella* | CBS 583.97 | MN973590 | MN943796 | MT005696 | MT018258 | Hou et al. (2020) |
| *Ascochyta pisi* | CBS 122785 | GU237763 | GU237969 | GU237532 | MT018244 | Aveskamp et al. (2010) |
| *Microsphaeropsis fusca* | CBS 116670 | MN973573 | MN943779 | MT005676 | MT018220 | Hou et al. (2020) |
| *Microsphaeropsis olivacea* | CBS 233.77 | GU237803 | GU237988 | GU237549 | MT018217 | Hou et al. (2020) |
| *Microsphaeropsis olivacea* | CBS 320.76 | MN973568 | MN943775 | MT005671 | MT018214 | Hou et al. (2020) |
| *Microsphaeropsis proteae* | CBS 111319 | JN712497 | JN712563 | MT005679 | MT018223 | Crous et al. (2011) |
| *Microsphaeropsis proteae* | CBS 111303 | JN712495 | JN712561 | MT005677 | MT018221 | Crous et al. (2011) |
| *Microsphaeropsis axicola* | CBS 442.83 | GU237865 | EU754171 | GU237547 | MT018211 | Aveskamp et al. (2010) |
| *Microsphaeropsis viridis* | CBS 639.80 | MN973564 | MN943771 | MT005667 | MT018208 | Hou et al. (2020) |
| *Microsphaeropsis viridis* | CBS 432.71 | GU237863 | GU237987 | GU237548 | MT018209 | Aveskamp et al. (2010) |
| *Neodidymelliopsis cannabis* | CBS 121.75 | GU237761 | GU237972 | GU237535 | MT018288 | Aveskamp et al. (2010) |
| *Paramicrosphaeropsis ellipsoidea* | CBS 197.97 | MN973574 | MN943780 | MT005680 | MT018224 | Hou et al. (2020) |
| *Paramicrosphaeropsis ellipsoidea* | CBS 194.97 | MN973575 | MN943781 | MT005681 | MT018225 | Hou et al. (2020) |
| *Paramicrosphaeropsis iranica* | IRAN 2929C | OK257016 | OK257024 | OK247743 | OK247737 | Ahmadpour et al. (2022a) |
| *Paramicrosphaeropsis iranica* | SCUA-Ah-B | OK257017 | OK257025 | OK247744 | OK247738 | Ahmadpour et al. (2022a) |
| *Xenodidymella applanata* | CBS 205.63 | GU237798 | GU237998 | GU237556 | KP330402 | Aveskamp et al. (2010) |
| *Xenodidymella applanata* | CBS 195.36 | KT389548 | KT389764 | KT389852 | MT018280 | Chen et al. (2015) |
| *Xenodidymella asphodeli* | CBS 499.72 | KT389550 | KT389766 | KT389853 | MT018282 | Chen et al. (2015) |
| *Xenodidymella asphodeli* | CBS 375.62 | KT389549 | KT389765 | MT005716 | KT389689 | Chen et al. (2015) |
| *Xenodidymella camporesii* | MFLUCC 17-2309 | NR169976 | MN244168 | MN871955 | – | Hyde et al. (2020) |
| *Xenodidymella catariae* | CBS 102635 | GU237727 | GU237962 | GU237524 | KP330404 | Aveskamp et al. (2010) |
| *Xenodidymella clematidis* | MFLUCC 16-1365 | MT310600 | MT214553 | – | – | Phukhamsakda et al. (2020) |
| ***Xenodidymella coffeae*** | **KUNCC24-18339** | **OR428407** | **OR428429** | **OR509743** | **OR515527** | **This study** |
| ***Xenodidymella coffeae*** | **KUNCC24-18340** | **OR428408** | **OR428430** | **OR509744** | **OR515528** | **This study** |
| *Xenodidymella glycyrrhizicola* | CBS 141234 | MN973607 | MN943816 | MT005718 | MT018284 | Hou et al. (2020) |
| *Xenodidymella glycyrrhizicola* | CBS 684.97 | MN973606 | MN943815 | MT005717 | MT018283 | Hou et al. (2020) |
| *Xenodidymella humicola* | CBS 220.85 | GU237800 | GU238086 | GU237617 | KP330422 | Aveskamp et al. (2010) |
| *Xenodidymella iranica* | SCUA-Ahm-Sh1-2 | MZ145253 | – | MZ169400 | – | Ahmadpour et al. (2022b) |
| *Xenodidymella iranica* | IRAN 4142C | MZ145252 | – | MZ169399 | – | Ahmadpour et al. (2022b) |
| *Xenodidymella menthae* | IRAN 4375C | OK257018 | OK257026 | OK247745 | OK247739 | Ahmadpour et al. (2022b) |
| *Xenodidymella menthae* | SCUA-Ah-W4-2 | OK257019 | OK257027 | OK247746 | OK247740 | Ahmadpour et al. (2022a) |
| *Xenodidymella weymaniae* | CBS 144960 | MN823588 | MN823439 | MN824762 | MN824613 | Hou et al. (2020) |

Newly generated sequences are in bold, and “–” indicates the sequence unavailability.

**Supplementary Table 7**. Names, voucher numbers, and corresponding GenBank numbers of the taxa used in the phylogenetic analyses of *Flabellascoma*.

| Species | Voucher number | GenBank accession number | | | | | References |
| --- | --- | --- | --- | --- | --- | --- | --- |
|  |  | ITS | LSU | SSU | *RPB*2 | *TEF*1-α |  |
| *Alfoldia vorosii* | REF113 | JN859333 | MK589353 | MK589345 | – | MK599319 | Crous et al. (2019) |
| *Alfoldia vorosii* | REF117 | JN859337 | MK589355 | MK589347 | – | MK599321 | Crous et al. (2019) |
| *Alfoldia vorosii* | CBS 145501 | JN859336 | MK589354 | MK589346 | – | MK599320 | Crous et al. (2019) |
| *Amorocoelophoma camelliae* | NTUCC 18-097-1 | MT112303 | MT071279 | MT071230 | MT459143 | MT743271 | Ariyawansa et al. (2020) |
| *Amorocoelophoma cassiae* | MFLUCC 17-2283 | MK347739 | MK347956 | MK347847 | MK434894 | MK360041 | Jayasiri et al. (2019) |
| *Amorocoelophoma neoregeliae* | CBS 146820 | MZ064410 | MZ064467 | – | MZ078193 | MZ078247 | Crous et al. (2021) |
| *Amorosia littoralis* | CBS 120399 | AM292047 | AM292055 | AM292056 | – | – | Mantle et al. (2006) |
| *Angustimassarina acerina* | MFLUCC 14-0505 | KP899132 | KP888637 | KP899123 | – | KR075168 | Thambugala et al. (2015) |
| *Angustimassarina arezzoensis* | MFLUCC 13-0578 | KY496743 | KY496722 | KY501113 | – | KY514392 | Tibpromma et al. (2017) |
| *Angustimassarina camporesii* | MFLU 18-0057 | MN244197 | MN244167 | MN244173 | – | – | Hyde et al. (2020) |
| *Bambusicola guttulata* | CGMCC 3.20935 | ON332909 | ON332927 | ON332919 | ON383985 | ON381177 | Yu et al. (2022) |
| *Bambusicola guttulata* | UESTCC 22.0002 | ON332910 | ON332928 | ON332920 | ON383986 | ON381178 | Yu et al. (2022) |
| *Crassiclypeus aquaticus* | CBS 143641 | LC312499 | LC312528 | LC312470 | LC312586 | LC312557 | Hashimoto et al. (2018) |
| *Crassiclypeus aquaticus* | CBS 143643 | LC312501 | LC312530 | LC312472 | LC312588 | LC312559 | Hashimoto et al. (2018) |
| *Flabellascoma aquaticum* | KUMCC 15-0258 | MN304827 | MN274564 | MN304832 | MN328895 | MN328898 | Bao et al. (2019) |
| *Flabellascoma cycadicola* | CBS 143644 | LC312502 | LC312531 | LC312473 | LC312589 | LC312560 | Hashimoto et al. (2018) |
| *Flabellascoma fusiforme* | MFLUCC 18-1584 | MN304830 | MN274567 | – | – | MN328902 | Bao et al. (2019) |
| ***Flabellascoma coffeae*** | **KUNCC24-18341** | **OR428405** | **OR428427** | **OR428385** | **OR515525** | **OR509759** | **This study** |
| ***Flabellascoma coffeae*** | **KUNCC24-18342** | **OR428406** | **OR428428** | **OR428386** | **OR515526** | **OR509760** | **This study** |
| ***Flabellascoma coffeae*** | **KUNCC24-18343** | **OR428403** | **OR428425** | **OR428383** | **OR515523** | **OR509757** | **This study** |
| ***Flabellascoma coffeae*** | **KUNCC24-18344** | **OR428404** | **OR428426** | **OR428384** | **OR515524** | **OR509758** | **This study** |
| *Flabellascoma minimum* | CBS 143645 | LC312503 | LC312532 | LC312474 | LC312590 | LC312561 | Hashimoto et al. (2018) |
| *Flabellascoma minimum* | CBS 143646 | LC312504 | LC312533 | LC312475 | LC312591 | LC312562 | Hashimoto et al. (2018) |
| *Flabellascoma sichuanense* | CGMCC 3.20936 | ON332911 | ON332929 | ON332921 | ON383987 | ON381179 | Yu et al. (2022) |
| *Flabellascoma sichuanense* | UESTCC 22.0003 | ON332912 | ON332930 | ON332922 | ON383988 | ON381180 | Yu et al. (2022) |
| *Lophiomurispora hongheensis* | KUMCC 20-0216 | MW264218 | MW264197 | MW264227 | MW256810 | MW256819 | Wanasinghe et al. (2021) |
| *Lophiomurispora hongheensis* | KUMCC 20-0217 | MW264216 | MW264195 | MW264225 | MW256808 | MW256817 | Wanasinghe et al. (2021) |
| *Lophiostoma macrostomum* | KT 709 = HHUF 27293 | AB433276 | AB433274 | AB521732 | JN993493 | LC001753 | Zhang et al. (2009) |
| *Lophiostoma pseudodictyosporium* | MFLUCC 13-0451 | KR025858 | KR025862 | – | – | – | Liu et al. (2015) |
| *Lophiostoma ravennicum* | MFLUCC 14-0005 | KP698413 | KP698414 | KP698415 | – | – | Liu et al. (2015) |
| *Massarina corticola* | CBS 154.93 | – | FJ795448 | FJ795491 | FJ795465 | – | Zhang et al. (2009) |
| *Neoangustimassarina sichuanensis* | CGMCC 3.20937 | ON332907 | ON332925 | ON332917 | ON383983 | ON381175 | Yu et al. (2022) |
| *Neoangustimassarina sichuanensis* | UESTCC 22.0001 | ON332908 | ON332926 | ON332918 | ON383984 | ON381176 | Yu et al. (2022) |
| *Neothyrostroma encephalarti* | CBS 146037 | MN562104 | MN567612 | – | – | MN556830 | Crous et al. (2019) |
| *Neothyrostroma encephalarti* | CPC 35999 | MN562105 | MN567613 | – | – | MN556831 | Crous et al. (2019) |
| *Neovaginatispora clematidis* | MFLUCC 17-2156 | MT310606 | MT214559 | MT226676 | – | MT394738 | Phukhamsakda et al. (2020) |
| *Neovaginatispora fuckelii* | KH 161 | LC001731 | AB619008 | AB618689 | – | LC001749 | Thambugala et al. (2015) |
| *Neovaginatispora fuckelii* | KT 634 | LC001732 | AB619009 | AB618690 | – | LC001750 | Thambugala et al. (2015) |
| *Paucispora quadrispora* | KT843 | LC001734 | AB619011 | AB618692 | – | LC001755 | Thambugala et al. (2015) |
| *Paucispora versicolor* | KH110 | AB918731 | AB918732 | LC001721 | – | LC001760 | Thambugala et al. (2015) |
| *Podocarpomyces knysnanus* | CBS 14676 | MN562155 | MN567662 | – | MN556816 | MN556836 | Crous et al. (2019) |
| *Vaginatispora amygdali* | CBS 143662 | LC312524 | LC312553 | LC312495 | LC312611 | LC312582 | Hashimoto et al. (2018) |
| *Vaginatispora appendiculata* | MFLUCC 16-0314 | KU743217 | KU743218 | KU743219 | – | KU743220 | Wanasinghe et al. (2016b) |
| *Vaginatispora aquatica* | MFLUCC 11-0083 | KJ591577 | KJ591576 | KJ591575 | – | – | Hyde et al. (1995) |

Newly generated sequences are in bold, “–” indicates the sequence unavailability.

**Supplementary Table 8**. Names, voucher numbers, and corresponding GenBank numbers of the taxa used in the phylogenetic analyses of *Longiostiolum.*

| Species | Voucher number | GenBank accession number | | | | Reference |
| --- | --- | --- | --- | --- | --- | --- |
|  |  | ITS | LSU | SSU | *TEF*1-α |  |
| *Aigialus grandis* | BCC 20000 | – | GU479775 | GU479739 | GU479839 | Suetrong et al. (2009) |
| *Aigialus mangrovis* | BCC 33563 | – | GU479776 | GU479741 | GU479840 | Suetrong et al. (2009) |
| *Aigialus parvus* | NFCCI-395 | MK028710 | MK026761 | MK026763 | MN520611 | Devadatha et al. (2018) |
| *Aigialus rhizophorae* | BCC 33572 | – | GU479780 | GU479745 | GU479844 | Suetrong et al. (2009) |
| *Alfoldia vorosii* | REF116 | JN859336 | MK589354 | MK589346 | MK599320 | Crous et al. (2019) |
| *Amorocoelophoma camelliae* | NTUCC 18-097-1 | MT112303 | MT071279 | MT071230 | MT459143 | Ariyawansa et al. (2020) |
| *Amorocoelophoma camelliae* | NTUCC 18-097-2 | MT112304 | MT071280 | MT071231 | MT459141 | Ariyawansa et al. (2020) |
| *Amorocoelophoma cassia* | MFLUCC 17-2283 | NR_163330 | NG_066307 | NG_065775 | MK360041 | Jayasiri et al. (2019) |
| *Amorocoelophoma neoregeliae* | CBS 146820 | MZ064410 | NG_076726 | – | MZ078247 | Crous et al. (2021) |
| *Amorosia littoralis* | NN 6654 | AM292047 | AM292055 | AM292056 | – | Mantle et al. (2006) |
| *Angustimassarina acerina* | MFLUCC 14-0505 | KP899132 | KP888637 | KP899123 | KR075168 | Thambugala et al. (2015) |
| *Angustimassarina alni* | MFLUCC 15-0184 | KY548099 | KY548097 | KY548098 | – | Tibpromma et al. (2017) |
| *Angustimassarina arezzoensis* | MFLUCC 13-0578 | KY496743 | KY496722 | KY501113 | KY514392 | Tibpromma et al. (2017) |
| *Angustimassarina camporesii* | MFLU 18-0057 | NR_168223 | MN244167 | MN244173 | – | Hyde et al. (2020) |
| *Angustimassarina coryli* | MFLUCC 14-0981 | MF167431 | MF167432 | – | MF167433 | Hyde et al. (2017) |
| *Aquasubmersa japonica* | KT2863 | LC061593 | LC061588 | LC061583 | LC194385 | Ariyawansa et al. (2015) |
| *Aquasubmersa japonica* | KT2862 | LC061592 | LC061587 | LC061582 | LC194384 | Ariyawansa et al. (2015) |
| *Botryosphaeria dothidea* | MFLUCC 16-0936 | MT177923 | MT177950 | MT177977 | – | Li et al. (2020) |
| *Botryosphaeria dothidea* | CBS 115476 | NR_111146 | NG_027577 | NG_062738 | – | Zhang et al. (2021) |
| *Crassiperidium octosporum* | KT 2894 | LC373097 | LC373109 | LC373085 | LC373121 | Matsumura et al. (2018) |
| *Crassiperidium octosporum* | KT 3008 | LC373098 | LC373110 | LC373086 | LC373122 | Matsumura et al. (2018) |
| *Crassiperidium octosporum* | KT 2144 | LC373096 | LC373108 | LC373084 | LC373120 | Matsumura et al. (2018) |
| *Crassiperidium quadrisporum* | KT 2798-1 | LC373106 | LC373118 | LC373094 | LC373130 | Matsumura et al. (2018) |
| *Crassiperidium quadrisporum* | KT 2798-2 | LC373107 | LC373119 | LC373095 | LC373131 | Matsumura et al. (2018) |
| *Cucitella opali* | CBS 142405 | MF795754 | MF795754 | MF795837 | MF795843 | Jaklitsch et al. (2018) |
| *Diatrype disciformis* | AFTOL-ID 927 | – | DQ470964 | DQ471012 | DQ471085 | Spatafora et al. (2006) |
| *Diplodia mutila* | AFTOL-ID 1572 | KU198424 | DQ377863 | DQ678012 | DQ677907 | Crous et al. (2006) |
| *Diplodia mutila* | GMBCC1173 | OM855587 | OM855596 | OM855610 | OM857551 | Wijayawardene et al. (2022) |
| *Diplodia seriata* | CBS 119049 | DQ458889 | EU673266 | EU673216 | – | Zhang et al. (2021) |
| *Diplodia seriata* | CBS 112555 | AY259094 | KF766327 | NG_062751 | – | Zhang et al. (2021) |
| *Fenestella media* | CBS 144860 | MK356285 | MK356285 | MK356326 | MK357558 | Jaklitsch et al. (2020) |
| *Graphostroma platystoma* | CBS 270.87 | JX658535 | DQ836906 | DQ836900 | DQ836915 | Stadler et al. (2014) |
| ***Longiostiolum coffeae*** | **KUNCC24-18345** | **OR428397** | **OR428419** | **OR428377** | **OR509751** | **This study** |
| ***Longiostiolum coffeae*** | **KUNCC24-18346** | **OR428398** | **OR428420** | **OR428378** | **OR509752** | **This study** |
| *Longiostiolum tectonae* | MFLU 15-3532 | KU712447 | KU764700 | KU712459 | KU872759 | Li et al. (2016) |
| *Parafenestella rosacearum* | FM1 | MK356313 | MK356313 | MK356327 | MK357585 | Jaklitsch et al. (2020) |
| *Phaeosphaeria chiangraina* | MFLUCC 13-0231 | KM434270 | KM434280 | KM434289 | KM434298 | Phookamsak et al. (2014) |
| *Phaeosphaeria musae* | MFLUCC 11-0133 | KM434267 | KM434277 | KM434287 | KM434296 | Phookamsak et al. (2014) |
| *Phaeosphaeria thysanolaenicola* | MFLUCC 10-0563 | KM434266 | KM434276 | KM434286 | KM434295 | Phookamsak et al. (2014) |
| *Salsuginea ramicola* | KT 2597.1 | – | GU479800 | GU479767 | GU479861 | Suetrong et al. (2009) |
| *Salsuginea ramicola* | KT 2597.2 | – | GU479801 | GU479768 | GU479862 | Suetrong et al. (2009) |
| *Shearia formosa* | MFLUCC 20-0017 | MT159625 | MT159619 | MT159631 | MT159602 | Wanasinghe et al. (2020) |
| *Shearia formosa* | MFLUCC 20-0018 | MT159627 | MT159621 | MT159633 | MT159604 | Wanasinghe et al. (2020) |
| *Shearia formosa* | GMBCC1172 | OM855592 | OM855601 | OM855615 | – | Wijayawardene et al. (2022) |
| *Shearia formosa* | MFLUCC 20-0019 | MT159626 | MT159620 | MT159632 | MT159603 | Wanasinghe et al. (2020) |
| *Sordaria fimicola* | AFTOL-ID 216 | DQ518178 | FR774289 | AH007748 | DQ518175 | James et al. (2006) |
| *Thyrostroma lycii* | MFLUCC 16-1170 | MK751734 | MK751824 | MK751769 | MK908024 | Senwanna et al. (2019) |
| *Thyrostroma tiliae* | MFLUCC 16-1178 | – | MK751828 | MK751773 | MK751738 | Senwanna et al. (2019) |
| *Tzeanania taiwanensis* | NTUCC 17-006 | – | MH461121 | MH461127 | MH461124 | Ariyawansa et al. (2018) |
| *Tzeanania taiwanensis* | NTUCC 17-005 | – | MH461120 | MH461126 | MH461123 | Ariyawansa et al. (2018) |

Newly generated sequences are in bold, “–” indicates the sequence unavailability.

**Supplementary Table 9**. Names, voucher numbers, and corresponding GenBank numbers of the taxa used in the phylogenetic analyses of *Helminthosporium*.

| Species | Voucher number | GenBank accession number | | | | | References |
| --- | --- | --- | --- | --- | --- | --- | --- |
|  |  | SSU | LSU | ITS | *RPB*2 | *TEF*1-α |  |
| *Byssothecium circinans* | CBS 675.92 | GU205235 | GU205217 | OM337536 | DQ767646 | GU349061 | Hu et al. (2009) |
| *Helminthosporium aquaticum* | S-096 = MFLUCC 15-0357 | KU697310 | KU697306 | KU697302 | – | – | Zhu et al. (2016) |
| *Helminthosporium austriacum* | L132 = CBS 139924 | KY984420 | KY984301 | KY984301 | KY984365 | KY984437 | Voglmayr and Jaklitsch (2017) |
| *Helminthosporium austriacum* | L169 = CBS 142388 | – | KY984303 | KY984303 | KY984367 | KY984439 | Voglmayr and Jaklitsch (2017) |
| *Helminthosporium caespitosum* | L99 = CBS 484.77 | KY984421 | JQ044448 | JQ044429 | KY984370 | KY984440 | Voglmayr and Jaklitsch (2017) |
| *Helminthosporium caespitosum* | L141 | – | KY984305 | KY984305 | KY984368 | – | Voglmayr and Jaklitsch (2017) |
| *Helminthosporium chengduense* | UESTC 22.0024 = CGMCC 3.23575 | ON557757 | ON557745 | ON557751 | ON563073 | ON600598 | Chen et al. (2022) |
| *Helminthosporium chengduense* | UESTC 22.0025 | ON557756 | ON557744 | ON557750 | ON563072 | ON600597 | Chen et al. (2022) |
| *Helminthosporium chiangraiense* | MFLUCC 21-0087 | – | MZ538538 | MZ538504 | – | – | Boonmee et al. (2021) |
| *Helminthosporium chinense* | UESTCC 22.0026 = CGMCC 3.23570 | ON557760 | ON557748 | ON557754 | – | ON600601 | Chen et al. (2022) |
| *Helminthosporium chlorophorae* | BRIP 14521 | – | – | AF120259 | – | – | Olivier et al. (2000) |
| *Helminthosporium dalbergiae* | H4628 = MAFF 243853 | AB797231 | AB807521 | LC014555 | – | AB808497 | Tanaka et al. (2015) |
| *Helminthosporium endiandrae* | CBS 138902 = CPC 22194 | – | KP004478 | KP004450 | – | – | Crous et al. (2014) |
| *Helminthosporium erythrinicola* | CBS 145569 = CPC 35291 | – | MK876432 | NR_165563 | MK876486 | – | Crous et al. (2019) |
| *Helminthosporium genistae* | L129 = CBS 139922 | KY984423 | KY984309 | KY984309 | KY984373 | – | Voglmayr and Jaklitsch (2017) |
| *Helminthosporium genistae* | L142 = CBS142597 | – | KY984310 | KY984310 | KY984374 | – | Voglmayr and Jaklitsch (2017) |
| *Helminthosporium genistae* | L143 = CBS 139927 | – | KY984311 | KY984311 | KY984375 | – | Voglmayr and Jaklitsch (2017) |
| *Helminthosporium genistae* | L144 = CBS 139928 | – | KY984312 | KY984312 | KY984376 | – | Voglmayr and Jaklitsch (2017) |
| *Helminthosporium genistae* | L148 = CBS 139929 | – | KY984315 | KY984315 | KY984379 | – | Voglmayr and Jaklitsch (2017) |
| *Helminthosporium genistae* | L149 = CBS 139930 | – | KY984316 | KY984316 | KY984380 | – | Voglmayr and Jaklitsch (2017) |
| *Helminthosporium guanshanense* | HJAUP C1022 | OQ172247 | OQ172239 | OQ172249 | OQ234978 | OQ256247 | Hu et al. (2023) |
| *Helminthosporium hispanicum* | L109 = CBS 136917 | KY984424 | KY984318 | KY984318 | KY984381 | KY984441 | Voglmayr and Jaklitsch (2017) |
| *Helminthosporium jiulianshanense* | HJAUP C1057 | – | OQ172253 | OQ172245 | OQ234979 | – | Hu et al. (2023) |
| *Helminthosporium juglandinum* | L101 = CBS 136912 | – | KY984319 | KY984319 | KY984382 | KY984442 | Voglmayr and Jaklitsch (2017) |
| *Helminthosporium juglandinum* | L102 = CBS 136913 | – | KY984320 | KY984320 | – | – | Voglmayr and Jaklitsch (2017) |
| *Helminthosporium juglandinum* | L118 = CBS 136922 | – | KY984321 | KY984321 | KY984383 | KY984443 | Voglmayr and Jaklitsch (2017) |
| *Helminthosporium juglandinum* | L97 = CBS 136911 | KY984425 | KY984322 | KY984322 | – | – | Voglmayr and Jaklitsch (2017) |
| *Helminthosporium leucadendri* | CBS 135133 = CPC19345 | – | KF251654 | KF251150 | KF252159 | KF253110 | Quaedvlieg et al. (2013) |
| *Helminthosporium livistonae* | CPC 32158 = CBS144413 | – | NG_064539 | NR_160348 | – | – | Crous et al. (2018b) |
| *Helminthosporium magnisporum* | H4627 = MAFF 239278 = TS 33 | AB797232 | AB807522 | AB811452 | – | AB808498 | Tanaka et al. (2015) |
| *Helminthosporium massarinum* | KT 838 = MAFF239604 | AB797233 | AB807523 | AB809628 | – | AB808499 | Tanaka et al. (2015) |
| *Helminthosporium massarinum* | KT 1564 = MAFF 239605 | AB797234 | AB807524 | AB809629 | – | AB808500 | Tanaka et al. (2015) |
| *Helminthosporium meilingense* | HJAUP C1076 | OQ172246 | OQ172238 | OQ172244 | OQ234980 | OQ234981 | Hu et al. (2023) |
| *Helminthosporium microsorum* | L94 | KY984426 | KY984327 | KY984327 | KY984388 | KY984446 | Voglmayr and Jaklitsch (2017) |
| *Helminthosporium microsorum* | L95 | – | KY984328 | KY984328 | KY984389 | KY984447 | Voglmayr and Jaklitsch (2017) |
| *Helminthosporium microsorum* | L96 = CBS 136910 | KY984427 | KY984329 | KY984329 | KY984390 | KY984448 | Voglmayr and Jaklitsch (2017) |
| *Helminthosporium nabanhense* | HJAUP C2054 | OP555400 | OP555398 | OP555394 | – | OP961931 | Liu et al. (2022) |
| *Helminthosporium nanjingense* | ZM020380 = HHAUF020380 | – | – | KF192322 | – | – | Wang et al. (2014) |
| *Helminthosporium oligosporum* | L92 = CBS 136908 | KY984428 | KY984332 | KY984332 | KY984393 | KY984450 | Voglmayr and Jaklitsch (2017) |
| *Helminthosporium oligosporum* | L93 = CBS 136909 | – | KY984333 | KY984333 | KY984394 | KY984451 | Voglmayr and Jaklitsch (2017) |
| ***Helminthosporium puerensis*** | **KUNCC24-18347** | **OR428371** | **OR428413** | **OR428391** | **OR515515** | **OR509745** | **This study** |
| ***Helminthosporium puerensis*** | **KUNCC24-18348** | **OR428372** | **OR428414** | **OR428392** | **OR515516** | **OR509746** | **This study** |
| *Helminthosporium quercinum* | ZT-97034 = CBS 112393 | – | KY984334 | KY984334 | KY984395 | KY984452 | Voglmayr and Jaklitsch (2017) |
| *Helminthosporium quercinum* | L107 = CBS 136915 | – | KY984336 | KY984336 | KY984397 | – | Voglmayr and Jaklitsch (2017) |
| *Helminthosporium quercinum* | L90 = CBS 136921 | KY984429 | KY984339 | KY984339 | KY984400 | KY984453 | Voglmayr and Jaklitsch (2017) |
| *Helminthosporium sinense* | HJAUP C2121 | OP555399 | OP555397 | OP555393 | – | OP961932 | Liu et al. (2022) |
| *Helminthosporium solani* | CBS 365.75 | KY984430 | KY984341 | KY984341 | KY984402 | KY984455 | Voglmayr and Jaklitsch (2017) |
| *Helminthosporium solani* | CBS 640.85 | – | KY984342 | KY984342 | KY984403 | – | Voglmayr and Jaklitsch (2017) |
| *Helminthosporium submersum* | MFLUCC 16-1360 | MG098796 | MG098787 | – | – | MG098586 | Zhao et al. (2018) |
| *Helminthosporium submersum* | MFLUCC 16-1290 | MG098797 | MG098788 | MG098780 | MG098592 | MG098587 | Zhao et al. (2018) |
| *Helminthosporium syzygii* | CPC35312 = CBS 145570 | – | MK876433 | NR_165564 | MK876487 | – | Crous et al. (2019) |
| *Helminthosporium tiliae* | L87 = CBS 136906 | – | KY984344 | KY984344 | KY984405 | – | Voglmayr and Jaklitsch (2017) |
| *Helminthosporium tiliae* | L88 = CBS 136907 | KY984431 | KY984345 | KY984345 | KY984406 | KY984457 | Voglmayr and Jaklitsch (2017) |
| *Helminthosporium velutinum* | H4626 | AB797240 | AB807530 | LC014556 | – | AB808505 | Tanaka et al. (2015) |
| *Helminthosporium velutinum* | H4739 | AB797235 | AB807525 | LC014557 | – | AB808501 | Tanaka et al. (2015) |
| *Helminthosporium velutinum* | L115 = CBS 136924 | – | KY984347 | KY984347 | KY984408 | KY984458 | Voglmayr and Jaklitsch (2017) |
| *Helminthosporium velutinum* | L131 = CBS 139923 | KY984432 | KY984352 | KY984352 | KY984413 | KY984463 | Voglmayr and Jaklitsch (2017) |
| *Helminthosporium velutinum* | L98 | KY984433 | KY984359 | KY984359 | KY984417 | KY984466 | Voglmayr and Jaklitsch (2017) |
| *Helminthosporium yunnanense* | HJAUP C2071 | OP555392 | OP555396 | OP555395 | OP961934 | OP961933 | Liu et al. (2022) |
| *Helminthosporium shangrilaense* | KUNCC22-12540 | OP767127 | OP767126 | OP767128 | – | OQ186449 | Hyde et al. (2023) |
| *Helminthosporium lignicolum* | MFLUCC 22- 0118 | OP740253 | OP740252 | ON329811 | OP757656 | OP757657 | Hyde et al. (2023) |
| *Massarina cisti* | CBS 266.62 = JCM 14140 | AB797249 | AB807539 | LC014568 | – | AB808514 | Tanaka et al. (2015) |
| *Massarina eburnea* | CBS 473.64 | AF164367 | GU301840 | AF383959 | GU371732 | GU349040 | Schoch et al. (2009) |
| *Massarina eburnea* | H3953 = CBS 139697 | AB521718 | AB521735 | LC014569 | – | AB808517 | Hirayama et al. (2010) |
| *Periconia digitata* | CBS 510.77 | AB797271 | AB807561 | LC014584 | – | AB808537 | Tanaka et al. (2015) |
| *Periconia pseudodigitata* | KT 1395 = CBS139699 | NG_064850 | NG_059396 | NR_153490 | – | AB808540 | Tanaka et al. (2015) |
| *Pseudosplanchnonema phorcioides* | L16 = CBS 122935 | KY984434 | KY984360 | KY984360 | KY984418 | KY984467 | Voglmayr and Jaklitsch (2017) |
| *Stagonospora paludosa* | CBS 135088 | – | KF251760 | KF251257 | KF252262 | KF253207 | Quaedvlieg et al. (2013) |
| *Stagonospora perfecta* | KT 1726A = MAFF 239609 | AB797289 | AB807579 | AB809642 | – | AB808555 | Tanaka et al. (2015) |
| *Stagonospora pseudoperfecta* | KT 889 = CBS 120.236 = MAFF 239607 | AB797287 | AB807577 | AB809641 | – | AB808553 | Tanaka et al. (2015) |
| *Stagonospora tainanensis* | KT 1866 = MAFF 243860 | AB797290 | AB807580 | AB809643 | – | AB808556 | Tanaka et al. (2015) |

Newly generated sequences are in bold, and “–” indicates the sequence unavailability.

**Supplementary Table 10**. Names, voucher numbers, and corresponding GenBank numbers of the taxa used in the phylogenetic analyses of *Neomassaria*.

| Species | Voucher number | GenBank accession number | | | | | References |
| --- | --- | --- | --- | --- | --- | --- | --- |
|  |  | ITS | LSU | SSU | *TEF*1-α | *RPB2* |  |
| *Acrocordiopsis patilii* | BCC28167 | – | GU479773 | GU479737 | – | GU479812 | Kohlmeyer et al. (2009) |
| *Acrocordiopsis patilii* | BCC28166 | – | GU479772 | GU479736 | – | GU479811 | Kohlmeyer et al. (2009) |
| *Acuminatispora palmarum* | MFLUCC 18-0460 | MN749106 | MH390438 | MH390402 | MH399249 | MH399252 | Zhang et al. (2018) |
| *Acuminatispora palmarum* | MFLUCC 18-0461 | MN749107 | MH390439 | MH390403 | MH399250 | MH399253 | Zhang et al. (2018) |
| *Aigialus grandis* | BCC20000 | – | GU479775 | GU479739 | GU479839 | GU479814 | Suetrong et al. (2009) |
| *Aigialus grandis* | BCC18419 | – | GU479774 | GU479738 | GU479838 | GU479813 | Suetrong et al. (2009) |
| *Ascocratera manglicola* | BCC09270 | – | GU479782 | GU479747 | GU479846 | GU479821 | Suetrong et al. (2009) |
| *Astrosphaeriella neofusispora* | MFLUCC 11-0161 | – | KT955463 | KT955444 | – | KT955418 | Phookamsak et al. (2015) |
| *Astrosphaeriellopsis bakeriana* | CBS115556 | JN846716 | GU301801 | – | GU349015 | – | Phookamsak et al. (2015) |
| *Berkleasmium longisporum* | MFLUCC 17-1999 | MH558698 | MH558825 | – | MH550889 | MH551012 | Lu et al. (2018) |
| *Berkleasmium thailandicum* | MFLUCC 17-2000 | MH558701 | MH558828 | – | MH550892 | MH551016 | Lu et al. (2018) |
| *Caryospora aquatica* | MFLUCC 11-0008 | MH057848 | MH057847 | MH057850 | – | – | Yang et al. (2023) |
| *Caryospora minima* | 492516 | – | EU196550 | EU196551 | – | – | Cai et al. (2007) |
| *Delitschia chaetomioides* | SMH 3253.2 | – | GU390656 | – | GU327753 | – | Mugambi et al. (2009) |
| *Delitschia winteri* | CBS 225.62 | – | DQ678077 | DQ678026 | DQ677922 | DQ677975 | Schoch et al. (2006) |
| *Fusculina eucalyptorum* | CBS 145083 | MK047449 | MK047499 | – | – | – | Crous et al. (2018c) |
| *Fusculina regnans* | CBS 143428 | MN161917 | MN162186 | – | – | – | Crous et al. (2019) |
| *Gordonomyces mucovaginatus* | CBS 127273 | MH864326 | NG_057941 | – | – | – | Crous et al. (2011) |
| *Lindgomyces angustiascus* | A640-1a | JX508281 | JX508279 | JX508280 | – | – | Raja et al. (2013) |
| *Lindgomyces apiculatus* | KT1108 | JF419892 | JF419884 | JF419886 | – | – | Raja et al. (2011) |
| *Lindgomyces aquaticus* | MFLUCC 18-1416 | MT627692 | MN913727 | MT864317 | – | MT878457 | Dong et al. (2020) |
| *Lindgomyces breviappendiculatus* | KT1399 | JF419897 | AB521749 | AB521734 | – | – | Hirayama et al. (2010) |
| *Lindgomyces carolinensis* | DSMZ103499G618 | KX655793 | KX655800 | KX655801 | – | – | Raja et al. (2017) |
| *Lindgomyces cigarospora* | G619 | KX655794 | KX655804 | KX655805 | – | – | Raja et al. (2017) |
| *Lindgomyces cinctosporus* | R56-1 | JF419905 | AB522431 | AB522430 | – | – | Hirayama et al. (2010) |
| *Lindgomyces ingoldianus* | ATCC200398 | – | AB521736 | AB521719 | – | – | Hirayama et al. (2010) |
| *Lindgomyces lemonweirensis* | A632-1a | JF419894 | JF419888 | JF419890 | – | – | Raja et al. (2011) |
| *Lindgomyces madisonensis* | G416-a | KT207818 | KT207820 | KT207822 | – | – | Crous et al. (2015) |
| *Lindgomyces okinawaensis* | KT3531 | LC100022 | LC100027 | LC100019 | – | – | Li et al. (2016) |
| *Lindgomyces pseudomadisonensis* | KT2742 | LC149914 | LC149916 | LC149912 | – | – | Hyde et al. (2016) |
| *Lindgomyces rotundatus* | KT966 | JF419901 | AB521739 | AB521722 | – | – | Hirayama et al. (2010) |
| *Lophiostoma heterosporum* | CBS 644.86 | – | AY016369 | AY016345 | – | – | Lumbsch et al. (2001) |
| *Lophiostoma macrostomum* | KT635 | – | AB433273 | AB521731 | – | JN993484 | Tanaka et al. (2008) |
| *Lophiostoma macrostomum* | KT709 | – | AB433274 | AB521732 | – | JN993493 | Tanaka et al. (2008) |
| *Massaria anomia* | CBS 591.78 | HQ599379 | GU301839 | GU296169 | – | GU371769 | Voglmayr et al. (2015) |
| *Massaria ariae* | M52 | – | HQ599382 | HQ599456 | HQ599322 | – | Voglmayr et al. (2015) |
| *Massaria ariae* | M9 | – | HQ599381 | HQ599458 | HQ599321 | – | Voglmayr et al. (2015) |
| *Massaria aucupariae* | M37 | – | HQ599383 | HQ599451 | HQ599323 | – | Voglmayr et al. (2015) |
| *Massaria aucupariae* | M49 | – | HQ599384 | HQ599455 | HQ599324 | – | Voglmayr et al. (2015) |
| *Massaria campestris* | M30 | – | HQ599387 | – | HQ599327 | – | Voglmayr et al. (2015) |
| *Massaria campestris* | M29 | – | HQ599386 | – | HQ599326 | – | Voglmayr et al. (2015) |
| *Massaria conspurcata* | WU30515 | – | HQ599394 | – | HQ599334 | – | Voglmayr et al. (2015) |
| *Massaria gigantispora* | M26 | HQ599397 | HQ599397 | HQ599447 | HQ599337 | – | Voglmayr et al. (2015) |
| *Massaria inquinans* | M19 | HQ599402 | HQ599402 | HQ599444 | HQ599342 | HQ599460 | Voglmayr et al. (2015) |
| *Neomassaria alstoniae* | MFLUCC 21-0213 | – | OL457711 | OL764416 | – | – | de Silva et al. (2022) |
| ***Neomassaria coffeae*** | **KUNCC24-18349** | **OR428411** | **OR428433** | **OR428389** | **OR509763** | **OR515529** | **This study** |
| ***Neomassaria coffeae*** | **KUNCC24-18350** | **OR428412** | **OR428434** | **OR428390** | **OR509764** | **OR515530** | **This study** |
| *Neomassaria fabacearum* | MFLUCC 15-0372 | – | MG837003 | MG837004 | – | – | Hyde et al. (2016) |
| *Neomassaria fabacearum* | MFLUCC 16-1875 | – | KX524145 | KX524147 | KX524149 | – | Hyde et al. (2016) |
| *Neomassaria formosana* | NTUCC 17-009 | – | MH714758 | MH714761 | MH714764 | MH714767 | Ariyawansa et al. (2018) |
| *Neomassaria formosana* | NTUCC 17-008 | – | MH714757 | MH714760 | MH714763 | MH714766 | Ariyawansa et al. (2018) |
| *Neomassaria formosana* | NTUCC 17-007 | – | MH714756 | MH714759 | MH714762 | MH714765 | Ariyawansa et al. (2018) |
| *Neomassaria hongheensis* | KUMCC 21-0344 | OL477614 | OL423113 | OL423115 | OL754594 | OL754595 | Yang et al. (2022) |
| *Neomassaria hongheensis* | KUMCC 21-0340 | OL477594 | OL423112 | OL423114 | OL754592 | OL754593 | Yang et al. (2022) |
| *Neomassaria thailandica* | MFLUCC 21-0193 | – | OL457712 | OL700224 | ON032376 | – | de Silva et al. (2022) |
| *Pigmentatineomassaria italica* | MFLU 19-0948 | – | OQ411138 | OQ411132 | OQ420449 | OQ420450 | Wijesinghe et al. (2023) |
| *Quercicola fusiformis* | MFUCC 18-0479 | MK347790 | MK348009 | MK347898 | MK360085 | MK434864 | Jayasiri et al. (2019) |
| *Quercicola guttulospora* | MFUCC 18-0481 | MK347791 | MK348010 | MK347899 | MK360086 | – | Jayasiri et al. (2019) |
| *Salsuginea ramicola* | KT2597.2 | – | GU479801 | GU479768 | GU479862 | GU479834 | Suetrong et al. (2009) |
| *Salsuginea ramicola* | KT2597.1 | – | GU479800 | GU479767 | GU479861 | GU479833 | Suetrong et al. (2009) |
| *Zopfiar hizophila* | CBS 207.26 | – | DQ384104 | – | – | – | Kruys et al. (2006) |

Newly generated sequences are in bold, “–” indicates the sequence unavailability.

**Supplementary Table 11**. Names, voucher numbers, and corresponding GenBank numbers of the taxa used in the phylogenetic analyses of *Neooccultibambusa*.

| Species | Voucher number | GenBank accession number | | | | | | | | | References |
| --- | --- | --- | --- | --- | --- | --- | --- | --- | --- | --- | --- |
|  |  | LSU | SSU | | ITS | | *RPB*2 | | *TEF*1-a | |  |
| *Brunneofusispora clematidis* | MFLUCC 17-2070 | MT214570 | | MT226685 | | MT310615 | | MT394692 | | MT394629 | Phukhamsakda et al. (2020) |
| *Brunneofusispora hyalina* | MFLUCC 21-0008 | MW287234 | | MW485613 | | MW260330 | | MW512609 | | MW512606 | Calabon et al. (2021) |
| *Brunneofusispora inclinatiostiola* | CGMCC 3.20403 | MZ964875 | | MZ964884 | | MZ964866 | | OK061075 | | OK061069 | Du et al. (2021) |
| *Brunneofusispora inclinatiostiola* | GZCC 21-0185 | MZ964876 | | MZ964885 | | MZ964867 | | OK061076 | | OK061070 | Du et al. (2021) |
| *Brunneofusispora sennae-torae* | BRIP 72515d | OK493235 | | – | | OK493236 | | – | | – | Wei et al. (2024) |
| *Brunneofusispora sinensis* | KUMCC 17-0030 | MH393557 | | MH393556 | | MH393558 | | – | | MH395329 | Wanasinghe et al. (2020) |
| *Brunneofusispora sinensis* | MFLUCC 20-0016 | MT159624 | | MT159636 | | MT159630 | | MT159613 | | MT159607 | Wanasinghe et al. (2020) |
| *Brunneofusispora* sp*.* | X135 | – | | – | | MK304223 | | – | | – | Calabon et al. (2021) |
| *Neooccultibambusa chiangraiensis* | MFLUCC 12-0559 | KU764699 | | KU712458 | | KU712442 | | – | | KU872761 | Doilom et al. (2017) |
| ***Neooccultibambusa coffeae*** | **KUNCC24-18351** | **OR428416** | | **OR428374** | | **OR428394** | | **OR515518** | | **OR509748** | **This study** |
| ***Neooccultibambusa coffeae*** | **KUNCC24-18353** | **OR428415** | | **OR428373** | | **OR428393** | | **OR515517** | | **OR509747** | **This study** |
| *Neooccultibambusa jonesii* | MFLUCC 16-0643 | KY111437 | | KY111438 | | – | | – | | – | Jayasiri et al. (2016) |
| *Neooccultibambusa kaiyangensis* | CGMCC 3.20404 | MZ964877 | | MZ964886 | | MZ964868 | | OK061077 | | OK061071 | Du et al. (2021) |
| *Neooccultibambusa kaiyangensis* | GZCC 21-0184 | MZ964878 | | MZ964887 | | MZ964869 | | OK061078 | | OK061072 | Du et al. (2021) |
| *Neooccultibambusa pandanicola* | KUMCC 17-0179 | MG298940 | | MG298942 | | MG298941 | | MG298944 | | MG298943 | Hyde et al. (2018) |
| *Neooccultibambusa thailandensis* | MFLUCC 16-0274 | MH260308 | | MH260348 | | MH275074 | | MH412758 | | MH412780 | Tibpromma et al. (2018) |
| *Neooccultibambusa trachycarpi* | CGMCC 3.20405 | MZ964879 | | MZ964888 | | MZ964870 | | OK061079 | | OK061073 | Du et al. (2021) |
| *Neooccultibambusa trachycarpi* | GZCC 21-0181 | MZ964880 | | MZ964889 | | MZ964871 | | OK061080 | | OK061074 | Du et al. (2021) |
| *Neoroussoella clematidis* | MFLUCC 17-2061 | MT214587 | | MT226700 | | MT310632 | | MT394701 | | MT394645 | Phukhamsakda et al. (2020) |
| *Neoroussoella fulvicomae* | MFLUCC 17-2073 | MT214588 | | MT226701 | | MT310633 | | MT394702 | | MT394646 | Phukhamsakda et al. (2020) |
| *Occultibambusa aquatica* | MFLUCC 11-0006 | KX698110 | | KX698112 | | KX698114 | | – | | – | Hyde et al. (2016) |
| *Occultibambusa bambusae* | MFLUCC 13-0855 | KU863112 | | KU872116 | | KU940123 | | KU940170 | | KU940193 | Dai et al. (2017) |
| *Occultibambusa chiangraiensis* | MFLUCC 16-0380 | KX655546 | | KX655551 | | – | | KX655566 | | KX655561 | Hyde et al. (2016) |
| *Occultibambusa fusispora* | MFLUCC 11-0127 | KU863114 | | – | | KU940125 | | KU940172 | | KU940195 | Dai et al. (2017) |
| *Occultibambusa jonesii* | GZCC 16-0117 | KY628322 | | KY628324 | | – | | KY814758 | | KY814756 | Xu et al. (2022) |
| *Occultibambusa kunmingensis* | HKAS 102151 | MN913733 | | MT864342 | | MT627716 | | MT878453 | | MT954407 | Dong et al. (2020) |
| *Occultibambusa maolanensis* | GZCC 16-0116 | KY628323 | | KY628325 | | – | | KY814759 | | KY814757 | Zhang et ai. (2017) |
| *Occultibambusa pustula* | MFLUCC 11-0502 | KU863115 | | KU872118 | | KU940126 | | – | | – | Dong et al. (2020) |
| *Ohleria modesta* | CBS 141480 | KX650563 | | KX650513 | | KX650563 | | KX650583 | | KX650534 | Dai et al. (2017) |
| *Ohleria modesta* | MGC | KX650562 | | – | | KX650562 | | KX650582 | | KX650533 | Dai et al. (2017) |
| *Roussoella nitidula* | MFLUCC 11-0182 | KJ474843 | | – | | KJ474835 | | KJ474859 | | KJ474852 | Liu et al. (2014) |
| *Roussoella thailandica* | MFLUCC 11-0621 | KJ474846 | | – | | KJ474838 | | – | | – | Liu et al. (2014) |
| *Seriascoma didymosporum* | MFLUCC 11-0179 | KU863116 | | KU872119 | | KU940127 | | KU940173 | | KU940196 | Dai et al. (2017) |
| *Seriascoma yunnanense* | MFLU 19-0690 | MN174695 | | MN174694 | | – | | MN210324 | | MN381858 | Rathnayaka et al. (2019) |
| *Thyridaria acaciae* | CBS 138873 | KP004497 | | – | | KP004469 | | – | | – | Crous et al. (2014) |
| *Thyridaria broussonetiae* | CBS 141481 | KX650568 | | KX650515 | | KX650568 | | KX650586 | | KX650539 | Jaklitsch et al. (2016) |
| *Thyridaria broussonetiae* | TB | KX650567 | | – | | KX650567 | | KX650585 | | KX650538 | Jaklitsch et al. (2016) |
| *Thyridaria broussonetiae* | TB2 | KX650570 | | – | | KX650570 | | KX650587 | | KX650540 | Jaklitsch et al. (2016) |
| *Versicolorisporium triseptatum* | JCM 14775 | AB330081 | | AB524501 | | AB365596 | | – | | – | Hatakeyama et al. (2008) |

Newly generated sequences are in bold, and “–” indicates the sequence unavailability.

**Supplementary Table 12**. Names, voucher numbers, and corresponding GenBank numbers of the taxa used in the phylogenetic analyses of *Pararoussoella*.

| Species | Voucher number | GenBank accession number | | | | | References |
| --- | --- | --- | --- | --- | --- | --- | --- |
|  |  | ITS | LSU | SSU | *RPB*2 | *TEF*1-α |  |
| *Arthopyrenia* sp. | UTHSC DI16-362 | LT796905 | LN907505 | – | LT797065 | LT797145 | Valenzuela-Lopez et al. (2017) |
| *Arthopyrenia* sp. | UTHSC DI16-334 | LT796887 | LN907477 | – | – | LT797127 | Valenzuela-Lopez et al. (2017) |
| *Neoroussoella alishanense* | FU31016 | MK503816 | MK503822 | MK503828 | MN037756 | MK336181 | Karunarathna et al. (2019) |
| *Neoroussoella bambusae* | MFLUCC 11-0124 | KJ474827 | KJ474839 | – | KJ474856 | KJ474848 | Liu et al. (2014) |
| *Neoroussoella entadae* | MFLUCC 15-0098 | MH275075 | MH260309 | MH260349 | – | – | Tibpromma et al. (2018) |
| *Neoroussoella heveae* | MFLUCC 17-0338 | MH590693 | MH590689 | MH590691 | – | – | Phookamsak et al. (2019) |
| *Neoroussoella lenispora* | GZCC 16-0020 | – | KX791431 | – | – | – | Hyde et al. (2016) |
| *Neoroussoella leucaenae* | MFLUCC 18-1544 | MK347767 | MK347984 | MK347874 | MK434876 | MK360067 | Jayasiri et al. (2019) |
| *Neoroussoella solani* | CPC 26331 | KX228261 | KX228312 | – | – | – | Crous et al. (2016) |
| ***Pararoussoella coffeae*** | **KUNCC24-18355** | **OR428401** | **OR428423** | **OR428381** | **OR515521** | **OR509755** | This study |
| ***Pararoussoella coffeae*** | **KUNCC24-18356** | **OR428402** | **OR428424** | **OR428382** | **OR515522** | **OR509756** | This study |
| *Pararoussoella mangrovei* | MFLUCC 16-0424 | MH025951 | MH023318 | – | MH028250 | MH028246 | Hyde et al. (2018) |
| *Pararoussoella mukdahanensis* | MFLUCC 11-0201 | KU940129 | KU863118 | KU872121 | – | – | Dai et al. (2017) |
| *Pararoussoella rosarum* | MFLUCC 17-0796 | NR_157529 | NG_059872 | MG829154 | – | MG829224 | Wanasinghe et al. (2018) |
| *Pararoussoella quercina* | CPC 34864 | MT223828 | MT223920 | – | – | – | Crous et al. (2020) |
| *Pararoussoella juglandicola* | CBS 145037 | MK442607 | MK442543 | – | MK442671 | MK442699 | Crous et al. (2019) |
| *Parathyridaria percutanea* | CBS 128203 | KF322117 | KF366448 | KF366450 | KF366453 | KF407988 | Ahmed et al. (2014b) |
| *Parathyridaria percutanea* | CBS 868.95 | KF322118 | KF366449 | KF366451 | KF366452 | KF407987 | Ahmed et al. (2014b) |
| *Parathyridaria ramulicola* | CBS 141479 | KX650565 | KX650565 | KX650514 | KX650584 | KX650536 | Jaklitsch et al. (2016) |
| *Parathyridaria ramulicola* | MF4 | KX650564 | KX650564 | – | – | KX650535 | Jaklitsch et al. (2016) |
| *Parathyridaria robiniae* | MFLUCC 14-1119 | KY511142 | KY511141 | – | – | KY549682 | Tibpromma et al. (2017) |
| *Pseudoneoconiothyrium euonymi* | CBS 143426 | MH107915 | MH107961 | – | MH108007 | – | Crous et al. (2018c) |
| *Pseudoneoconiothyrium rosae* | MFLUCC 15-0052 | NR_157523 | NG_059868 | NG_063686 | MN814846 | MN794051 | Wanasinghe et al. (2018) |
| *Pseudoroussoella chromolaenae* | MFLUCC 17-1492 | MT214345 | MT214439 | MT214393 | – | MT235769 | Mapook et al. (2020) |
| *Pseudoroussoella elaeicola* | MFLUCC 17-1483 | MT214348 | MT214442 | MT214396 | MT235808 | MT235772 | Mapook et al. (2020) |
| *Pseudoroussoella elaeicola* | MFLUCC 15-0276a | MH742329 | MH742326 | – | – | – | Phookamsak et al. (2019) |
| *Pseudoroussoella elaeicola* | MFLUCC 15-0276b | MH742330 | MH742327 | – | – | – | Phookamsak et al. (2019) |
| *Roussoella aquatica* | MFLUCC 18-1040 | NR_171975 | NG_073797 | NG_073545 | – | – | Dong et al. (2020) |
| *Roussoella arundinacea* | CBS 146088 | MT223838 | MT223928 | – | MT223699 | MT223723 | Crous et al. (2020) |
| *Roussoella chiangraina* | MFLUCC 10-0556 | KJ474828 | KJ474840 | – | KJ474857 | KJ474849 | Liu et al. (2014) |
| *Roussoella doimaesalongensis* | MFLUCC 14-0584 | KY026584 | KY000659 | – | KY678394 | KY651249 | Thambugala et al. (2017) |
| *Roussoella guttulata* | MFLUCC 20-0102 | NR_172428 | NG_075383 | – | MW022187 | MW022188 | Zhang et al. (2020) |
| *Roussoella hysterioides* | CBS 546.94 | KF443405 | KF443381 | AY642528 | KF443392 | KF443399 | Ahmed et al. (2014b) |
| *Roussoella intermedia* | CBS 170.96 | KF443407 | KF443382 | KF443390 | KF443394 | KF443398 | Ahmed et al. (2014) |
| *Roussoella japanensis* | MAFF 239636 | KJ474829 | AB524621 | AB524480 | AB539101 | AB539114 | Liu et al. (2014) |
| *Roussoella kunmingensis* | KUMCC 18-0128 | MH453491 | MH453487 | – | MH453484 | MH453480 | Jiang et al. (2019) |
| *Roussoella magnatum* | MFLUCC 15-0185 | – | KT281980 | – | – | – | Ariyawansa et al. (2015)  al. (2015) |
| *Roussoella margidorensis* | MUT 5329 | KU314944 | MN556322 | MN556309 | MN605917 | MN605897 | Poli et al. (2020) |
| *Roussoella mediterranea* | MUT 5369 | KU314947 | MN556324 | MN556310 | MN605919 | MN605899 | Poli et al. (2020) |
| *Roussoella mexicana* | CPC 25355 | KT950848 | KT950862 | – | – | – | Crous et al. (2015)  (2015) |
| *Roussoella multiloculate* | GMBCC1056 | OM891799 | OM884015 | OM892821 | ON098369 | ON098343 | Dai et al. (2022) |
| *Roussoella neopustulans* | MFLUCC 11-0609 | KJ474833 | KJ474841 | – | – | KJ474850 | Liu et al. (2014) |
| *Roussoella nitidula* | MFLUCC 11-0182 | KJ474835 | KJ474843 | – | KJ474859 | KJ474852 | Liu et al. (2014) |
| *Roussoella padinae* | MUT 5503 | KU158170 | MN556327 | MN556312 | MN605922 | MN605902 | Dai et al. (2022) |
| *Roussoella papillate* | GMBCC1121 | OM891814 | OM755608 | – | ON098378 | ON098346 | Dai et al. (2022) |
| *Roussoella pseudohysterioides* | MFLUCC 13-0852 | KU940131 | KU863120 | KU872123 | – | KU940198 | Jiang et al. (2019) |
| *Roussoella pustulans* | MAFF 239637 | KJ474830 | AB524623 | AB524482 | AB539103 | AB539116 | Liu et al. (2014) |
| *Roussoella scabrispora* | MFLUCC 11-0624 | KJ474836 | KJ474844 | – | KJ474860 | KJ474853 | Liu et al. (2014) |
| *Roussoella siamensis* | MFLUCC 11-0149 | KJ474837 | KJ474845 | KU872125 | KJ474861 | KJ474854 | Liu et al. (2014) |
| *Roussoella sinensis* | GMBCC1119 | OM891813 | OM884024 | ON228185 | ON098379 | ON098357 | Dai et al. (2022) |
| *Roussoella thailandica* | MFLUCC 11-0621 | KJ474838 | KJ474846 | – | – | – | Liu et al. (2014) |
| *Roussoella tuberculata* | MFLUCC 13-0854 | KU940132 | KU863121 | KU872124 | – | KU940199 | Dai et al. (2017) |
| *Roussoella uniloculata* | GMBCC1110 | OM891809 | OM801286 | OM891829 | ON098374 | ON098360 | Dai et al. (2022) |
| *Roussoella verrucispora* | CBS 125434 | KJ474832 | AB524622 | AB524481 | AB539102 | AB539115 | Liu et al. (2014) |
| *Roussoella yunnanensis* | KUMCC 18-0115 | MH453492 | MH453488 | – | – | MH453481 | Jiang et al. (2019) |
| *Roussoellopsis macrospora* | MFLUCC 12-0005 | KJ739604 | KJ474847 | KJ739608 | KJ474862 | KJ474855 | Liu et al. (2014) |
| *Roussoellopsis* sp. | KT 1710 | – | AB524626 | AB524485 | – | – | Tanaka et al. (2009) |
| *Roussoellopsis tosaensis* | KT 1659 | – | AB524625 | AB524484 | AB539104 | MG829199 | Tanaka et al. (2009) |
| *Setoarthopyrenia chromolaenae* | MFLUCC 17-1444 | MT214344 | MT214438 | MT214392 | MT235805 | MT235768 | Mapook et al. (2020) |
| *Thyridaria acaciae* | CBS 138873 | KP004469 | KP004497 | – | – | – | Crous et al. (2014) |
| *Thyridaria broussonetiae* | CBS 141481 | NR_147658 | KX650568 | KX650515 | KX650586 | KX650539 | Jaklitsch et al. (2016) |
| *Torula herbarum* | CBS 111855 | KF443409 | KF443386 | KF443391 | KF443396 | KF443403 | Ahmed et al. (2014b) |
| *Torula hollandica* | CBS 220.69 | KF443406 | KF443384 | KF443389 | KF443393 | KF443401 | Crous et al. (2015) |
| *Xenoroussoella triseptata* | MFLUCC 17-1438 | MT214343 | MT214437 | MT214391 | MT235804 | MT235767 | Mapook et al. (2020) |

Newly generated sequences are in bold, “–” indicates the sequence unavailability.

**Supplementary Table 13**. Names, voucher numbers, and corresponding GenBank numbers of the taxa used in the phylogenetic analyses of *Cycasicola*.

| Species | Voucher number | GenBank accession number | | | | | | | References |
| --- | --- | --- | --- | --- | --- | --- | --- | --- | --- |
|  |  | ITS | LSU | | | SSU | *TEF*1-α | |  |
| *Arthopyrenia salicis* | CBS 368.94 | KF443410 | | AY538339 | AY538333 | | | KF443404 | Ahmed et al. (2014a) |
| *Chromolaenomyces appendiculatus* | MFLUCC 17-1455 | NR_168862 | | NG_068705 | MT214394 | | | MT235770 | Mapook et al. (2020) |
| ***Cycasicola coffeae*** | **KUNCC24-18357** | **OR428409** | | **OR428431** | **OR428387** | | | **OR509761** | **This study** |
| ***Cycasicola coffeae*** | **KUNCC24-18358** | **OR428410** | | **OR428432** | **OR428388** | | | **OR509762** | **This study** |
| *Cycasicola goaensis* | MFLUCC 17-0754 | MG828885 | | MG829001 | MG829112 | | | MG829198 | Wanasinghe et al. (2018) |
| *Cycasicola leucaenae* | MFLUCC 17-0914 | MK347726 | | MK347942 | MK347833 | | | MK360046 | Jayasiri et al. (2019) |
| *Liua muriformis* | KUMCC 18-0177 | MK433599 | | MK433598 | MK433595 | | | MK426798 | Phookamsak et al. (2020) |
| *Neoroussoella alishanense* | AKW 11 FU31018 | MK503818 | | MK503824 | MK503830 | | | MK336182 | Karunarathna et al. (2019) |
| *Neoroussoella alishanense* | AKW 03 FU31016 | MK503816 | | MK503822 | MK503828 | | | MK336181 | Karunarathna et al. (2019) |
| *Neoroussoella entadae* | MFLUCC 18-0243 | MK347786 | | MK348004 | MK347893 | | | MK360065 | Jayasiri et al. (2019) |
| *Neoroussoella leucaenae* | MFLUCC 18-1544 | MK347767 | | MK347984 | MK347874 | | | MK360067 | Jayasiri et al. (2019) |
| *Occultibambusa bambusae* | MFLUCC 11-0394 | KU940124 | | KU863113 | – | | | KU940194 | Dai et al. (2016) |
| *Occultibambusa bambusae* | MFLUCC 13-0855 | KU940123 | | KU863112 | KU872116 | | | KU940193 | Dai et al. (2016) |
| *Ohleria modesta* | MGC | KX650562 | | KX650562 | – | | | KX650533 | Jaklitsch and Voglmayr (2016) |
| *Ohleria modesta* | OM | KX650563 | | KX650563 | KX650513 | | | KX650534 | Jaklitsch and Voglmayr (2016) |
| *Parathyridaria clematidis* | MFLUCC 17-2154 | MT310645 | | MT214601 | MT226712 | | | MT394657 | Phukhamsakda et al. (2020) |
| *Parathyridaria clematidis* | MFLUCC 17-2157 | MT310644 | | MT214600 | MT226711 | | | MT394656 | Phukhamsakda et al. (2020) |
| *Parathyridaria clematidis* | MFLUCC 17-2160 | MT310643 | | MT214599 | MT226710 | | | MT394655 | Phukhamsakda et al. (2020) |
| *Parathyridaria clematidis* | MFLUCC 17-2185 | MT310642 | | MT214598 | NG_070668 | | | MT394654 | Phukhamsakda et al. (2020) |
| *Parathyridaria ellipsoidea* | KNU-JJ-1829 | LC552950 | | LC552952 | – | | | – | Das et al. (2021) |
| *Parathyridaria flabelliae* | MUT 4886 | KR014358 | | KP671720 | KT587317 | | | MN605910 | Poli et al. (2020) |
| *Parathyridaria flabelliae* | MUT 4859 | KR014355 | | KP671716 | KT587315 | | | MN605909 | Poli et al. (2020) |
| *Parathyridaria percutanea* | CBS 128203 | KF322117 | | KF366448 | KF366450 | | | KF407988 | Ahmed et al. (2014a) |
| *Parathyridaria percutanea* | CBS 868.95 | KF322118 | | KF366449 | KF366451 | | | KF407987 | Ahmed et al. (2014a) |
| *Parathyridaria philadelphi* | CBS 143432 | MH107905 | | NG_063958 | – | | | MH108023 | Crous et al. (2018a) |
| *Parathyridaria ramulicola* | MUT 4397 | KC339235 | | KF636775 | MN556311 | | | MN605913 | Panno et al. (2013) |
| *Parathyridaria ramulicola* | CBS 141479 | NR_147657 | | KX650565 | KX650514 | | | KX650536 | Jaklitsch and Voglmayr (2016) |
| *Parathyridaria robiniae* | MUT 2452 | MG813183 | | MG816491 | MN556312 | | | MN605903 | Bovio et al. (2018) |
| *Parathyridaria robiniae* | MUT 4893 | KM355998 | | MN556328 | KM355993 | | | MN605904 | Bovio et al. (2018) |
| *Parathyridaria robiniae* | MFLUCC 14-1119 | KY511142 | | KY511141 | – | | | KY549682 | Tibpromma et al. (2017) |
| *Parathyridaria rosae* | MFLU 17-0623 | NR_157530 | | NG_059873 | – | | | – | Wanasinghe et al. (2018) |
| *Parathyridaria serratifoliae* | MFLUCC 17-2210 | MT310646 | | MT214602 | NG_070669 | | | MT394658 | Phukhamsakda et al. (2020) |
| *Parathyridaria tyrrhenica* | MUT 4966 | KR014366 | | KP671740 | KT587309 | | | MN605911 | Poli et al. (2020) |
| *Parathyridaria tyrrhenica* | MUT 5371 | KU314951 | | MN556329 | KU314952 | | | MN605912 | Poli et al. (2020) |
| *Parathyridaria virginianae* | MFLUCC 17-2163 | MT310647 | | NG_073853 | NG_070670 | | | MT394659 | Phukhamsakda et al. (2020) |
| *Parathyridariella dematiacea* | MUT 4419 | KC339245 | | KF636786 | MN556313 | | | MN605905 | Poli et al. (2020) |
| *Parathyridariella dematiacea* | MUT 5310 | KU255057 | | MN556330 | MN556314 | | | MN605907 | Poli et al. (2020) |
| *Parathyridariella dematiacea* | MUT 5381 | KU314959 | | MN556331 | KU314960 | | | MN605908 | Poli et al. (2020) |
| *Parathyridariella dematiacea* | MUT 4884 | MN556317 | | KP671726 | KT587329 | | | MN605906 | Poli et al. (2020) |
| *Pseudothyridariella chromolaenae* | MFLUCC 17-1472 | NR_168863 | | NG_068706 | MT214395 | | | MT235771 | Mapook et al. (2020) |
| *Pseudothyridariella mahakoshae* | NFCCI 4215 | MG020435 | | MG020438 | MG020441 | | | MG023140 | Devadatha et al. (2018) |
| *Roussoella hysterioides* | CBS 546.94 | KF443405 | | KF443381 | AY642528 | | | KF443399 | Ahmed et al. (2014a) |
| *Roussoella intermedia* | CBS 170.96 | KF443407 | | KF443382 | KF443390 | | | KF443398 | Ahmed et al. (2014a) |
| *Roussoella japanensis* | MAFF 239636 | KJ474829 | | AB524621 | AB524480 | | | AB539114 | Liu et al. (2014) |
| *Roussoella margidorensis* | MUT 5329 | KU314944 | | MN556322 | MN556309 | | | MN605897 | Poli et al. (2020) |
| *Roussoella mediterranea* | MUT 5306 | KU255054 | | MN556323 | MN556310 | | | MN605898 | Poli et al. (2020) |
| *Roussoella padinae* | MUT 5341 | KU158153 | | MN556325 | KU158176 | | | MN605900 | Poli et al. (2020) |
| *Roussoella pustulans* | KT 1709 | KJ474830 | | AB524623 | AB524482 | | | AB539116 | Liu et al. (2014) |
| *Roussoella siamensis* | MFLUCC 11-0149 | KJ474837 | | KJ474845 | KU872125 | | | KJ474854 | Liu et al. (2014) |
| *Thyridaria acaciae* | CBS 138873 | KP004469 | | KP004497 | – | | | – | Crous et al. (2014) |
| *Thyridaria aureobrunnea* | MFLUCC 21-0090 | MZ538528 | | MZ538562 | – | | | – | Boonmee et al. (2021) |
| *Thyridaria broussonetiae* | TB | KX650567 | | KX650567 | – | | | KX650538 | Jaklitsch and Voglmayr (2016) |
| *Thyridaria broussonetiae* | TB1a | KX650569 | | KX650569 | – | | | – | Jaklitsch and Voglmayr (2016) |
| *Thyridaria broussonetiae* | TB2 | KX650570 | | KX650570 | – | | | KX650540 | Jaklitsch and Voglmayr (2016) |
| *Thyridaria broussonetiae* | TB1 | KX650568 | | KX650568 | KX650515 | | | KX650539 | Jaklitsch and Voglmayr (2016) |
| *Thyridaria jonahhulmei* | KUMCC 21-0816 | ON007041 | | ON007037 | ON007046 | | | ON009131 | Wanasinghe and Mortimer (2022) |
| *Thyridaria jonahhulmei* | KUMCC 21-0817 | ON007042 | | ON007038 | ON007047 | | | ON009132 | Wanasinghe and Mortimer (2022) |
| *Thyridariella mangrovei* | NFCCI 4214 | MG020436 | | MG020439 | MG020442 | | | MG020444 | Devadatha et al. (2018) |
| *Thyridariella mangrovei* | NFCCI 4213 | MG020434 | | MG020437 | MG020440 | | | MG020443 | Devadatha et al. (2018) |
| *Torula herbarum* | CBS 111855 | KF443409 | | KF443386 | KF443391 | | | KF443403 | Ahmed et al. (2014a) |
| *Torula herbarum* | CBS 595.96 | KF443408 | | KF443385 | KF443387 | | | KF443402 | Ahmed et al. (2014a) |

Newly generated sequences are in bold, “–” indicates the sequence unavailability.

**Supplementary Table 14**. Phylogenetic statistics in each tree legend.

| Figure number | Total characters | Final likelihood value | Distinct alignment patterns | Undetermined characters or gaps | Gamma distribution (α) | Estimated base frequencies | | | | | | | | | |
| --- | --- | --- | --- | --- | --- | --- | --- | --- | --- | --- | --- | --- | --- | --- | --- |
|  |  |  |  |  |  | A | C | G | T | AC | AG | AT | CG | CT | GT |
| **3** | 4654 | - 20864.078247 | 1297 | 25.51% | 0.174417 | 0.244074 | 0.255051 | 0.269570 | 0.231306 | 1.349807 | 3.164833 | 1.472521 | 1.013251 | 7.310405 | 1.000000 |
| **5** | 3340 | - 16527.715728 | 1034 | 22.54% | 0.178820 | 0.238817 | 0.255490 | 0.272013 | 0.233680 | 1.425290 | 2.332338 | 1.535354 | 1.159643 | 6.651510 | 1.000000 |
| **7** | 2246 | -7353.044296 | 403 | 11.67% | 0.162600 | 0.239449 | 0.240190 | 0.276733 | 0.243629 | 2.527717 | 13.099659 | 3.639751 | 1.629076 | 24.442787 | 1.000000 |
| **9** | 4325 | - 23104.441626 | 1528 | 22.25% | 0.168772 | 0.248393 | 0.248745 | 0.267301 | 0.235561 | 1.339800 | 3.748512 | 1.387971 | 1.287352 | 7.888021 | 1.000000 |
| **11** | 3371 | - 24543.565116 | 1615 | 21.88% | 0.299921 | 0.248235 | 0.238371 | 0.269117 | 0.244277 | 1.091908 | 2.424931 | 1.582888 | 0.877623 | 6.533597 | 1.000000 |
| **13** | 4404 | - 24716.386641 | 1414 | 29.80% | 0.175143 | 0.241570 | 0.252231 | 0.270677 | 0.235522 | 1.764679 | 4.646256 | 1.689674 | 0.923731 | 8.272955 | 1.000000 |
| **15** | 4434 | - 29981.179416 | 1810 | 37.21% | 0.203853 | 0.253137 | 0.234062 | 0.271290 | 0.241511 | 1.755919 | 4.590368 | 1.428862 | 1.416373 | 8.476162 | 1.000000 |
| **17** | 4387 | - 24509.308516 | 1520 | 24.21% | 0.179191 | 0.246633 | 0.253860 | 0.271381 | 0.228125 | 2.071171 | 4.905156 | 1.731671 | 1.649762 | 10.215993 | 1.000000 |
| **19** | 4294 | - 36528.895756 | 1924 | 32.94% | 0.243128 | 0.247636 | 0.253433 | 0.269644 | 0.229288 | 1.450292 | 3.863454 | 1.529977 | 1.070850 | 7.278891 | 1.000000 |
| **21** | 3331 | - 18803.214047 | 1205 | 15.70% | 0.207937 | 0.245002 | 0.258591 | 0.268797 | 0.227610 | 1.419228 | 2.263816 | 1.566788 | 0.933159 | 6.360775 | 1.000000 |

**References**

Ahmadpour SA, Mehrabi-Koushki M, Farokhinejad R, Asgari B (2022a) New species of the family *Didymellaceae* in Iran. Mycological Progress 21(2): 28. <https://doi.org/10.1007/s11557-022-01800-5>

Ahmadpour SA, Mehrabi-Koushki M, Farokhinejad R, Asgari B (2022b) *Xenodidymella iranica* sp. nov. and new hosts of *X. glycyrrhizicola* in Iran. Tropical Plant Pathology 47(3): 430–441. <https://doi.org/10.1007/s40858-022-00491-3>

Ahmed SA, Van De Sande WWJ, Stevens DA, Fahal A, Van Diepeningen AD, Menken SBJ, De Hoog GS (2014a) Revision of agents of black-grain eumycetoma in the order *Pleosporales*. Persoonia-Molecular Phylogeny and Evolution of Fungi 33(1): 141–154. <https://doi.org/10.3767/003158514X684744>

Ahmed SA, Stevens DA, van de Sande WW, Meis JF, De Hoog GS (2014b) *Roussoella percutanea*, a novel opportunistic pathogen causing subcutaneous mycoses. Medical Mycology 52: 689–698. https://doi.org/10.1093/mmy/myu035

Alidadi A, Kowsari M, Javan-Nikkhah M, Jouzani GS, Rastaghi ME (2019) New pathogenic and endophytic fungal species associated with Persian oak in Iran. European Journal of Plant Pathology 155: 1017–1032. <https://doi.org/10.1007/s10658-019-01830-y>

Aveskamp MM, de Gruyter J, Woudenberg JHC, Verkley GJM, Crous PW (2010) Highlights of the *Didymellaceae*: a polyphasic approach to characterise *Phoma* and related pleosporalean genera. Studies in Mycology 65(1): 1–60. <https://doi.org/10.3114/sim.2010.65.01>

Ariyawansa HA, Maharachchikumbura SS, Karunarathne SC, Chukeatirote E, Bahkali AH, Kang JK, Hyde KD (2013) *Deniquelata barringtoniae* gen. et sp. nov., associated with leaf spots of *Barringtonia asiatica*. Phytotaxa 105(1): 11–20. <https://doi.org/10.11646/phytotaxa.105.1.2>

Ariyawansa HA, Hyde KD, Jayasiri SC, Buyck B, Chethana KWT, Dai DQ, Dai YC, Daranagama DA, Jayawardena RS, Lücking R, Ghobad-Nejhad M, Niskanen T, Thambugala KM, Voigt K, Zhao RL, Li GJ, Doilom M, Boonmee S, Yang ZL, Cai Q, Cui YY, Bahkali AH, Chen J, Cui BK, Chen JJ, Dayarathne MC, Dissanayake AJ, Ekanayaka AH, Hashimoto A, Hongsanan S, Jones EBG, Larsson E, Li WJ, Li QR, Liu JK, Luo ZL, Maharachchikumbura SSN, Mapook A, McKenzie EHC, Norphanphoun C, Konta S, Pang KL, Perera RH, Phookamsak R, Phukhamsakda C, Pinruan U, Randrianjohany E, Singtripop C, Tanaka K, Tian CM, Tibpromma S, Abdel-Wahab MA, Wanasinghe DN, Wijayawardene NN, Zhang JF, Zhang H, Abdel-Aziz FA, Wedin M, Westberg M, Ammirati JF, Bulgakov TS, Lima DX, Callaghan TM, Callac P, Chang CH, Coca LF, Dal-Forno M, Dollhofer V, Fliegerová K, Greiner K, Griffith GW, Ho HM, Hofstetter V, Jeewon R, Kang JC, Wen TC, Kirk PM, Kytövuori I, Lawrey JD, Xing J, Li H, Liu ZY, Liu XZ, Liimatainen K, Lumbsch HT, Matsumura M, Moncada B, Nuankaew S, Parnmen S, de Azevedo Santiago ALCM, Sommai S, Song Y, de Souza CAF, de Souza-Motta CM, Su HY, Suetrong S, Wang Y, Wei SF, Wen TC, Yuan HS, Zhou LW, Réblová M, Fournier J, Camporesi E, Luangsa-ard JJ, Tasanathai K, Khonsanit A, Thanakitpipattana D, Somrithipol S, Diederich P, Millanes AM, Common RS, Stadler M, Yan JY, Li XH, Lee HW, Nguyen TTT, Lee HB, Battistin E, Marsico O, Vizzini A, Vila J, Ercole E, Eberhardt U, Simonini G, Wen HA, Chen XH, Miettinen O, Spirin V, Hernawati (2014) A molecular phylogenetic reappraisal of the *Didymosphaeriaceae* (= *Montagnulaceae*). Fungal Diversity 68: 69–104. <https://doi.org/10.1007/s13225-014-0305-6>

Ariyawansa HA, Hyde KD, Jayasiri SC, Buyck B, Chethana KWT, Dai DQ, Dai YC, Daranagama DA, Jayawardena RS, Lücking R, Ghobad-Nejhad M, Niskanen T, Thambugala KM, Voigt K, Zhao RL, Li GJ, Doilom M, Boonmee S, Yang ZL, Cai Q, Cui YY, Bahkali AH, Chen J, Cui BK, Chen JJ, Dayarathne MC, Dissanayake AJ, Ekanayaka AH, Hashimoto A, Hongsanan S, Jones EBG, Larsson E, Li WJ, Li QR, Liu JK, Luo ZL, Maharachchikumbura SSN, Mapook A, McKenzie EHC, Norphanphoun C, Konta S, Pang KL, Perera RH, Phookamsak R, Phukhamsakda C, Pinruan U, Randrianjohany E, Singtripop C, Tanaka K, Tian CM, Tibpromma S, Abdel-Wahab MA, Wanasinghe DN, Wijayawardene NN, Zhang JF, Zhang H, Abdel-Aziz FA, Wedin M, Westberg M, Ammirati JF, Bulgakov TS, Lima DX, Callaghan TM, Callac P, Chang CH, Coca LF, Dal-Forno M, Dollhofer V, Fliegerová K, Greiner K, Griffith GW, Ho HM, Hofstetter V, Jeewon R, Kang JC, Wen TC, Kirk PM, Kytövuori I, Lawrey JD, Xing J, Li H, Liu ZY, Liu XZ, Liimatainen K, Lumbsch HT, Matsumura M, Moncada B, Nuankaew S, Parnmen S, de Azevedo Santiago ALCM, Sommai S, Song Y, de Souza CAF, de Souza-Motta CM, Su HY, Suetrong S, Wang Y, Wei SF, Wen TC, Yuan HS, Zhou LW, Réblová M, Fournier J, Camporesi E, Luangsa-ard JJ, Tasanathai K, Khonsanit A, Thanakitpipattana D, Somrithipol S, Diederich P, Millanes AM, Common RS, Stadler M, Yan JY, Li XH, Lee HW, Nguyen TTT, Lee HB, Battistin E, Marsico O, Vizzini A, Vila J, Ercole E, Eberhardt U, Simonini G, Wen HA, Chen XH, Miettinen O, Spirin V, Hernawati (2015) Fungal diversity notes 111–252—taxonomic and phylogenetic contributions to fungal taxa. Fungal Diversity 75: 27–274. https://doi.org/10.1007/s13225-015-0346-5

Ariyawansa HA, Jaklitsch WM, Voglmayr H (2018) Additions to Taiwan fungal flora 1: *Neomassariaceae* fam. nov. Cryptogamie Mycologie 39: 359–372. https://doi.org/10.7872/crym/v39.iss3.2018.359

Ariyawansa HA, Tsai I, Thambugala KM, Chuang WY, Lin SR, Hozzein WN, Cheewangkoon R (2020a) Species diversity of *Pleosporalean* taxa associated with *Camellia sinensis* (L.) Kuntze in Taiwan. Scientific Reports 10(1): 12762. <https://doi.org/10.1038/s41598-020-69718-0>

Ariyawansa HA, Tsai I, Hozzein WN, Thambugala KM (2020b) *Leucaenicola osmanthi* sp. nov. (*Bambusicolaceae, Pleosporales*), causing leaf spot of *Osmanthus fragrans* in Taiwan. Phytotaxa 437(1): 3. https://doi.org/10.11646/phytotaxa.437.1.3

Aptroot A (1995) Redisposition of some species excluded from *Didymosphaeria* (*Ascomycotina*). Nova Hedwigia 60: 325–379.

Bhat D, Sutton B (1985) New and interesting hyphomycetes from Ethiopia. Transactions of the British Mycological Society 85: 107–122. https://doi.org/10.1016/S0007-1536(85)80160-1

Bhat DJ (2008) The forests of Western Ghats, an abode of novel and interesting microfungi. Kavaka 36: 1–11.

Boonmee S, Wanasinghe DN, Calabon MS, Huanraluek N, Chandrasiri SKU, Jones GEB, Rossi W, Leonardi M, Singh SK, Rana S, Singh PN, Maurya DK, Lagashetti AC, Choudhary D, Dai YC, Zhao CL, Mu YH, Yuan HS, He SH, Phookamsak R, Jiang HB, Martín MP, Dueñas M, Telleria MT, Kałucka IL, Jagodziński AM, Liimatainen K, Pereira DS, Phillips AJL, Suwannarach N, Kumla J, Khuna S, Lumyong S, Potter TB, Shivas RG, Sparks AH, Vaghefi N, Abdel-Wahab MA, Abdel-Aziz FA, Li GJ, Lin WF, Singh U, Bhatt RP, Lee HB, Nguyen TTT, Kirk PM, Dutta AK, Acharya K, Sarma VV, Niranjan M, Rajeshkumar KC, Ashtekar N, Lad S, Wijayawardene NN, Bhat DJ, Xu RJ, Wijesinghe SN, Shen HW, Luo ZL, Zhang JY, Sysouphanthong P, Thongklang N, Bao DF, Aluthmuhandiram JVS, Abdollahzadeh J, Javadi A, Dovana F, Usman M, Khalid AN, Dissanayake AJ, Telagathoti A, Probst M, Peintner U, Garrido-Benavent I, Bóna L, Merényi Z, Boros L, Zoltán B, Stielow JB, Jiang N, Tian CM, Shams E, Dehghanizadeh F, Pordel A, Javan-Nikkhah M, Denchev TT, Denchev CM, Kemler M, Begerow D, Deng CY, Harrower E, Bozorov T, Kholmuradova T, Gafforov Y, Abdurazakov A, Xu JC, Mortimer PE, Ren GC, Jeewon R, Maharachchikumbura SSN, Phukhamsakda C, Mapook A, Hyde KD (2021) Fungal diversity notes 1387–1511: Taxonomic and phylogenetic contributions on genera and species of fungal taxa. Fungal Diversity 111(1): 1–335. <https://doi.org/10.1007/s13225-021-00489-3>

Bovio E, Garzoli L, Poli A, Prigione V, Firsova D, McCormack GP, Varese GC (2018) The culturable mycobiota associated with three Atlantic sponges, including two new species: *Thelebolus balaustiformis* and *T. spongiae*. Fungal Systematics and Evolution 1(1): 141–167. <https://doi.org/10.3114/fuse.2018.01.07>

Bao DF, Su HY, Maharachchikumbura SSN, Liu JK, Nalumpang S, Luo ZL, Hyde KD (2019) Lignicolous freshwater fungi from China and Thailand: Multi-gene phylogeny reveals new species and new records in *Lophiostomataceae*. Mycosphere 10(1): 1080–1099. https://doi.org/10.5943/mycosphere/10/1/20

Borse BD (1987) New species of *Aigialus* from India. Transactions of the British Mycological Society 88: 424–426.

Chen Q, Jiang JR, Zhang GZ, Cai L, Crous PW (2015) Resolving the *Phoma* enigma. Studies in Mycology 82: 137–217. <https://doi.org/10.1016/j.simyco.2015.10.003>

Chen Y, Tian W, Guo Y, Madrid H, Maharachchikumbura SS (2022) *Synhelminthosporium* gen. et sp. nov. and two new species of *Helminthosporium* (*Massarinaceae, Pleosporales*) from Sichuan Province, China. Journal of Fungi 8(7): 712. https://doi.org/10.3390/jof8070712

Crane JL, Shearer CA (1991) A nomenclator of *Leptosphaeria* V. Cesati & G. DeNotaris. Illinois Nat. Hist. Survey, Biol. Notes 34: 1–355. https://doi.org/10.21900/j.inhs.v34.133

Cai L, Hyde KD (2007) *Ascorhombispora aquatica* gen. et sp. nov. from a freshwater habitat in China, and its phylogenetic placement based on molecular data. Cryptogamie Mycologie 28: 291.

Calabon MS, Jones EG, Boonmee S, Doilom M, Lumyong S, Hyde KD (2021) Five novel freshwater ascomycetes indicate high undiscovered diversity in lotic habitats in Thailand. Journal of Fungi 7: 117. <https://doi.org/10.3390/jof7020117>

Crous PW, Slippers B, Wingfield MJ, Rheeder J, Marasas WF, Philips AJ, Groenewald JZ (2006) Phylogenetic lineages in the *Botryosphaeriaceae*. Studies in Mycology 55: 235–253. <https://doi.org/10.3114/sim.55.1.235>

Crous PW, Wingfield MJ, Roux JJ, Le R, Richardson DM, Strasberg D, Shivas RG, Alvarado P, Edwards J, Moreno G, Sharma R, Sonawane MS, Tan YP, Altés A, Barasubiye T, Barnes CW, Blanchette RA, Boertmann D, Bogo A, Carlavilla JR, Cheewangkoon R, Daniel R, de Beer ZW, de Jesús Yáñez-Morales M, Duong TA, Fernández-Vicente J, Geering ADW, Guest DI, Held BW, Heykoop M, Hubka V, Ismail AM, Kajale SC, Khemmuk W, Kolařík M, Kurli R, Lebeuf R, Lévesque CA, Lombard L, Magista D, Manjón JL, Marincowitz S, Mohedano JM, Nováková A, Oberlies NH, Otto EC, Paguigan ND, Pascoe IG, Pérez-Butrón JL, Perrone G, Rahi P, Raja HA, Rintoul T, Sanhueza RMV, Scarlett K, Shouche YS, Shuttleworth LA, Taylor PWJ, Thorn RG, Vawdrey LL, Solano-Vidal R, Voitk A, Wong PTW, Wood AR, Zamora JC, Groenewald JZ (2015) Fungal Planet description sheets: 371–399. Persoonia 35: 264–327. <https://doi.org/10.3767/003158515X690269>

Crous PW, Wingfield MJ, Richardson DM, Le Roux JJ, Strasberg D, Edwards J, Roets F, Hubka V, Taylor PW, Heykoop M, Martín MP, Moreno G, Sutton DA, Wiederhold NP, Barnes CW, Carlavilla JR, Gené J, Giraldo A, Guarnaccia V, Guarro J, Hernández-Restrepo M, Kolařík M, Manjón JL, Pascoe IG, Popov ES, Sandoval-Denis M, Woudenberg JH, Acharya K, Alexandrova AV, Alvarado P, Barbosa RN, Baseia IG, Blanchette RA, Boekhout T, Burgess TI, Cano-Lira JF, Čmoková A, Dimitrov RA, Dyakov MY, Dueñas M, Dutta AK, Esteve-Raventós F, Fedosova AG, Fournier J, Gamboa P, Gouliamova DE, Grebenc T, Groenewald M, Hanse B, Hardy GE, Held BW, Jurjević Ž, Kaewgrajang T, Latha KP, Lombard L, Luangsa-Ard JJ, Lysková P, Mallátová N, Manimohan P, Miller AN, Mirabolfathy M, Morozova OV, Obodai M, Oliveira NT, Ordóñez ME, Otto EC, Paloi S, Peterson SW, Phosri C, Roux J, Salazar WA, Sánchez A, Sarria GA, Shin HD, Silva BD, Silva GA, Smith MT, Souza-Motta CM, Stchigel AM, Stoilova-Disheva MM, Sulzbacher MA, Telleria MT, Toapanta C, Traba JM, Valenzuela-Lopez N, Watling R, Groenewald JZ (2016) Fungal Planet description sheets: 400–468. Persoonia 36: 316–458. <https://doi.org/10.3767/003158516X692185>

Crous PW, Schumacher RK, Akulov A, Thangavel R, Hernández-Restrepo M, Carnegie AJ, Cheewangkoon R, Wingfield MJ, Summerell BA, Quaedvlieg W, Coutinho TA, Roux J, Wood AR, Giraldo A, Groenewald JZ (2019) New and interesting fungi. 2. Fungal Systematics and Evolution 3: 57–134. https://doi.org/10.3114/fuse.2019.03.06

Crous PW, Cowan DA, Maggs-Kölling G, Yilmaz N, Thangavel R, Wingfield MJ, Groenewald JZ (2021) Fungal Planet description sheets: 1182–1283. Persoonia: Molecular Phylogeny and Evolution of Fungi 46: 313. http://doi.org/10.3767/persoonia.2021.46.11

Dai DQ, Wijayawardene NN, Dayarathne MC, Kumla J, Han LS, Zhang GQ, Chen HH (2022) Taxonomic and phylogenetic characterizations reveal four new species, two new asexual morph reports, and six new country records of bambusicolous *Roussoella* from China. Journal of Fungi 8(5): 532. https://doi.org/10.3390/jof8050532

Crous PW, Schumacher RK, Wingfield MJ, Akulov A, Denman S, Roux J, Braun U, Burgess TI, Carnegie AJ, Váczy KZ, Guatimosim E, Schwartsburd PB, Barreto RW, Hernández-Restrepo M, Lombard L, Groenewald JZ (2018a) New and interesting fungi. 1. Fungal Systematics and Evolution 1(1): 169–215. <https://doi.org/10.3114/fuse.2018.01.08>

Crous PW, Wingfield MJ, Burgess TI, Hardy GEStJ, Gené J, Guarro J, Baseia IG, García D, Gusmão LFP, Souza-Motta CM, Thangavel R, Adamčík S, Barili A, Barnes CW, Bezerra JDP, Bordallo JJ, Cano-Lira JF, de Oliveira RJV, Ercole E, Hubka V, Iturrieta-González I, Kubátová A, Martín MP, Moreau P-A, Morte A, Ordoñez ME, Rodríguez A, Stchigel AM, Vizzini A, Abdollahzadeh J, Abreu VP, Adamčíková K, Albuquerque GMR, Alexandrova AV, Álvarez Duarte E, Armstrong-Cho C, Banniza S, Barbosa RN, Bellanger J-M, Bezerra JL, Cabral TS, Caboň M, Caicedo E, Cantillo T, Carnegie AJ, Carmo LT, Castañeda-Ruiz RF, Clement CR, Čmoková A, Conceição LB, Cruz RHSF, Damm U, da Silva BDB, da Silva GA, da Silva RMF, de A Santiago ALCM, de Oliveira LF, de Souza CAF, Déniel F, Dima B, Dong G, Edwards J, Félix CR, Fournier J, Gibertoni TB, Hosaka K, Iturriaga T, Jadan M, Jany J-L, Jurjević Ž, Kolařík M, Kušan I, Landell MF, Leite Cordeiro TR, Lima DX, Loizides M, Luo S, Machado AR, Madrid H, Magalhães OMC, Marinho P, Matočec N, Mešić A, Miller AN, Morozova OV, Neves RP, Nonaka K, Nováková A, Oberlies NH, Oliveira-Filho JRC, Oliveira TGL, Papp V, Pereira OL, Perrone G, Peterson SW, Pham THG, Raja HA, Raudabaugh DB, Řehulka J, Rodríguez-Andrade E, Saba M, Schauflerová A, Shivas RG, Simonini G, Siqueira JPZ, Sousa JO, Stajsic V, Svetasheva T, Tan YP, Tkalčec Z, Ullah S, Valente P, Valenzuela-Lopez N, Abrinbana M, Viana Marques DA, Wong PTW, Xavier de Lima V, Groenewald JZ (2018b) Fungal Planet description sheets: 716–784. Persoonia: Molecular Phylogeny and Evolution of Fungi 40: 240. <https://doi.org/10.3767/persoonia.2018.40.10>

Crous PW, Luangsa-ard JJ, Wingfield MJ, Carnegie AJ, Hernández-Restrepo M, Lombard L, Roux J, Barreto RW, Baseia IG, Cano-Lira JF, Martín MP, Morozova OV, Stchigel AM, Summerell BA, Brandrud TE, Dima B, García D, Giraldo A, Guarro J, Gusmão LFP, Khamsuntorn P, Noordeloos ME, Nuankaew S, Pinruan U, Rodríguez-Andrade E, Souza-Motta CM, Thangavel R, van Iperen AL, Abreu VP, Accioly T, Alves JL, Andrade JP, Bahram M, Baral HO, Barbier E, Barnes CW, Bendiksen E, Bernard E, Bezerra JDP, Bezerra JL, Bizio E, Blair JE, Bulyonkova TM, Cabral TS, Caiafa MV, Cantillo T, Colmán AA, Conceição LB, Cruz S, Cunha AOB, Darveaux BA, da Silva AL, da Silva GA, da Silva GM, da Silva RMF, de Oliveira RJV, Oliveira RL, De Souza JT, Dueñas M, Evans HC, Epifani F, Felipe MTC, Fernández-López J, Ferreira BW, Figueiredo CN, Filippova NV, Flores JA, Gené J, Ghorbani G, Gibertoni TB, Glushakova AM, Healy R, Huhndorf SM, Iturrieta-González I, Javan-Nikkhah M, Juciano RF, Jurjević Ž, Kachalkin AV, Keochanpheng K, Krisai-Greilhuber I, Li YC, Lima AA, Machado AR, Madrid H, Magalhães OMC, Marbach PAS, Melanda GCS, Miller AN, Mongkolsamrit S, Nascimento RP, Oliveira TGL, Ordoñez ME, Orzes R, Palma MA, Pearce CJ, Pereira OL, Perrone G, Peterson SW, Pham THG, Piontelli E, Pordel A, Quijada L, Raja HA, Rosas de Paz E, Ryvarden L, Saitta A, Salcedo SS, Sandoval-Denis M, Santos TAB, Seifert KA, Silva BDB, Smith ME, Soares AM, Sommai S, Sousa JO, Suetrong S, Susca A, Tedersoo L, Telleria MT, Thanakitpipattana D, Valenzuela-Lopez N, Visagie CM, Zapata M, Groenewald JZ (2018c) Fungal Planet description sheets: 785–867. Persoonia-Molecular Phylogeny and Evolution of Fungi 41(1): 238–417. https://doi.org/10.3767/persoonia.2018.41.12

Crous PW, Wingfield MJ, Schumacher RK, Summerell BA, Giraldo A, Gené J, Guarro J, Wanasinghe DN, Hyde KD, Camporesi E, Garethjones EB, Thambugala KM, Malysheva EF, Malysheva VF, Acharya K, Álvarez J, Alvarado P, Assefa A, Barnes CW, Bartlett JS, Blanchette RA, Burgess TI, Carlavilla JR, Coetzee MPA, Damm U, Decock CA, Denbreeÿen A, Devries B, Dutta AK, Holdom DG, Rooney-Latham S, Manjón JL, Marincowitz S, Mirabolfathy M, Moreno G, Nakashima C, Papizadeh M, Shahzadehfazeli SA, Amoozegar MA, Romberg MK, Shivas RG, Stalpers JA, Stielow B, Stukely MJC, Swart WJ, Tan YP, Vanderbank M, Wood AR, Zhang Y, Groenewald JZ (2014) Fungal Planet description sheets: 281–319. Persoonia-Molecular Phylogeny and Evolution of Fungi 33(1): 212–289. https://doi.org/10.3767/003158514X685680

Crous PW, Carnegie AJ, Wingfield MJ, Sharma R, Mughini G, Noordeloos ME, Santini A, Shouche YS, Bezerra JDP, Dima B, Guarnaccia V, Imrefi I, Jurjević Ž, Knapp DG, Kovács GM, Magistà D, Perrone G, Rämä T, Rebriev YA, Shivas RG, Singh SM, Souza-Motta CM, Thangavel R, Adhapure NN, Alexandrova AV, Alfenas AC, Alfenas RF, Alvarado P, Alves AL, Andrade DA, Andrade JP, Barbosa RN, Barili A, Barnes CW, Baseia IG, Bellanger J-M, Berlanas C, Bessette AE, Bessette AR, Biketova AYu, Bomfim FS, Brandrud TE, Bransgrove K, Brito ACQ, Cano-Lira JF, Cantillo T, Cavalcanti AD, Cheewangkoon R, Chikowski RS, Conforto C, Cordeiro TRL, Craine JD, Cruz R, Damm U, de Oliveira RJV, de Souza JT, de Souza HG, Dearnaley JDW, Dimitrov RA, Dovana F, Erhard A, Esteve-Raventós F, Félix CR, Ferisin G, Fernandes RA, Ferreira RJ, Ferro LO, Figueiredo CN, Frank JL, Freire KTLS, García D, Gené J, Gêsiorska A, Gibertoni TB, Gondra RAG, Gouliamova DE, Gramaje D, Guard F, Gusmão LFP, Haitook S, Hirooka Y, Houbraken J, Hubka V, Inamdar A, Iturriaga T, Iturrieta-González I, Jadan M, Jiang N, Justo A, Kachalkin AV, Kapitonov VI, Karadelev M, Karakehian J, Kasuya T, Kautmanová I, Kruse J, Kušan I, Kuznetsova TA, Landell MF, Larsson K-H, Lee HB, Lima DX, Lira CRS, Machado AR, Madrid H, Magalhães OMC, Majerova H, Malysheva EF, Mapperson RR, Marbach PAS, Martín MP, Martín-Sanz A, Matočec N, McTaggart AR, Mello JF, Melo RFR, Mešić A, Michereff SJ, Miller AN, Minoshima A, Molinero-Ruiz L, Morozova OV, Mosoh D, Nabe M, Naik R, Nara K, Nascimento SS, Neves RP, Olariaga I, Oliveira RL, Oliveira TGL, Ono T, Ordoñez ME, Ottoni A de M, Paiva LM, Pancorbo F, Pant B, Pawłowska J, Peterson SW, Raudabaugh DB, Rodríguez-Andrade E, Rubio E, Rusevska K, Santiago ALCMA, Santos ACS, Santos C, Sazanova NA, Shah S, Sharma J, Silva BDB, Siquier JL, Sonawane MS, Stchigel AM, Svetasheva T, Tamakeaw N, Telleria MT, Tiago PV, Tian CM, Tkalčec Z, Tomashevskaya MA, Truong HH, Vecherskii MV, Visagie CM, Vizzini A, Yilmaz N, Zmitrovich IV, Zvyagina EA, Boekhout T, Kehlet T, Læssøe T, Groenewald JZ (2019) Fungal Planet description sheets: 868–950. Persoonia: Molecular Phylogeny and Evolution of Fungi 42: 291. https://doi.org/10.3767/persoonia.2019.42.11

Crous PW, Summerell BA, Swart L, Denman S, Taylor JE, Bezuidenhout CM, Palm ME, Marincowitz S, Groenewald JZ (2011) Fungal pathogens of *Proteaceae*. Persoonia-Molecular Phylogeny and Evolution of Fungi 27(1): 20–45. <https://doi.org/10.3767/003158511X606239>

Crous PW, Carris LM, Giraldo A, Groenewald JZ, Hawksworth DL, Hernández-Restrepo M, Jaklitsch WM, Lebrun MH, Schumacher RK, Stielow JB, van der Linde EJ, Vilcāne J, Voglmayr H, Wood AR (2015) The genera of fungi-fixing the application of the type species of generic names-G 2: *Allantophomopsis, Latorua, Macrodiplodiopsis, Macrohilum, Milospium, Protostegia, Pyricularia, Robillarda, Rotula, Septoriella, Torula*, and *Wojnowicia*. IMA Fungus 6: 163–198. <https://doi.org/10.5598/imafungus.2015.06.01.11>

Crous PW Summerell BA Shivas RG Burgess TI Decock CA Dreyer LL Granke LL Guest DI Hardy GE STJ Hausbeck MK Hüberli D Jung T Koukol O Lennox CL Liew ECY Lombard L McTaggart AR Pryke JS Roets F Saude C Shuttleworth LA Stukely MJC Vánky K Webster BJ Windstam ST Groenewald JZ (2012) Fungal Planet description sheets: 107–127. Persoonia 28: 138–182. https://doi.org/10.3767/003158512X652633

Crous PW Wingfield MJ Chooi YH Gilchrist CLM Lacey E Pitt JI Roets F Swart WJ Cano-Lira JF Valenzuela-Lopez N Hubka V Shivas RG Stchigel AM Holdom DG Jurjević Ž Kachalkin AV Lebel T Lock C Martín MP Tan YP Tomashevskaya MA Vitelli JS Baseia IG Bhatt VK Brandrud TE De Souza JT Dima B Lacey HJ Lombard L Johnston PR Morte A Papp V Rodríguez A Rodríguez-Andrade E Semwal KC Tegart L Abad ZG Akulov A Alvarado P Alves A Andrade JP Arenas F Asenjo C Ballarà J Barrett MD Berná LM Berraf-Tebbal A Bianchinotti MV Bransgrove K Burgess TI Carmo FS Chávez R Čmoková A Dearnaley JDW de A Santiago ALCM Freitas-Neto JF Denman S Douglas B Dovana F Eichmeier A Esteve-Raventós F Farid A Fedosova AG Ferisin G Ferreira RJ Ferrer A Figueiredo CN Figueiredo YF Reinoso-Fuentealba CG Garrido-Benavent I Cañete-Gibas CF Gil-Durán C Glushakova AM Gonçalves MFM González M Gorczak M Gorton C Guard FE Guarnizo AL Guarro J Gutiérrez M Hamal P Hien LT Hocking AD Houbraken J Hunter GC Inácio CA Jourdan M Kapitonov VI Kelly L Khanh TN Kisło K Kiss L Kiyashko A Kolařík M Kruse J Kubátová A Kučera V Kučerová I Kušan I Lee HB Levicán G Lewis A Liem NV Liimatainen K Lim HJ Lyons MN Maciá-Vicente JG Magaña-Dueñas V Mahiques R Malysheva EF Marbach PAS Marinho P Matočec N McTaggart AR Mešić A Morin L Muñoz-Mohedano JM Navarro-Ródenas A Nicolli CP Oliveira RL Otsing E Ovrebo CL Pankratov TA Paños A Paz-Conde A Pérez-Sierra A Phosri C Pintos Á Pošta A Prencipe S Rubio E Saitta A Sales LS Sanhueza L Shuttleworth LA Smith J Smith ME Spadaro D Spetik M Sochor Z Sochorová Z Sousa JO Suwannasai N Tedersoo L Thanh HM Thao LD Tkalčec Z Vaghefi N Venzhik AS Verbeken A Vizzini A Voyron S Wainhouse M Whalley AJS Wrzosek M Zapata M Zeil-Rolfe I Groenewald JZ (2020) Fungal Planet description sheets: 1042–1111. Persoonia 44: 301–459. <https://doi.org/10.3767/persoonia.2020.44.11>

Crous PW, Wingfield MJ, Schumacher RK, Summerell BA, Giraldo A, Gené J, Guarro J, Wanasinghe DN, Hyde KD, Camporesi E, Garethjones EB, Thambugala KM, Malysheva EF, Malysheva VF, Acharya K, Álvarez J, Alvarado P, Assefa A, Barnes CW, Bartlett JS, Blanchette RA, Burgess TI, Carlavilla JR, Coetzee MPA, Damm U, Decock CA, Denbreeÿen A, Devries B, Dutta AK, Holdom DG, Rooney-Latham S, Manjón JL, Marincowitz S, Mirabolfathy M, Moreno G, Nakashima C, Papizadeh M, Shahzadehfazeli SA, Amoozegar MA, Romberg MK, Shivas RG, Stalpers JA, Stielow B, Stukely MJ, Swart WJ, Tan YP, Vanderbank M, Wood AR, Zhang Y, Groenewald JZ (2014) Fungal Planet description sheets: 281–319. Persoonia-Molecular Phylogeny and Evolution of Fungi 33(1): 212–289. <https://doi.org/10.3767/003158514X685680>

Chomnunti P, Schoch CL, Aguirre-Hudson B, Ko-Ko TW, Hongsanan S, Jones EG, Kodsueb R, Phookamsak R, Chukeatirote E, Bahkali AH, Hyde KD (2011) *Capnodiaceae*. Fungal Diversity 51: 103–34. https://doi.org/10.1007/s13225-011-0145-6

de Silva NI, Hyde KD, Lumyong S, Phillips AJL, Bhat DJ, Maharachchikumbura SSN, Thambugala KM, Tennakoon DS, Suwannarach N, Karunarathna SC (2022) Morphology, phylogeny, host association, and geography of fungi associated with plants of *Annonaceae*, *Apocynaceae*, and *Magnoliaceae*. Mycosphere 13(1): 955–1076. https://doi.org/10.5943/mycosphere/13/1/12

Du TY, Hyde KD, Mapook A, Mortimer PE, Xu J, Karunarathna SC, Tibpromma S (2021) Morphology and phylogenetic analyses reveal *Montagnula puerensis* sp. nov. (*Didymosphaeriaceae, Pleosporales*) from southwest China. Phytotaxa 514(1): 1–25. <https://doi.org/10.11646/phytotaxa.514.1.1>

Du TY, Dai DQ, Mapook A, Lu L, Stephenson SL, Suwannarach N, Elgorban AM, Al-Rejaie S, Karunarathna SC, Tibpromma S (2023) Additions to *Rhytidhysteron* (*Hysteriales, Dothideomycetes*) in China. Journal of Fungi 9(2): 148. https://doi.org/10.3390/jof9020148

Dai DQ, Bhat DJ, Liu JK, Chukeatirote E, Zhao RL, Hyde KD (2012) *Bambusicola*, a new genus from bamboo with asexual and sexual morphs. Cryptogamie Mycologie 33: 363–379. <https://doi.org/10.7872/crym.v33.iss3.2012.363>

Dai DQ, Bahkali AH, Li WJ, Bhat DJ, Zhao RL, Hyde KD (2015) *Bambusicola loculata* sp. nov. (*Bambusicolaceae*) from bamboo. Phytotaxa 213: 122–130. <https://doi.org/10.11646/phytotaxa.213.2.5>

Dai DQ, Phookamsak R, Wijayawardene NN, Li WJ, Bhat DJ, Xu JC, Taylor JE, Hyde KD, Chukeatirote E (2016) Bambusicolous fungi. Fungal Diversity 82: 1–105. <https://doi.org/10.1007/s13225-016-0367-8>

Das K, Lee SY, Jung HY (2021) Morphology and phylogeny of two novel species within the class *Dothideomycetes* collected from soil in Korea. Mycobiology 49(1): 15–23. <https://doi.org/10.1080/12298093.2020.1838114>

Dubey R (2021) *Didymocrea leucaenae:* A new record to Indian mycoflora. MycoAsia – Journal of Modern Mycology. <https://doi.org/10.59265/mycoasia.2021-02>

Devadatha B, Sarma VV, Jeewon R, Wanasinghe DN, Hyde KD, Gareth Jones EB (2018) *Thyridariella*, a novel marine fungal genus from India: morphological characterization and phylogeny inferred from multigene DNA sequence analyses. Mycological Progress 17: 791–804. <https://doi.org/10.1007/s11557-018-1387-4>

Doilom M, Dissanayake AJ, Wanasinghe DN, Boonmee S, Liu JK, Bhat DJ, Hyde KD (2017) Microfungi on *Tectona grandis* (teak) in northern Thailand. Fungal Diversity 82: 107–182. https://doi.org/10.1007/s13225-016-0368-7

Dong W, Wang B, Hyde KD, McKenzie EH, Raja HA, Tanaka K, Abdel-Wahab MA, Abdel-Aziz FA, Doilom M, Phookamsak R, Hongsanan S, Wanasinghe DN, Yu XD, Wang GN, Yang H, Yang J, Thambugala KM, Tian Q, Luo ZL, Yang JB, Miller AN, Fournier J, Boonmee S, Hu DM, Nalumpang S, Zhang H (2020) Freshwater *Dothideomycetes*. Fungal Diversity 105: 319–575. https://doi.org/10.1007/s13225-020-00463-5

Feng Y, Zhang SN, Liu ZY (2019) *Tremateia murispora* sp. nov. (*Didymosphaeriaceae*, *Pleosporales*) from Guizhou, China. Phytotaxa 416(1): 79–87. <https://doi.org/10.11646/phytotaxa.416.1.10>

Gizaw B, Tsegaye Z, Tefera G (2016) Isolation, identification and characterization of yeast species from coffee waste collected from Sidama and Gedio zone. Journal of Yeast and Fungal Research 7(6): 47–53. <https://doi.org/10.5897/JYFR2016.0174>

Guo YL (1999) Imperfect fungi in the tropical areas of China II. Some species of *Pseudocercospora* from Guangdong Province. Mycosystema 18: 130–134.

Gonçalves MF, Vicente TFL, Esteves AC, Alves A (2019) *Neptunomyces aureus* gen. et sp. nov. (*Didymosphaeriaceae*, *Pleosporales*) isolated from algae in Ria de Aveiro, Portugal. MycoKeys 60: 31–44. https://doi.org/10.3897/mycokeys.60.37931

Hu YF, Liu JW, Xu ZH, Castañeda-Ruíz RF, Zhang K, Ma J (2023) Morphology and multigene phylogeny revealed three new species of *Helminthosporium* (*Massarinaceae*, *Pleosporales*) from China. Journal of Fungi 9(2): 280. https://doi.org/10.3390/jof9020280

Hu FJ, Jeewon R, Hyde KD (2009) Relationships among *Astrosphaeriella, Caryospora* and *Trematosphaeria* (Doctoral dissertation, The University of Hong Kong).

Hirayama K, Tanaka K, Raja HA, Miller AN, Shearer CA (2010) A molecular phylogenetic assessment of *Massarina ingoldiana* sensu lato. Mycologia 102(3): 729-746. https://doi.org/10.3852/09-230

Hashimoto A, Hirayama K, Takahashi H, Matsumura M, Okada G, Chen CY, Tanaka K (2018) Resolving the *Lophiostoma bipolare* complex: Generic delimitations within *Lophiostomataceae*. Studies in Mycology 90(1): 161–189. https://doi.org/10.1016/j.simyco.2018.03.001

Hatakeyama S, Tanaka K, Harada Y (2008) Bambusicolous fungi in Japan (7): A new coelomycetous genus, *Versicolorisporium*. Mycoscience 49(3): 211–214. https://doi.org/10.1007/s10267-008-0409-5

Hyde KD, Chaiwan N, Norphanphoun C, Boonmee S, Camporesi E, Chethana KWT, Dayarathne MC, de Silva NI, Dissanayake AJ, Ekanayaka AH, Hongsanan S, Huang SK, Jayasiri SC, Jayawardena RS, Jiang HB, Karunarathna A, Lin CG, Liu JK, Liu NG, Lu YZ, Luo ZL, Maharachchimbura SSN, Manawasinghe IS, Pem D, Perera RH, Phukhamsakda C, Samarakoon MC, Senwanna C, Shang QJ, Tennakoon DS, Thambugala KM, Tibpromma S, Wanasinghe DN, Xiao YP, Yang J, Zeng XY, Zhang JF, Zhang SN, Bulgakov TS, Bhat DJ, Cheewangkoon R, Goh TK, Jones EBG, Kang JC, Jeewon R, Liu ZY, Lumyong S, Kuo CH, McKenzie EHC, Wen TC, Yan JY, Zhao Q (2018) Mycosphere notes 169–224. Mycosphere 9(2): 271–430. https://doi.org/10.5943/mycosphere/9/2/8

Hyde KD, Wijesinghe SN, Afshari N, Aumentado HD, Bhunjun CS, Boonmee S, Camporesi E, Chethana KWT, Doilom M, Dong W, Du TY, Farias ARG, Gao Y, Jayawardena RS, Karimi O, Karunarathna SC, Kularathnage ND, Lestari AS, Li CJY, Li YX, Liao CF, Liu XF, Lu L, Lu YZ, Luo ZL, Ma J, Mamarabadi M, Manawasinghe IS, Mapook A, Mi LX, Niranjan M, Senanayake IC, Shen HW, Su HL, Tibpromma S, Xu RJ, Yan JY, Yang YH, Yang YY, Yu FQ, Kang JC, Zhang JY (2024) Mycosphere Notes 469–520. Mycosphere 15(1): 1294–454. https://doi.org/10.5943/mycosphere/15/1/11

Hyde KD, Norphanphoun C, Ma J, Yang HD, Zhang JY, Du TY, Gao Y, Gomes de Farias AR, Gui H, He SC, He YK, Li CJY, Liu XF, Lu L, Su HL, Tang X, Tian XG, Wang SY, Wei DP, Xu RF, Xu RJ, Yang Q, Yang YY, Zhang F, Zhang Q, Bahkali AH, Boonmee S, Chethana KWT, Jayawardena RS, Lu YZ, Karunarathna SC, Tibpromma S, Wang Y, Zhao Q (2023) Mycosphere notes 387–412: novel species of fungal taxa from around the world. Mycosphere 14(1): 663–744. https://doi.org/10.5943/mycosphere/14/1/8

Hyde KD, Dong Y, Phookamsak R, Jeewon R, Bhat DJ, Jones EBG, Liu NG, Abeywickrama PD, Mapook A, Wei DP, Perera RH, Manawasinghe IS, Pem D, Bundhun D, Karunarathna A, Ekanayaka AH, Bao DF, Li JF, Samarakoon MC, Chaiwan N, Lin CG, Phutthacharoen K, Zhang SN, Senanayake IC, Goonasekara ID, Thambugala KM, Phukhamsakda C, Tennakoon DS, Jiang HB, Yang J, Zeng M, Huanraluek N, Liu JK, Wijesinghe SN, Tian Q, Tibpromma S, Brahmanage RS, Boonmee S, Huang SK, Thiyagaraja V, Lu YZ, Jayawardena RS, Dong W, Yang EF, Singh SK, Singh SM, Rana S, Lad SS, Anand G, Devadatha B, Niranjan M, Sarma VV, Liimatainen K, Aguirre-Hudson B, Niskanen T, Overall A, Alvarenga RLM, Gibertoni TB, Pfliegler WP, Horváth E, Imre A, Alves AL, Santos ACS, Tiago PV, Bulgakov TS, Wanasinghe DN, Bahkali AH, Doilom M, Elgorban AM, Maharachchikumbura SSN, Rajeshkumar KC, Haelewaters D, Mortimer PE, Zhao Q, Lumyong S, Xu JC (2020) Fungal diversity notes 1151–1276: taxonomic and phylogenetic contributions on genera and species of fungal taxa. Fungal Diversity 100: 5–277. https://doi.org/10.1007/s13225-020-00439-5

Hyde KD, Hongsanan S, Jeewon R, Bhat DJ, McKenzie EHC, Jones EBG, Phookamsak R, Ariyawansa HA, Boonmee S, Zhao Q, Abdel-Aziz FA, Abdel-Wahab MA, Banmai S, Chomnunti P, Cui BK, Daranagama DA, Das K, Dayarathne MC, de Silva NI, Dissanayake AJ, Doilom M, Ekanayaka AH, Gibertoni TB, Góes-Neto A, Huang SK, Jayasiri SC, Jayawardena RS, Konta S, Lee HB, Li WJ, Lin CG, Liu JK, Lu YZ, Luo ZL, Manawasinghe IS, Manimohan P, Mapook A, Niskanen T, Norphanphoun C, Papizadeh M, Perera RH, Phukhamsakda C, Richter C, de Santiago ALCMA, Drechsler-Santos ER, Senanayake IC, Tanaka K, Tennakoon TMDS, Thambugala KM, Tian Q, Tibpromma S, Thongbai B, Vizzini A, Wanasinghe DN, Wijayawardene NN, Wu HX, Yang J, Zeng XY, Zhang H, Zhang JF, Bulgakov TS, Camporesi E, Bahkali AH, Amoozegar AM, Araujo-Neta LS, Ammirati JF, Baghela A, Bhatt RP, Bojantchev S, Buyck B, da Silva GA, de Lima CLF, de Oliveira RJV, de Souza CAF, Dai YC, Dima B, Duong TT, Ercole E, Mafalda-Freire F, Ghosh A, Hashimoto A, Kamolhan S, Kang JC, Karunarathna SC, Kirk PM, Kyto¨vuori I, Lantieri A, Liimatainen K, Liu ZY, Liu XZ, Lücking R, Medardi G, Mortimer PE, Nguyen TT, Promputtha I, Raj KNA, Reck MA, Lumyong S, Shahzadeh-Fazeli SA, Stadler M, Soudi MR, Su HY, Takahashi T, Tangthirasunun N, Uniyal P, Wang Y, Wen TC, Xu JC, Zhang ZK, Zhao YC, Zhou JZ, Zhu L (2016) Fungal diversity notes 367–490: taxonomic and phylogenetic contributions to fungal taxa. Fungal Diversity 80: 1–270. <https://doi.org/10.1007/s13225-016-0373-x>

Hyde KD, Norphanphoun C, Abreu VP, Bazzicalupo A, Chethana KWT, Clericuzio M, Dayarathne MC, Dissanayake AJ, Ekanayaka AH, He MQ, Hongsanan S, Huang SK, Jayasiri SC, Jayawardena RS, Karunarathna A, Konta S, Kušan I, Lee H, Li JF, Lin CG, Liu NG, Lu YZ, Luo ZL, Manawasinghe IS, Mapook A, Perera RH, Phookamsak R, Phukhamsakda C, Siedlecki I, Soares AM, Tennakoon DS, Tian Q, Tibpromma S, Wanasinghe DN, Xiao YP, Yang J, Zeng XY, Abdel-Aziz FA, Li WJ, Senanayake IC, Shang QJ, Daranagama DA, de Silva NI, Thambugala KM, Abdel-Wahab MA, Bahkali AH, Berbee ML, Boonmee S, Bhat DJ, Bulgakov TS, Buyck B, Camporesi E, Castañeda-Ruiz RF, Chomnunti P, Doilom M, Dovana F, Gibertoni TB, Jadan M, Jeewon R, Jones EBG, Kang JC, Karunarathna SC, Lim YW, Liu JK, Liu ZY, Plautz Jr HL, Lumyong S, Maharachchikumbura SSN, Matočec N, McKenzie EHC, Mešić A, Miller D, Pawłowska J, Pereira OL, Promputtha I, Romero AI, Ryvarden L, Su HY, Suetrong S, Tkalčec Z, Vizzini A, Wen TC, Wisitrassameewong K, Wrzosek M, Xu JC, Zhao Q, Zhao RL, Mortimer PE (2017) Fungal diversity notes 603–708: taxonomic and phylogenetic notes on genera and species. Fungal Diversity 87: 1–235. <https://doi.org/10.1007/s13225-017-0391-3>

Hou LW, Groenewald JZ, Pfenning LH, Yarden O, Crous PW, Cai L (2020) The phoma-like dilemma. Studies in Mycology 96: 309–396. <https://doi.org/10.1016/j.simyco.2020.05.001>

Hongsanan S, Hyde KD, Bahkall AH, Camporesi BE, Chomnunti P, Ekanayaka H, Gomes AAM, Hofstetter V, Jones EBG, Pinho DB, Pereira OL, Tian Q, Wansinghe DN, Xu JC, Buyck B (2015) Fungal biodiversity profiles 11–20. Cryptogamie Mycologie 36: 355–380. https://doi.org/10.7872/crym/v36.iss3.2015.355.

James TY, Kauff F, Schoch CL, Matheny PB, Hofstetter V, Cox CJ, Vilgalys R (2006) Reconstructing the early evolution of fungi using a six-gene phylogeny. Nature 443(7113): 818–822.

Jayasiri SC, Hyde KD, Jones EBG, McKenzie EH, Jeewon R, Phillips AJL, Karunarathna SC (2019) Diversity, morphology and molecular phylogeny of *Dothideomycetes* on decaying wild seed pods and fruits. MycoKeys 56: 1–25. <https://doi.org/10.5943/mycosphere/10/1/1>

Jaklitsch WM, Voglmayr H (2016) Hidden diversity in *Thyridaria* and a new circumscription of the *Thyridariaceae*. Studies in Mycology 85: 35–64. <https://doi.org/10.1016/j.simyco.2016.09.002>

Jayasiri SC (2016) *Neooccultibambusa jonesii*, a novel taxon within *Occultibambusaceae*. Mycosphere 7(9): 1458–1472. https://doi.org/10.5943/mycosphere/7/9/17

Jaklitsch WM, Checa J, Blanco MN, Olariaga I, Tello S, Voglmayr H (2018) A preliminary account of the *Cucurbitariaceae*. Studies in Mycology 90: 71–118. https://doi.org/10.1016/j.simyco.2017.11.002

Jaklitsch WM, Voglmayr H (2020) Fenestelloid clades of the *Cucurbitariaceae*. Persoonia-Molecular Phylogeny and Evolution of Fungi 44(1): 1–40. https://doi.org/10.3767/persoonia.2020.44.01

Jiang HB, Hyde KD, Jayawardena RS, Doilom M, Xu JC, Phookamsak R (2019) Taxonomic and phylogenetic characterizations reveal two new species and two new records of *Roussoella* (*Roussoellaceae*, *Pleosporales*) from Yunnan, China. Mycological Progress 18: 577–591. https://doi.org/10.1007/s11557-019-01471-9

Kruys Å, Eriksson OE, Wedin M (2006) Phylogenetic relationships of coprophilous *Pleosporales* (*Dothideomycetes*, *Ascomycota*), and the classification of some bitunicate taxa of unknown position. Mycological Research 110: 527–536. <https://doi.org/10.1016/j.mycres.2006.03.002>

Karunarathna A, Phookamsak R, Jayawardena RS, Cheewangkoon R, Hyde KD, Kuo CH (2019) The holomorph of *Neoroussoella alishanense* sp. nov. (*Roussoellaceae, Pleosporales*) on *Pennisetum purpureum* (*Poaceae*). Phytotaxa 406(4): 218–236. <https://doi.org/10.11646/phytotaxa.406.4.1>

Kohlmeyer B, Sakayaroj J, Phongpaichit S, Tanaka K, Hirayama K, Jones EB (2009) Molecular systematics of the marine *Dothideomycetes*. Studies in Mycology 64(1): 155–173. https://doi.org/10.3114/sim.2009.64.09

Liew EC, Aptroot A, Hyde KD (2002) An evaluation of the monophyly of *Massarina* based on ribosomal DNA sequences. Mycologia 94(5): 803–813. <https://doi.org/10.1080/15572536.2003.11833174>

Liu YJ, Whelen S, Hall BD (1999) Phylogenetic relationships among ascomycetes: evidence from an RNA polymerse II subunit. Molecular Biology and Evolution 16: 1799–1808. <https://doi.org/10.1093/oxfordjournals.molbev.a026092>.

Liu SL, Zhao P, Cai L, Shen S, Wei HW, Na Q, Han M, Wei RX, Ge YP, Ma HX, Karunarathna SC, Tibpromma S, Zhang B, Dai D, Lin L, Fan XL, Luo ZL, Shen HW, Lu L, Lu WH, Xu RF, Tohtirjap A, Wu F, Zhou LW (2024a) Catalogue of fungi in China 1. New taxa of plant-inhabiting fungi. Mycology 1–58. https://doi.org/10.1080/21501203.2024.2316066.

Liu XF, Tibpromma S, Karunarathna SC, Chethana KW, Lu L, Dai D, Elgorban AM, Hyde KD (2024b) Morphology and multi-gene phylogeny reveal a new *Brunneofusispora* species from coffee in Yunnan Province, China. Phytotaxa 634(1): 16–30. https://doi.org/10.11646/phytotaxa.634.1.2.

Liu JK, Hyde KD, Jones EBG, Ariyawansa HA, Bhat DJ, Boonmee S, Maharachchikumbura S, McKenzie EHC, Phookamsak R, Phukhamsakda C, Shenoy BD, Abdel-Wahab MA, Buyck B, Chen J, Chethana KW, Singtripop C, Dai DQ, Dai YC, Daranagama DA, Dissanayake AJ, Doliom M, D’souza MJ, Fan XL, Goonasekara ID, Hirayama K, Hongsanan S, Jayasiri SC, Jayawardena RS, Karunarathna SC, Li WJ, Mapook A, Norphanphoun C, Pang KL, Perera RH, Persöh D, Pinruan U, Senanayake IC, Somrithipol S, Suetrong S, Tanaka K, Thambugala KM, Tian Q, Tibpromma S, Udayanga D, Wijayawardena NN, Wanasinghe D, Wisitrassameewong K, Abdel-Aziz FA, Adamcýk S, Bahkali AH, Boonyuen N, Bulgakov T, Callac P, Chomnunti P, Greiner K, Hashimoto A, Hofstetter V, Kang JC, Lewis D, Li XH, Liu XX, Liu ZY, Matumura M, Mortimer PE, Rambold G, Randrianjohany E, Sato G, Sri-Indrasutdhi V, Tian CM, Verbeken A, von Brackel W, Wang Y, Wen TC, Xu JC, Yan JY, Zhao RL, Camporesi E (2015) Fungal Diversity notes 1–110: taxonomic and phylogenetic contributions to fungal species. Fungal Diversity 72: 1–197. <https://doi.org/10.1007/s13225-015-0324-y>.

Liu JK, Phookamsak R, Dai DQ, Tanaka K, Jones EG, Xu JC, Hyde KD (2014) *Roussoellaceae*, a new *Pleosporalean* family to accommodate the genera *Neoroussoella* gen. nov., *Roussoella* and *Roussoellopsis*. Phytotaxa 181(1): 1–33. <https://doi.org/10.11646/phytotaxa.181.1.1>

Liu J, Hu Y, Luo X, Castañeda-Ruíz RF, Ma J (2022) Three novel species of *Helminthosporium* (*Massarinaceae, Pleosporales*) from China. MycoKeys 94: 73. https://doi.org/10.3897/mycokeys.94.95888

Lu L, Karunarathna SC, Dai DQ, Xiong YR, Suwannarach N, Stephenson SL, Elgorban AM, Al-Rejaie S, Jayawardena RS, Tibpromma S (2022a) Description of four novel species in *Pleosporales* associated with coffee in Yunnan, China. Journal of Fungi 8: 1113. <https://doi.org/10.3390/jof8101113>.

Lu L, Karunarathna SC, Hyde KD, Bhat DJ, Dai DQ, Jayawardena RS, Tibpromma S (2022b) *Crassiparies yunnanensis* sp. nov. (*Neohendersoniaceae*, *Pleosporales*) from dead twigs of *Coffea arabica* in China. Phytotaxa 543(4): 244–254. <https://doi.org/10.11646/phytotaxa.543.4.4>.

Lu L, Tibpromma S, Karunarathna SC, Thiyagaraja V, Xu JC, Jayawardena RS, Lumyong S, Hyde KD (2021) Taxonomic and phylogenetic appraisal of a novel species and a new record of *Stictidaceae* from coffee in Yunnan Province, China. Phytotaxa 528(2): 111–124. <https://doi.org/10.11646/phytotaxa.528.2.4>.

Lu L, Karunarathna SC, Dai DQ, Jayawardena RS, Suwannarach N, Tibpromma S (2022c) Three new species of *Nigrograna* (*Dothideomycetes*, *Pleosporales*) associated with Arabica coffee from Yunnan Province, China. MycoKeys 94: 51. <https://doi.org/10.3897/mycokeys.94.95751>.

Lu L, Karunarathna SC, Liu YN, Elgorban AM, Tibpromma S, Jayawardena RS (2024) *Pseudoconiocessia xishuangbannaensis* gen. et sp. nov. in *Coniocessiaceae*, *Xylariales* from *Coffea liberica* in China. Phytotaxa 641(2): 125–137. <https://doi.org/10.11646/phytotaxa.641.2.4>.

Li GJ, Hyde KD, Zhao RL, Hongsanan S, Abdel-Aziz FA, Abdel-Wahab MA, Alvarado P, Alves-Silva GA, Ammirati JF, Ariyawansa HA, Baghela A, Bahkali AH, Beug M, Bhat DJ, Bojantchev D, Boonpratuang T, Bulgakov TS, Camporesi E, Boro MC, Ceska O, Chakraborty D, Chen JJ, Chethana KWT, Chomnunti P, Consiglio G, Cui BK, Dai DQ, Dai YC, Daranagama DA, Das K, Dayarathne MC, De Crop E, De Oliveira RJV, de Souza CAF, de Souza JI, Dentinger BTM, Dissanayake AJ, Doilom M, Drechsler-Santos ER, Ghobad-Nejhad M, Gilmore SP, Góes-Neto A, Gorczak M, Haitjema CH, Hapuarachchi KK, Hashimoto A, He MQ, Henske JK, Hirayama K, Iribarren MJ, Jayasiri SC, Jayawardena RS, Jeon SJ, Jerônimo GH, Jesus AL, Jones EBG, Kang JC, Karunarathna SC, Kirk PM, Konta S, Kuhnert E, Langer E, Lee HS, Lee HB, Li WJ, Li XH, Liimatainen K, Lima DX, Lin CG, Liu JK, Liu XZ, Liu ZY, Lu J, Lücking R, Lumbsch HT, Lumyong S, Leaño EM, Marano AV, Matsumura M, McKenzie EHC, Mongkolsamrit S, Mortimer PE, Nguyen TTT, Niskanen T, Norphanphoun C, O’Malley MA, Parnmen S, Pawłowska J, Perera RH, Phookamsak R, Phukhamsakda C, Pires-Zottarelli CLA, Raspé O, Reck MA, Rocha SCO, de Santiago ALCM, Senanayake IC, Setti L, Shang QJ, Singh SK, Sir EB, Solomon KV, Song J, Srikitikulchai PS, Stadler M, Suetrong S, Takahashi H, Takahashi T, Tanaka K, Tang LP, Thambugala KM, Thanakitpipattana D, Theodorou MK, Thongbai BJ, Thummarukcharoen T, Tian Q, Tibpromma S, Verbeken A, Vizzini A, Vlasák J, Voigt K, Wanasinghe DN, Wang Y, Weerakoon G, Wen HA, Wen TC, Wijayawardene NN, Wongkanoun S, Wrzosek M, Xiao YP, Xu JC, Yan JY, Yang JD, Yang YH, Hu Y, Zhang JF, Zhao J, Zhou LW, Peršoh D, Phillips AJL, Maharachchikumbura SSN (2016) Fungal diversity notes 253–366: Taxonomic and phylogenetic contributions to fungal taxa. Fungal Diversity 78: 1–237. https://doi.org/10.1007/s13225-016-0366-9

Li WJ, McKenzie EHC, Liu JK, Bhat DJ, Dai DQ, Camporesi E, Tian Q, Maharachchikumbura SSN, Luo ZL, Shang QJ, Zhang JF, Tangthirasunun N, Karunarathna SC, Xu JC, Hyde KD (2020) Taxonomy and phylogeny of hyaline-spored coelomycetes. Fungal Diversity 100: 279–801. https://doi.org/10.1007/s13225-020-00440-y

Lu YZ, Liu JK, Hyde KD, Jeewon R, Kang JC, Fan C, Boonmee S, Bhat DJ, Luo ZL, Lin CG, Eungwanichayapant PD (2018) A taxonomic reassessment of *Tubeufiales* based on multi-locus phylogeny and morphology. Fungal Diversity 92: 131–344. https://doi.org/10.1007/s13225-018-0411-y

Lumbsch HT, Lindemuth R (2001) Major lineages of *Dothideomycetes* (*Ascomycota*) inferred from SSU and LSU rDNA sequences. Mycological Research 105(8): 901–908.

Matsumura M, Kato W, Hashimoto A, Takahashi YS, Shirouzu T, Tanaka K (2018) *Crassiperidium* (*Pleosporales*, *Dothideomycetes*), a new ascomycetous genus parasitic on *Fagus crenata* in Japan. Mycosphere 9(6): 13. https://doi.org/10.5943/mycosphere/9/6/13

Mugambi GK, Huhndorf SM (2009) Molecular phylogenetics of *Pleosporales*: *Melanommataceae* and *Lophiostomataceae* re-circumscribed (*Pleosporomycetidae*, *Dothideomycetes*, *Ascomycota*). Studies in Mycology 64(1): 103–121. https://doi.org/10.3114/sim.2009.64.05

Mantle PG, Hawksworth DL, Pazoutova S, Collinson LM, Rassing BR (2006) *Amorosia littoralis* gen. sp. nov., a new genus and species name for the scorpinone and caffeine-producing hyphomycete from the littoral zone in the Bahamas. Mycological Research 110(12): 1371–1378. https://doi.org/10.1016/j.mycres.2006.09.013

Matsushima T (1980) Matsushima Mycological Memoirs No. 1. Saprophytic Microfungi from Taiwan, Part 1. Hyphomycetes. Matsushima Fungus Collect., Kobe, Japan: 82.

Maharachchikumbura SS, Haituk S, Pakdeeniti P, Al-Sadi AM, Hongsanan S, Chomnunti P, Cheewangkoon R (2018) *Phaeosaccardinula coffeicola* and *Trichomerium chiangmaiensis*, two new species of *Chaetothyriales* (*Eurotiomycetes*) from Thailand. Mycosphere 9(4): 769–778. <https://doi.org/10.5943/mycosphere/9/4/5>.

Mapook A, Hyde KD, McKenzie EHC, Jones EBG, Bhat DJ, Jeewon R, Stadler M, Samarakoon MC, Malaithong M, Tanunchai B, Buscot F, Wubet T, Purahong W (2020) Taxonomic and phylogenetic contributions to fungi associated with the invasive weed *Chromolaena odorata* (Siam weed). Fungal Diversity 101: 175. <https://doi.org/10.1007/s13225-020-00444-8>.

Monkai J, Wanasinghe DN, Jeewon R, Promputtha I, Phookamsak R (2021) Morphological and phylogenetic characterization of fungi within *Bambusicolaceae*: Introducing two new species from the Greater Mekong Subregion. Mycological Progress 20(5): 721–732. <https://doi.org/10.1007/s11557-021-01694-9>.

Nag Raj TR (1993) Coelomycetous anamorphs with appendage-bearing conidia. Mycologue Publications, Waterloo, Ontario: 1–1101.

Onofri S, Zucconi L (1984) Two new species of the genus *Phialocephala*. Mycotaxon 20: 185–195.

Olivier C, Berbee ML, Shoemaker RA, Loria R (2000) Molecular phylogenetic support from ribosomal DNA sequences for origin of *Helminthosporium* from *Leptosphaeria*-like loculoascomycete ancestors. Mycologia 92(4): 736-746. https://doi.org/10.1080/00275514.2000.12061213

Piepenbring M (2006) Checklist of fungi in Panama. Preliminary version. Puente Biológico 1: 1–190.

Pitt W, Úrbez-Torres JR, Trouillas FP (2014) *Munkovalsaria donacina* from grapevines and Desert Ash in Australia. Mycosphere 5: 656–661. <https://doi.org/10.5943/mycosphere/5/5/6>.

Petrini LE (2004) A revision of the genus *Stilbohypoxylon* (*Xylariaceae*). Sydowia 56: 51–71.

Phookamsak R, Liu JK, McKenzie EHC, Manamgoda DS, Ariyawansa H, Thambugala KM, Dai DQ, Camporesi E, Chukeatirote E, Wijayawardene NN, Bahkali AH, Mortimer PE, Xu JC, Hyde KD (2014) Revision of *Phaeosphaeriaceae*. Fungal Diversity 68(1): 159–238. <https://doi.org/10.1007/s13225-014-0308-3>

Phookamsak R, Norphanphoun C, Tanaka K, Dai DQ, Luo ZL, Liu JK, Su HY, Bhat DJ, Bahkali AH, Mortimer PE, Xu JC, Hyde KD (2015) Towards a natural classification of *Astrosphaeriella*-like species; introducing *Astrosphaeriellaceae* and *Pseudoastrosphaeriellaceae* fam. nov. and *Astrosphaeriellopsis*, gen. nov. Fungal Diversity 74: 143–197. <https://doi.org/10.1007/s13225-015-0352-7>

Phookamsak R, Hyde KD, Jeewon R, Bhat DJ, Jones EBG, Maharachchikumbura SSN, Raspé O, Karunarathna SC, Wanasinghe DN, Hongsanan S, Doilom M, Tennakoon DS, Machado AR, Firmino AL, Ghosh A, Karunarathna A, Mešić A, Dutta AK, Thongbai B, Devadatha B, Norphanphoun C, Senwanna C, Wei D, Pem D, Ackah FK, Wang GN, Jiang HB, Madrid H, Lee HB, Goonasekara ID, Manawasinghe IS, Kušan I, Cano J, Gené J, Li J, Das K, Acharya K, Raj KNA, Latha KPD, Chethana KWT, He MQ, Dueñas M, Jadan M, Martín MP, Samarakoon MC, Dayarathne MC, Raza M, Park MS, Telleria MT, Chaiwan N, Matočec N, de Silva NI, Pereira OL, Singh PN, Manimohan PM, Uniyal P, Shang QJ, Bhatt RP, Perera RH, Alvarenga RLM, Nogal-Prata S, Singh SK, Vadthanarat S, Oh SY, Huang SK, Rana S, Konta S, Paloi S, Jayasiri SC, Jeon SJ, Mehmood T, Gibertoni TB, Nguyen TTT, Singh U, Thiyagaraja V, Sarma VV, Dong W, Yu XD, Lu YZ, Lim YW, Chen Y, Tkalčec Z, Zhang ZF, Luo ZL, Daranagama DA, Thambugala KM, Tibpromma S, Camporesi E, Bulgakov TS, Dissanayake AJ, Senanayake IC, Dai DQ, Tang LZ, Khan S, Zhang H, Promputtha I, Cai L, Chomnunti P, Zhao RL, Lumyong S, Boonmee S, Wen TC, Mortimer PE, Xu JC (2019) Fungal diversity notes 929–1035: taxonomic and phylogenetic contributions on genera and species of fungi. Fungal Diversity 95: 1–273. <https://doi.org/10.1007/s13225-019-00421-w>

Phukhamsakda C, Bhat DJ, Hongsanan S, Xu JC, Stadler M, Hyde KD (2018) Two novel species of *Neoaquastroma* (*Parabambusicolaceae*, *Pl*eosporales) with their *phoma*-like asexual morphs. MycoKeys 34: 47. <https://doi.org/10.3897/mycokeys.34.25124>

Phukhamsakda C, McKenzie EHC, Phillips AJL, Jones EBG, Bhat DJ, Stadler M, Bhunjun CS, Wanasinghe DN, Thongbai B, Camporesi E, Ertz D, Jayawardena RS, Perera RH, Ekanayake AH, Tibpromma S, Doilom M, Xu JC, Hyde KD (2020) Microfungi associated with *Clematis* (*Ranunculaceae*) with an integrated approach to delimiting species boundaries. Fungal Diversity 102: 1–203. <https://doi.org/10.1007/s13225-020-00448-4>

Poli A, Bovio E, Ranieri L, Varese GC, Prigione V (2020) News from the sea: a new genus and seven new species in the *Pleosporalean* families *Roussoellaceae* and *Thyridariaceae*. Diversity 12(4): 144. <https://doi.org/10.3390/d12040144>

Panno L, Bruno M, Voyron S, Anastasi A, Gnavi G, Miserere L, Varese GC (2013) Diversity, ecological role and potential biotechnological applications of marine fungi associated to the seagrass *Posidonia oceanica*. New Biotechnology 30(6): 685–694. <https://doi.org/10.1016/j.nbt.2013.01.010>

Quaedvlieg WGJM, Verkley GJM, Shin HD, Barreto RW, Alfenas AC, Swart WJ, Crous PW (2013) Sizing up septoria. Studies in Mycology 75(1): 307–390. https://doi.org/10.3114/sim0017

Ren GC, Wanasinghe DN, Monkai J, Mortimer PE, Hyde KD, Xu JC, Pang A, Gui H (2021) Novel saprobic *Hermatomyces* species (*Hermatomycetaceae*, *Pleosporales*) from China (Yunnan Province) and Thailand. MycoKeys 82: 57–79. https://doi.org/10.3897/mycokeys.82.67973

Raja HA, Tanaka K, Hirayama K, Miller AN, Shearer CA (2011) Freshwater ascomycetes: two new species of *Lindgomyces* (*Lindgomycetaceae*, *Pleosporales*, *Dothideomycetes*) from Japan and USA. Mycologia 103(6): 1421–1432. <https://doi.org/10.3852/11-077>

Raja HA, Oberlies NH, El-Elimat T, Miller AN, Zelski SE, Shearer CA (2013) *Lindgomyces angustiascus*, (*Lindgomycetaceae*, *Pleosporales*, *Dothideomycetes*), a new lignicolous species from freshwater habitats in the USA. *Mycoscience* 54(5): 353–361. <https://doi.org/10.1016/j.myc.2012.12.004>

Raja HA, Paguigan ND, Fournier J, Oberlies NH (2017) Additions to *Lindgomyces* (*Lindgomycetaceae*, *Pleosporales*, *Dothideomycetes*), including two new species occurring on submerged wood from North Carolina, USA, with notes on secondary metabolite profiles. Mycological Progress 16: 535–552. <https://doi.org/10.1007/s11557-017-1282-4>

Rehner S, Buckley E (2005) Cryptic diversification in *Beauveria bassiana* inferred from nuclear its and *tef*1-alpha phylogenies. Mycologia 97: 84–98. <https://doi.org/10.3852/mycologia.97.1.84>.

Rathnayaka AR, Dayarathne MC, Maharachchikumbura SSN, Liu JK, Tennakoon DS, Hyde KD (2019) Introducing *Seriascoma yunnanense* sp. nov. (*Occultibambusaceae*, *Pleosporales*) based on evidence from morphology and phylogeny. Asian Journal of Mycology 2: 245–253. <https://doi.org/10.5943/ajom/2/1/15>

Schoch CL, Shoemaker RA, Seifert KA, Hambleton S, Spatafora JW, Crous PW (2006) A multigene phylogeny of the *Dothideomycetes* using four nuclear loci. Mycologia 98(6): 1041–1052. <https://doi.org/10.1080/15572536.2006.11832632>

Senwanna C, Wanasinghe DN, Bulgakov TS, Wang Y, Bhat DJ, Tang AMC, Mortimer PE, Xu J, Hyde KD, Phookamsak R (2019) Towards a natural classification of *Dothidotthia* and *Thyrostroma* in *Dothidotthiaceae* (*Pleosporineae*, *Pleosporales*). Mycosphere 10(1): 701. <https://doi.org/10.5943/mycosphere/10/1/15>

Spatafora JW, Sung GH, Johnson D, Hesse C, O’Rourke B, Serdani M, Spotts R, Lutzoni F, Hofstetter V, Miadlikowska J, Reeb V, Gueidan C, Fraker E, Lumbsch T, Lücking R, Schmitt I, Hosaka K, Aptroot A, Roux C, Miller AN, Geiser DM, Hafellner J, Hestmark G, Arnold AE, Büdel B, Rauhut A, Hewitt D, Untereiner WA, Cole MS, Scheidegger C, Schultz M, Sipman H, Schoch CL (2006) A five-gene phylogeny of *Pezizomycotina*. *Mycologia* 98(6): 1018–1028. <https://doi.org/10.1080/15572536.2006.11832630>

Stadler M, Læssøe T, Fournier J, Decock C, Schmieschek B, Tichy HV, Peršoh D (2014) A polyphasic taxonomy of *Daldinia* (*Xylariaceae*). Studies in Mycology 77: 1–143. <https://doi.org/10.3114/sim0016>

Suetrong S, Schoch CL, Spatafora JW, Kohlmeyer J, Volkmann-Kohlmeyer B, Sakayaroj J, et al. (2009) Molecular systematics of the marine *Dothideomycetes*. Studies in Mycology 64(1): 155–173. <https://doi.org/10.3114/sim.2009.64.09>

Schoch CL, Crous PW, Groenewald JZ, Boehm EWA, Burgess TI, De Gruyter J, Spatafora JW (2009) A class-wide phylogenetic assessment of *Dothideomycetes*. Studies in Mycology 64(1): 1–15. <https://doi.org/10.3114/sim.2009.64.01>

Sheir-Neiss G, Lai MH, Morris NR (1978) Identification of a gene for β-tubulin in *Aspergillus nidulans*. Cell 15: 639–647. https://doi.org/10.1016/0092-8674(78)90032-6.

Samarakoon BC, Phookamsak R, Wanasinghe DN, Chomnunti P, Hyde KD, McKenzie EH, Promputtha I, Xu JC, Li YJ (2020) Taxonomy and phylogenetic appraisal of *Spegazzinia musae* sp. nov. and *S. deightonii* (*Didymosphaeriaceae*, *Pleosporales*) on *Musaceae* from Thailand. MycoKeys 70: 19–37. https://doi.org/10.3897/mycokeys.70.52043

Spatafora JW, Sung GH, Johnson D, Hesse C, O’Rourke B, Serdani M, Schoch CL (2006) A five-gene phylogeny of *Pezizomycotina*. Mycologia 98(6): 1018–1028. <https://doi.org/10.1080/15572536.2006.11832630>

Sun YR, Zhang JY, Hyde KD, Wang Y, Jayawardena RS (2023) Morphology and phylogeny reveal three *Montagnula* species from China and Thailand. Plants 12(4): e738. https://doi.org/10.3390/plants12040738

Tanaka K, Hirayama K, Yonezawa H, Sato G, Toriyabe A, Kudo H, Hosoya T (2015) Revision of the *Massarineae* (*Pleosporales*, *Dothideomycetes*). Studies in Mycology 82: 75–136. <https://doi.org/10.1016/j.simyco.2015.10.002>

Tanaka K, Hirayama K, Yonezawa H, Hatakeyama S, Harada Y, Sano T, Hosoya T (2009) Molecular taxonomy of bambusicolous fungi: *Tetraplosphaeriaceae*, a new *pleosporalean* family with *Tetraploa*-like anamorphs. Studies in Mycology 64(1): 175–209. <https://doi.org/10.3114/sim.2009.64.10>

Tanaka K, Hosoya T (2008) *Lophiostoma sagittiforme* sp. nov., (*Pleosporales*, *Dothideomycetes*) from Island Yakushima in Japan. Sydowia 60(1): 131–145.

Thambugala KM, Hyde KD, Tanaka K, Tian Q, Wanasinghe DN, Ariyawansa HA, Jayasiri SC, Boonmee S, Camporesi E, Hashimoto A, Hirayama K, Schumacher RK, Promputtha I, Liu ZY (2015) Towards a natural classification and backbone tree for *Lophiostomataceae*, *Floricolaceae*, and *Amorosiaceae* fam. nov. Fungal Diversity 74: 199–266. <https://doi.org/10.1007/s13225-015-0348-3>

Thambugala KM, Wanasinghe DN, Phillips AJL, Camporesi E, Bulgakov TS, Phukhamsakda C, Ariyawansa HA, Goonasekara ID, Phookamsak R, Dissanayake A, Tennakoon DS, Tibpromma S, Chen YY, Liu ZY, Hyde KD (2017) Mycosphere notes 1–50: grass (*Poaceae*) inhabiting *Dothideomycetes*. Mycosphere 8(4): 697–796. <https://doi.org/10.5943/mycosphere/8/4/13>

Tennakoon DS, Hyde KD, Wanasinghe DN, Bahkali AH, Camporesi E, Khan S, Phookamsak R (2016) Taxonomy and phylogenetic appraisal of *Montagnula jonesii* sp. nov. (*Didymosphaeriaceae*, *Pleosporales*). Mycosphere 7: 1346–1356. https://doi.org/10.5943/mycosphere/7/9/8

Tennakoon DS, Thambugala KM, de Silva NI, Suwannarach N, Lumyong S (2022) A taxonomic assessment of novel and remarkable fungal species in *Didymosphaeriaceae* (*Pleosporales*, *Dothideomycetes*) from plant litter. Frontiers in Microbiology 13: e1016285. https://doi.org/10.3389/fmicb.2022.1016285

Tibpromma S, Hyde KD, Jeewon R, Maharachchikumbura SSN, Liu JK, Bhat DJ, Jones EBG, McKenzie EHC, Camporesi E, Bulgakov TS, Doilom M, Santiago ALCMA, Das K, Manimohan P, Gibertoni TB, Lim YW, Ekanayaka AH, Thongbai B, Lee HB, Yang JB, Kirk PM, Sysouphanthong P, Singh SK, Boonmee S, Dong W, Raj KNA, Latha KPD, Phookamsak R, Phukhamsakda C, Konta S, Jayasiri SC, Norphanphoun C, Tennakoon DS, Li J, Dayarathne MC, Perera RH, Xiao Y, Wanasinghe DN, Senanayake IC, Goonasekara ID, de Silva NI, Mapook A, Jayawardena RS, Dissanayake AJ, Manawasinghe IS, Chethana KW, Luo ZL, Hapuarachchi KK, Baghela A, Soares AM, Vizzini A, Meiras-Ottoni A, Mešić A, Dutta AK, Souza CAFd, Richter C, Lin CG, Chakrabarty D, Daranagama DA, Lima DX, Chakraborty D, Ercole E, Wu F, Simonini G, Vasquez G, Alves da Silva GA, Plautz Jr HL, Ariyawansa HA, Lee H, Kušan I, Song J, Sun J, Karmakar J, Hu KF, Semwal KC, Thambugala KM, Voigt K, Acharya K, Rajeshkumar K, Ryvarden L, Jadan M, Hosen MI, Mikšík M, Samarakoon MC, Wijayawardene NN, Kim NK, Matočec N, Singh PNS, Tian Q, Bhatt RP, Oliveira RJVd, Tulloss RE, Aamir S, Kaewchai S, Marathe SD, Khan S, Hongsanan S, Adhikari S, Mehmood T, Bandyopadhyay TK, Svetasheva TY, Nguyen TTT, Antonín V, Li WJ, Wang Y, Indoliya Y, Tkalčec Z, Elgorban AM, Bahkali AH, Tang AMC, Su HY, Zhang H, Promputtha I, Luangsa-ard J, Xu J, Yan J, Ji-Chuan K, Stadler M, Mortimer PE, Chomnunti P, Zhao Q, Phillips AJL, Nontachaiyapoom S, Wen TC, Karunarathna SC (2017) Fungal diversity notes 491–602: taxonomic and phylogenetic contributions to fungal taxa. Fungal Diversity 83: 1–261. <https://doi.org/10.1007/s13225-017-0378-0>

Tibpromma S, Hyde KD, McKenzie EH, Bhat JD, Phillips AJL, Wanasinghe DN, Samarakoon MC, Jayawardena RS, Dissanayake AJ, Tennakoon DS, Doilom M, Phookamsak R, Tang AMC, Xu JC, Mortimer PE, Promputtha I, Maharachchikumbura SSN, Khan S, Karunarathna SC (2018) Fungal Diversity notes 840–928: micro-fungi associated with *Pandanaceae*. Fungal Diversity 93: 1–160. https://doi.org/10.1007/s13225-018-0408-6

Urtiaga R (1986) Indice de enfermedades en plantas de Venezuela y Cuba. Impresos Nuevo Siglo. S.R.L., Barquisimeto, Venezuela: 202.

Vincent MA, Seifert KA, Samson RA (1988) *Akanthomyces johnsonii*, a saprophytic synnematous hyphomycete. Mycologia 80: 685–688. <https://doi.org/10.1080/00275514.1988.12025601>

Vilgalys R, Hester M (1990) Rapid genetic identification and mapping of enzymatically amplified ribosomal DNA from several *Cryptococcus* species. Journal of Bacteriology 172: 4238–4246. <https://doi.org/10.1128/jb.172.8.4238-4246.1990>.

Voglmayr H, Jaklitsch WM (2017) *Corynespora*, *Exosporium* and *Helminthosporium* revisited-new species and generic reclassification. Studies in Mycology 87(1): 43–76. https://doi.org/10.1016/j.simyco.2017.05.001

Valenzuela-Lopez N, Sutton DA, Cano-Lira JF, Paredes K, Wiederhold N, Guarro J, Stchigel AM (2017) Coelomycetous fungi in the clinical setting: morphological convergence and cryptic diversity. Journal of Clinical Microbiology 55(2): 552–567. <https://doi.org/10.1128/jcm.02221-16>

Voglmayr H, Jaklitsch WM (2011) Molecular data reveal high host specificity in the phylogenetically isolated genus *Massaria* (*Ascomycota*, *Massariaceae*). Fungal Diversity 46: 133–170. <https://doi.org/10.1007/s13225-010-0078-5>

White TJ, Bruns T, Lee S, Taylor J (1990) Amplification and direct sequencing of fungal ribosomal RNA genes for phylogenetics. In: PCR Protocols: A Guide to Methods and Applications 18: 315–322. https://doi.org/10.1016/B978-0-12-372180-8.50042-1.

Wanasinghe DN, Jones EBG, Camporesi E, Dissanayake AJ, Kamolhan S, Mortimer PE, Xu J, Abd-Elsalam KA, Hyde KD (2016a) Taxonomy and phylogeny of *Laburnicola* gen. nov. and *Paramassariosphaeria* gen. nov. (*Didymosphaeriaceae*, *Massarineae*, *Pleosporales*). Fungal Biology 120: 1354–1373. <https://doi.org/10.1016/j.funbio.2016.06.006>

Wanasinghe DN, Jones EBG, Dissanayake AJ, Hyde KD (2016b) Saprobic *Dothideomycetes* in Thailand: *Vaginatispora appendiculata* sp. nov. (*Lophiostomataceae*) introduced based on morphological and molecular data. Studies in Fungi 1(1): 56–68.

Wanasinghe DN, Phukhamsakda C, Hyde KD, Jeewon R, Lee HB, Jones EBG, Tibpromma S, Tennakoon DS, Dissanayake AJ, Jayasiri SC, Gafforov Y, Camporesi E, Bulgakov TS, Ekanayake AH, Perera RH, Samarakoon MC, Goonasekara ID, Mapook A, Li WJ, Senanayake IC, Li J, Norphanphoun C, Doilom M, Bahkali AH, Xu J, Mortimer PE, Tibell L, Tibell S, Karunarathna SC (2018) Fungal diversity notes 709–839: taxonomic and phylogenetic contributions to fungal taxa with an emphasis on fungi on *Rosaceae*. Fungal Diversity 89: 1–236. <https://doi.org/10.1007/s13225-018-0395-7>

Wanasinghe DN, Mortimer PE (2022) Taxonomic and phylogenetic insights into novel *Ascomycota* from forest woody litter. Biology 11(6): 889. https://doi.org/10.3390/biology11060889

Wanasinghe DN, Wijayawardene NN, Xu J, Cheewangkoon R, Mortimer PE (2020) Taxonomic novelties in *Magnolia*-associated *pleosporalean* fungi in the Kunming Botanical Gardens (Yunnan, China). PLOS ONE 15(7): e0235855. <https://doi.org/10.1371/journal.pone.0235855>

Wanasinghe DN, Mortimer PE, Xu J (2021) Insight into the systematics of microfungi colonizing dead woody twigs of *Dodonaea viscosa* in Honghe (China). Journal of Fungi 7(3): 180. <https://doi.org/10.3390/jof7030180>

Wei GY, Mo WD, Hu Y, Zou MT, Wen JT, Chang LF, Yang QI (2024) Discovery of a novel *Occultibambusa* species (*Occultibambusaceae*, Dothideomycetes) from *Poaceae* host from Guizhou Province, China. Current Research in Environmental & Applied Mycology (Journal of Fungal Biology) 14(1): 146–156.

Wijayawardene NN, Dai DQ, Zhu ML, Wanasinghe DN, Kumla J, Zhang GQ, Chen HH (2022) Fungi associated with dead branches of *Magnolia grandiflora*: A case study from Qujing, China. Frontiers in Microbiology 13: 954680. <https://doi.org/10.3389/fmicb.2022.954680>

Wijesinghe SN, Samarakoon MC, Camporesi E, Hyde KD, Jones EBG, Zucconi L, Perera RH, Boonmee S, de Silva NI, Rathnayaka AR, Pem D, Wanasinghe DN, Maharachchikumbura SSN, Wang Y (2023) Over the footprints of Italian mycology with emphasis on plant-associated Ascomycota. Current Research in Environmental & Applied Mycology 13: 162–276. <https://doi.org/10.5943/cream/13/1/11>

Wang XJ, Wu HY, Zhang M (2014) A new species of *Helminthosporium* from Jiangsu, China. Mycotaxon 127(1): 1–4. https://doi.org/10.5248/127.1

Wijesinghe SN, Wang Y, Camporesi E, Wanasinghe DN, Boonmee S, Hyde KD (2020a) A new genus of *Bambusicolaceae* (*Pleosporales*) on *Corylus avellana* (*Fagales*) from Italy. Biodiversity Data Journal 8. <https://doi.org/10.3897/BDJ.8.e55957>

Wijesinghe SN, Wanasinghe DN, Maharachchikumbura SS, Wang Y, Al-Sadi AM, Hyde KD (2020b) *Bimuria omanensis* sp. nov. (*Didymosphaeriaceae*, *Pleosporales*) from *Oman*. Phytotaxa 449(2): 97–108. <https://doi.org/10.11646/phytotaxa.449.2.1>

Wang F, Zeng Q, Lv Y, Xu X, Han S, Yang H, Yang C (2022) Branch blight of Juglans regia caused by *Palmiascoma qujingense* in China. Plant Disease 106(11): 2992. <https://doi.org/10.1094/PDIS-01-22-0010-PDN>

Xu S, Phookamsak R, Jiang HB, Tibpromma S, Yang JB, Xu JC, Lumyong S (2022) First report of *Occultibambusa jonesii* on Para grass (*Brachiaria mutica*) in Yunnan, China. World 4: 19. <https://doi.org/10.12982/CMJS.2022.048>

Yu XD, Zhang SN, Liu JK (2022) Morpho-phylogenetic evidence reveals novel *pleosporalean* taxa from Sichuan Province, China. Journal of Fungi 8(7): 720. <https://doi.org/10.3390/jof8070720>

Yang EF, Tibpromma S, Karunarathna SC, Phookamsak R, Xu JC, Zhao ZX, Promputtha I (2022) Taxonomy and phylogeny of novel and extant taxa in *Pleosporales* associated with *Mangifera indica* from Yunnan, China (Series I). Journal of Fungi 8(2): 152. <https://doi.org/10.3390/jof8020152>

Yang J, Liu LL, Jones EBG, Hyde KD, Liu ZY, Bao DF, Liu NG, Li WL, Shen HW, Yu XD, Liu JK (2023) Freshwater fungi from karst landscapes in China and Thailand. Fungal Diversity 119(1): 1–212. <https://doi.org/10.1007/s13225-023-00514-7>

Yu XD, Zhang SN, Cheewangkoon R, Liu JK (2021) Additions to *Occultibambusaceae* (*Pleosporales*, *Dothideomycetes*): Unrevealing palmicolous fungi in China. Diversity 13(11): 516. <https://doi.org/10.3390/d13110516>

Zhao ZZ, Zhao K, Chen HP, Bai X, Zhang L, Liu JK (2017) Terpenoids from the mushroom-associated fungus *Montagnula donacina*. Phytochemistry 147: 21–29. https://doi.org/10.1016/j.phytochem.2017.12.015

Zeng Y, Yang M, Xu J, Luo M, Guo Q, Jiang Y (2024) First report of leaf spot caused by *Pseudopithomyces chartarum* on Chinese prickly ash in China. Crop Protection 182: 106712. <https://doi.org/10.1016/j.cropro.2024.106712>

Zhu D, Luo ZL, Baht DJ, McKenzie EH, Bahkali AH, Zhou DQ, Hyde KD (2016) *Helminthosporium velutinum* and *H. aquaticum* sp. nov. from aquatic habitats in Yunnan Province, China. Phytotaxa 253(3): 179–190. https://doi.org/10.11646/phytotaxa.253.3.1

Zhao N, Luo ZL, D Hyde KE, Su HY, Bhat DJ, Liu JK, Hao YE (2018) *Helminthosporium submersum* sp. nov. (*Massarinaceae*) from submerged wood in north-western Yunnan Province, China. Phytotaxa 348(4): 269–278. https://doi.org/10.11646/phytotaxa.348.4.3

Zhang JF, Liu JK, Hyde KD, Yang W, Liu ZY (2017) Fungi from Asian karst formations II. Two new species of *Occultibambusa* (*Occultibambusaceae*, *Dothideomycetes*) from karst landforms of China. Mycosphere 8(4): 550–559. <https://doi.org/10.5943/mycosphere/8/4/4>

Zhang JZ, Phookamsak R, Boonmee S, Hyde KD, Dai DQ, Lu YZ (2020) *Roussoella guttulata* (*Roussoellaceae*, *Pleosporales*), a novel bambusicolous ascomycete from Thailand. Phytotaxa 471(3): 221–233. <https://doi.org/10.11646/phytotaxa.471.3.4>

Zhang SN, Hyde KD, Gareth Jones EB, et al. (2018) *Acuminatispora palmarum* gen. et sp. nov. from mangrove habitats. Mycological Progress 17: 1173–1188. <https://doi.org/10.1007/s11557-018-1433-2>

Zhang W, Groenewald JZ, Lombard L, Schumacher RK, Phillips AJL, Crous PW (2021) Evaluating species in *Botryosphaeriales*. Persoonia – Molecular Phylogeny and Evolution of Fungi 46(1): 63–115. <https://doi.org/10.3767/persoonia.2021.46.03>

Zhang Y, Wang HK, Fournier J, Crous PW, Jeewon R, Pointing SB, Hyde KD (2009) Towards a phylogenetic clarification of *Lophiostoma* / *Massarina* and morphologically similar genera in *Pleosporales*. Fungal Diversity 38: 225–251.
